# Perinatal depression and adverse child growth outcomes in low-income and middle-income countries (LMICs): A systematic review and meta-analysis

**DOI:** 10.1371/journal.pgph.0003586

**Published:** 2024-10-28

**Authors:** Elizabeth Carosella, Shradha Chhabria, Hyelee Kim, Aliya Moreira, Dana Naamani, Brennan Ninesling, Aimee Lansdale, Lakshmi Gopalakrishnan, Bizu Gelaye, Aisha Yousafzai, Stefania Papatheodorou

**Affiliations:** 1 Harvard T.H. Chan School of Public Health, Boston, Massachusetts, United States of America; 2 University of California San Francisco, San Francisco, California, United States of America; 3 Rutgers School of Public Health, Piscataway, New Jersey, United States of America; VART Consulting PVT LTD, INDIA

## Abstract

Perinatal depression (PND), which encompasses the antepartum and postpartum depression (APD and PPD), is a neglected crisis in low-income and middle-income countries (LMICs). We aimed to systematically search and meta-analyze existing evidence to determine whether a mother’s PND affects adverse growth outcomes in children in LMICs (PROSPERO protocol: CRD42021246803). We conducted searches, including nine databases (PubMed, EMBASE, Web of Science, CINAHL Plus, Global Health Database, Google Scholar, WHO Regional Databases, PsycINFO, and LILACS) from January 2000 to September 2023. We restricted studies that assessed PND using validated screening tools or clinical interviews during pregnancy or within 12 months postpartum. We included studies that reported four types of adverse child growth outcomes (stunting, wasting, underweight, and overweight/obesity) in children younger than 5 years. We assessed the quality using the Newcastle Ottawa Scale and pooled risk ratios (RRs) and odds ratios (ORs) between PND and each adverse growth outcome using random-effects models. In total, 27 studies met the inclusion criteria for systematic review, with 24 eligible for meta-analysis, spanning data from 15 countries and 26,261 mother-baby pairs. Based on the studies that reported ORs, children below the age of 3 years with mothers experiencing PND had higher odds for stunting (OR 1.63, 95% CI 1.32, 2.02, I^2^ = 56.0%) and underweight (OR 2.65, 95% CI 1.90, 3.68, I^2^ = 34.5%) compared to children of mothers without PND. The pooled RRs for stunting and underweight did not show significant differences between mothers with and without PND. Studies on wasting (n = 5) and overweight/obesity (n = 2) were limited, demonstrating inconsistent results across studies. The association between PND and adverse growth outcomes varied according to the measure of association, region, country, PND type, outcome timepoint, and study design. There were limited studies in diverse LMICs, particularly on wasting, or overweight/obesity as an outcome.

## Introduction

Perinatal depression (PND), a nonpsychotic depressive episode of mild to major severity, affects 11.9% of mothers during pregnancy or after delivery worldwide [1], with significant reductions in quality of life and day-to-day functioning, with potentially far-reaching consequences for ranges of adverse child health outcomes [2, 3]. PND encompasses depression among mothers during pregnancy, antepartum depression (APD), and depression after delivery, postpartum depression (PPD), depending on the timing of symptom onset [4, 5]. Research has suggested a higher prevalence of PND in low-income and middle-income countries (LMICs) compared to high-income countries where epidemiologic estimates are well studied [1, 6], and in LMICs, over 25% and 19% of women suffer from PND and PPD, respectively [7].

Maternal health during and after pregnancy plays a critical role in children’s health. The first 1,000 days from conception to two years of age is a particularly sensitive period for children’s development and sets the stage for their future growth trajectories. Moreover, eating behaviors and food preferences, influenced by parental care practices, become established during the first five years [8, 9]. A systematic review and meta-analysis in Africa highlighted a strong association between APD and adverse birth outcomes, such as preterm birth and low birth weight, while PPD was also highly associated with infant growth and health outcomes [10]. Further, studies have shown that PPD in LMICs could contribute to poor infant attachment and interactions with caregivers, potentially contributing to developmental and emotional challenges in children’s future [6]. Inadequate nutrition during this time may lead to negative health outcomes for children, including cognitive impairment, compromised immune systems, and an increased risk of cardiometabolic disease [11–13].

Globally, nearly half of child deaths are associated with malnutrition [14]. According to the 2021 UNICEF/WHO/World Bank Group Joint Child Malnutrition Estimates, 149.2 million children below five years were affected by stunting, 45.4 million by wasting, and 28.9 million by overweight [15]. Despite progress in recent decades, stunting and wasting rates remain above-established targets, while the prevalence of overweight is on the rise [16]. Neglecting to address PND may be contributing to these trends, with severe consequences for children’s health, development, and future economic participation [17]. Moreover, the COVID-19 pandemic is expected to heighten the risk of all forms of child malnutrition and poor maternal mental health [18, 19]. As part of efforts to achieve Sustainable Development Goal 2 and end all forms of malnutrition by 2030 [20], maternal mental health merits higher priority on the global public health agenda. Nonetheless, there is relatively less evidence regarding the relationship between perinatal maternal mental health and child adverse growth outcomes in LMICs [21, 22]. A synthesis of the available evidence for this relationship is useful for understanding these relationships and for highlighting gaps in the existing literature.

The objective of this systematic review and meta-analysis was to assess whether PND has an impact on four types of adverse growth outcomes (stunting, wasting, underweight, overweight/obesity) among children under five years in LMICs using existing studies. While we considered the associations reported in the previous studies, our primary interest lies in understanding the temporal relationship between PND and adverse child growth outcomes. Thus, we hypothesized that the PND would exhibit a statistically significant association with adverse child growth outcomes in the pooled analyses. In contrast to previous meta-analyses on this topic, we intended to estimate the effect of exposures to APD and PPD separately or as a composite PND on a broader range of adverse growth outcomes, including wasting and overweight/obesity.

## Methods

We defined PND as a diagnosis made during pregnancy or within 12 months postpartum. Our primary exposure of interest was PND. Therefore, in cases where the authors reported maternal depression instead of PND, we limited the measurement time point for PPD to a period of up to 12 months after delivery to prevent reverse causation between the exposure, PPD, and the outcome. We reported the pooled odds ratios (ORs) and the pooled risk ratios (RRs) for child adverse growth outcomes for both disaggregated exposures (APD and PPD) and the composite exposure (PND) [4]. Given that each growth outcome has unique environmental and biological underpinnings, our aim was to report multiple disaggregated adverse growth outcomes, specifically stunting, underweight, wasting, and overweight/obesity. This study adhered to the pre-registered protocol on PROSPERO International prospective register of systematic reviews (registration number: CRD42021246803) [23].

Stunting was defined as height or length-for-age z-scores (HAZ or LAZ) of two standard deviations (SD) or more below the WHO Child Growth Standards median by sex [24]. Underweight was defined as weight-for-age z-scores (WAZ) of two SDs or more below the WHO Child Growth Standards median by sex [24]. Wasting was defined as weight-for-length or height-for-length z-scores (WLZ or HLZ) of two SDs or more below the WHO Child Growth Standards median by sex [24]. Overweight was defined as WLZ or HLZ of two SDs or more from the WHO Child Growth Standards median by sex, and obesity was defined as WLZ or HLZ of three SDs or more from the WHO Child Growth Standards median by sex [24]. Given the nature of the systematic review, the institutional review board approval was not required.

### Data source

We conducted searches in the following databases: PubMed, EMBASE, Web of Science, CINAHL Plus, Global Health Database (EBSCO), Google Scholar, WHO Regional Databases (Global Index Medicus), PsycINFO, and Latin American and Caribbean Health Sciences Literature (LILACS). Our initial search was from January 2000 to March 2021, and during the revision process, we updated the search until September 2023 to include the recent publications for the past two years. The exact search terms used for each of the databases are provided in S1 Text. Additionally, we supplemented our search with a hand search of the Women’s Mental Health Journal, Conference abstracts of the International Marcé Society for Perinatal Mental Health, the WHO working group on PND, and related information and studies from websites of the Center for Global Mental Health and ReliefWeb.

### Study selection and data extraction

Identified references were uploaded to Covidence.org for the review process [25]. Eight reviewers completed title and abstract screening, full-text review, and data extraction (E.C., H.K., B.N., S.C., D.N., A.M., A.L., and L.G.). Each title and abstract, and subsequently each full-text study, underwent reviewed by a combination of two authors among the list (E.C., H.K., B.N., S.C., D.N., A.M., A.L., and L.G.). Articles agreed by two authors were included in the study. For articles selected by only one author, the inclusion was based on the team’s discussion of its suitability for inclusion.

Studies were considered eligible if they met the following criteria: 1) was an original research article published in a peer-reviewed journal; 2) contained demographic and clinical information on mother-baby pairs; 3) the exposure was either APD or PPD, diagnosed during pregnancy or up to 12 months after delivery; 4) depression diagnosis was based on a validated patient questionnaire or clinical interview; 5) the outcome was classified as stunting, wasting, underweight, overweight, or obesity among children under age five; 6) conducted in an LMIC as defined by the World Bank in 2021, including 136 low-, lower-middle, or upper-middle income countries [26]; 7) reported relevant quantitative measures, such as ORs, RRs, or other metrics suitable for meta-analysis (e.g., % proportion or beta-coefficients); and 8) the publication was available in English, Spanish, Portuguese, or French.

We excluded studies that were literature reviews, systematic reviews and/or meta-analyses, and clinical trials. When a study conducted secondary data analysis on data from a randomized controlled trial (RCT) as an observational study, we assumed that the path from randomization to PND did not exist due to the inclusion of randomization. Consequently, observational studies conducted within RCTs were included if there was no backdoor path between PND and adverse growth outcomes. If the studies used PND measurement addressing heterogeneous symptoms of depression, anxiety, and psychological distress, we included them if the association for depression was reported or if the authors demonstrated that the measure was validated for depression in the study population. In cases where adverse growth outcomes were defined outside the inclusion criteria, we included the studies based on consensus. Studies were excluded if they solely assessed adverse birth outcomes (e.g. low birth weight) or if the definition of adverse growth outcomes was omitted in the paper.

Since our research question focuses on adverse growth outcomes, we excluded studies that analyzed the continuous growth outcome indicators, such as height-/length-for-age Z score (HAZ/LAZ), weight-for-age Z score (WAZ), or height-/length-for-weight Z score (HWZ/LWZ), as opposed to adverse growth outcomes (binary variable). Additionally, during the full-text review, it became apparent that many studies examined the association between maternal depression outside the PND timeframe (e.g. maternal depression measured four years after delivery) and child growth outcomes that were not classified as adverse. While these findings were not the primary focus of our research, we have included a summary in the S1 Table due to their relevance to our main research question. This table includes 30 studies, which including additional findings from four studies within the final selection for systematic review and meta-analysis [27–56].

Standardized data collection forms were developed by two authors (B.N. and E.C.), and data extraction was performed by a combination of two authors among eight authors (D.N., H.K., S.C., A.M., B.N., E.C., A.L., and L.G) during May 2021 for the first search and October 2023 for the search update. The data extracted included the following variables: authors, publication year, country, study design, number of mother-child pairs, PND measurements, and their cut-points and timing, the type and definition of adverse growth outcomes, including the outcome timepoint, covariates, measures of association (types and values), and 95% confidence intervals (CIs).

### Quality assessment

The quality of the included studies was assessed by five authors (B.N., D.N., H.K., A.L., and L.G.), who evaluated studies using the Newcastle Ottawa Scale (NOS) (S2 Table) [57]. Each study underwent assessment by two reviewers (D.N. and H.K.), and a third reviewer (B.N., A.L., or L.G.) provided the final approval and acted as a tiebreaker in the case of any discordance. One component of quality assessment for cohort studies examined whether the outcome of interest was absent at the baseline. However, given our research focus on the path from APD through adverse birth outcomes, we decided that this requirement was irrelevant to our research question. Consequently, we did not deduct any points for this component, regardless of whether the outcome was reported at baseline.

Studies with a score of 7 or higher out of a total score of 9 were categorized as "good quality." Nearly all studies, except for Joshi and Raut (2019), got scores of seven or above on the NOS scale, demonstrating sufficient quality for inclusion in the study. It is worth noting that while these studies were deemed to have good quality, each study may not have been entirely free from bias due to various factors, such as missing observations, residual and unmeasured confounding, and measurement bias. Joshi and Raut (2019) received a score of 5 due to the absence of attrition rate reporting and the use of unadjusted reported measures of association. Regardless, we confirmed that all studies are eligible to be included in the review.

### Synthesis

While our initial aim was to perform meta-analyses encompassing children up to 5 years, it was observed that nearly all the included studies assessed growth outcomes in children aged less than 32 months. The exception was a single study, Bennett et al. (2016), which included four sub-studies and reported the outcomes at the age of five years [58]. In cases where outcomes were repeatedly measured in a longitudinal cohort study, the associations measured at different time points were inherently correlated since they were measured in the same individual. Consequently, including all of these estimates in the meta-analysis would have artificially reduced the CIs [59]. To address this, we selected one of the estimates in such cases [59]. To ensure homogeneity in the timepoints of outcomes, the primary meta-analysis focused on estimates reported closest to the one-year mark. For example, in Patel et al. (2003), we selected the adjusted OR (aOR) among children aged six months rather than 6 weeks. For estimates repeated at various time points, we conducted the subgroup analyses to understand the trend of the association based on the outcome timepoints.

Due to the limited number of studies using a consistent measure of association for overweight/obesity, the primary meta-analysis focused only on stunting, underweight, and wasting. Additionally, recognizing the heterogeneity, we stratified the analysis based on the type of measure of association (RR and OR). Meta-analyses were conducted using both the DerSimonian and Laird methods (DL) under a random-effects model and the Knapp–Hartung (KH) standard-error adjustment estimates (also known as Sidik–Jonkman adjustment), given the relatively small number of studies for each outcome [60, 61]. We presented the results of KH estimates in the S3 Table. We reported the overall effect of PND on each adverse growth outcome and performed subgroup analyses based on the timing of depression diagnosis (APD vs. PPD), time point of outcome (4 months, 6 months, 1 year, 2 years, and 5 years), region (Africa, America, Asia, and Middle-East), and study design (case-control, cohort, cross-sectional, and secondary analysis in RCT).

Sensitivity analyses were conducted to assess the influence of 1) unadjusted estimates on the pooled estimate, 2) inclusion of maternal depression measured up to 24 months, and 3) studies published more than ten years ago. Funnel plots and Egger’s test were conducted to examine potential publication bias due to missing studies [62]. To evaluate heterogeneity, we followed the guidelines recommended by the Cochrane Statistical Methods Group: *I*^2^ 30% to 60% moderate heterogeneity, 50% to 90% substantial heterogeneity, and 75% to 100% indicating considerable heterogeneity [63, 64]. We also conducted the meta-regression analyses using the type of PND, study year (categorical variable), income levels (low income, lower-middle income, upper-middle income), region, country, study design, and age months of outcome measured (continuous variable) with Sidik–Jonkman random-effects method for each association (RRs and ORs) between PND and outcomes (stunting and underweight). All analyses were performed using Stata Version 17 [65].

## Results

### Description of studies

**Fig 1:**
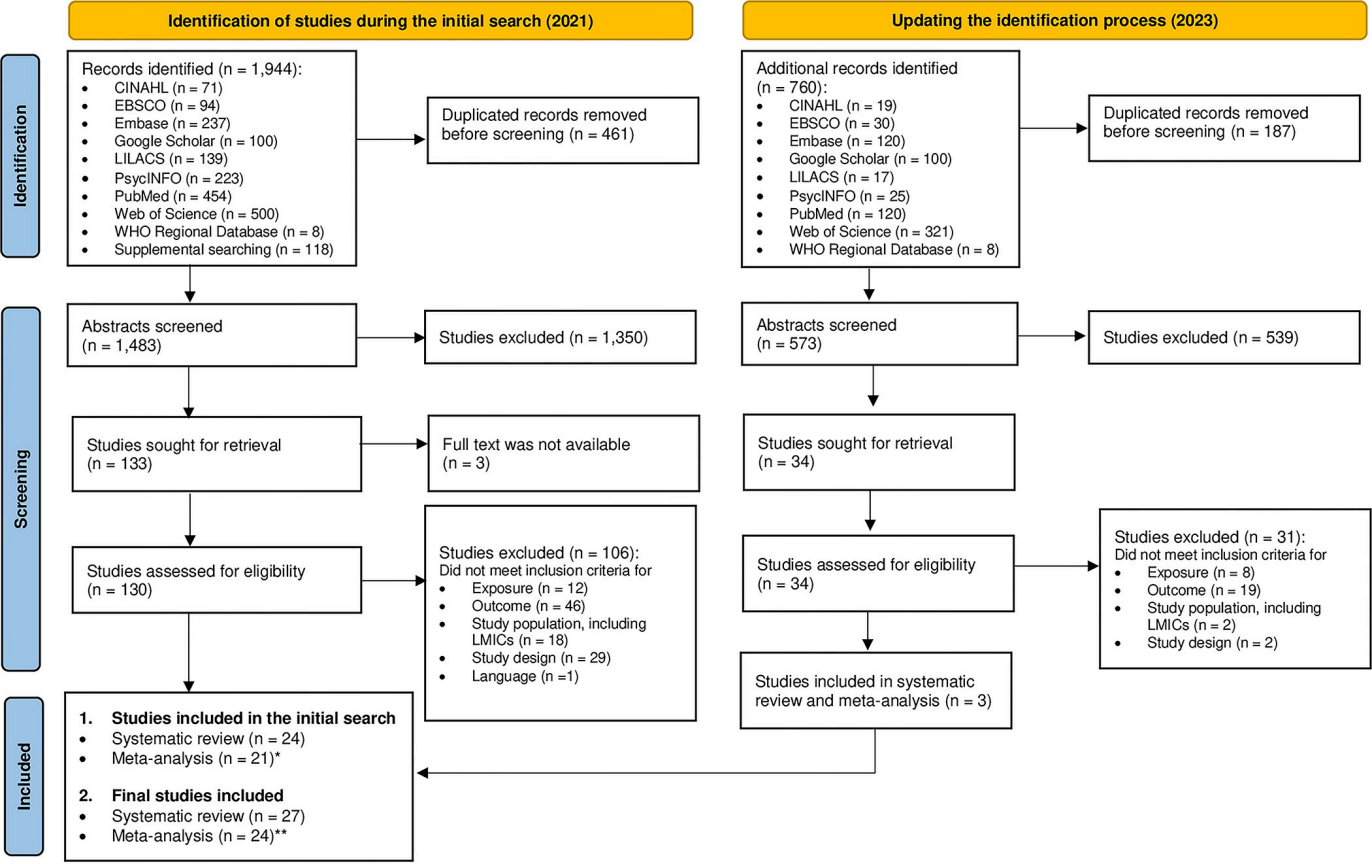
PRISMA flowchart. *From*: Page MJ, McKenzie JE, Bossuyt PM, Boutron I, Hoffmann TC, Mulrow CD, et al. The PRISMA 2020 statement: an updated guideline for reporting systematic reviews. BMJ 2021;372:n71. doi: 10.1136/bmj.n71. For more information, visit: http://www.prisma-statement.org/. Note: LMICs = low-income and middle-income countries. *A total of 24 sub-studies included since Bennett et al. (2016) conducted in 4 different countries and reported the findings in each country. *A total of 27 sub-studies included since Bennett et al. (2016) conducted in 4 different countries and reported the findings in each country.

During the initial database search in 2021, we identified 1,944 records and assessed titles and abstracts of 1,483 articles (Fig 1). From this set, we conducted the full-text review of 128 articles, except for four studies we could not locate (e.g. the conference abstract). In cases where specific estimates required for our research question were not reported (missing estimates for the research question), particularly when maternal depression was measured up to 5 years after birth, we contacted the authors to obtain the relevant estimate (n = 24). Among them, one group, Prado et al. (2019), provided the requested estimate (contacted person and date: Prado and September 19, 2021) [66]. Additionally, three studies, Surkan et al. (2008), Kaaya et al. (2016), and Wemakor and Iddrisu (2018), that measured PPD beyond the upper limit of our inclusion criteria (up to 24 months) were included in the systematic review and meta-analysis, and we excluded them in the sensitivity analysis.

Three studies, namely Nasreen et al. (2013), Brentani and Fink. (2016), and Christodoulou (2019), met the other inclusion criteria but did not report the variance of the estimate [34, 56, 67]. Consequently, these studies with missing variance of estimates were included in the systematic review but excluded from the meta-analysis. One study by Bennett et al. (2016), combined sub-studies conducted in India, Ethiopia, Peru, and Vietnam, was analyzed separately [58]. As a result, the systematic review encompassed 24 studies (including 27 sub-studies), while the meta-analysis included 21 studies (comprising 24 sub-studies) during the initial search [34, 35, 56, 58, 66–85].

During the search update in September 2023, we identified 760 studies and screened 573 studies based on their titles and abstracts (Fig 1). Among these, we conducted a full-text review of 34 studies, ultimately including three studies in the systematic review and meta-analysis [39, 86, 87]. As a result, across the two database search processes, a total of 26,261 mother-baby pairs from 27 studies (30 sub-studies) done in 15 countries were included in the final systematic review [34, 35, 39, 56, 58, 66–87] (Table 1). These 27 studies covered 24 studies on stunting, 19 studies on underweight, two studies on overweight/obestiy, and five studies on wasting. In the final meta-analysis, we pooled estimates from a total of 27 sub-studies (n of studies = 24) [35, 58, 66, 68–85]: 21 studies (24 sub-studies) were on stunting, 17 studies (20 sub-studies) on underweight, and three studies on wasting (S2 Data).

**Table 1:** Summary of the study characteristics included in the systematic review and meta-analysis for the association between perinatal depression and child adverse growth outcomes (stunting, underweight, overweight/obesity) in the low-income and middle- income countries (N = 26,261 from 27 studies).

First author (year)	Country	Study design	Sample size	Depression measure (cutoff)	Time of depression measure	Association measure	Time of outcome measure (after-birth)^a^	Stunting estimate (95% CI)	Underweight/overweight or obesity ^b^ estimate (95% CI)
Patel (2003)	India	Cohort	171	EPDS (≥11)	6 weeks postpartum	aOR	6 weeks	1.9 (0.5, 6.8)	3.2 (1.1, 9.4)
							6 months	3.2 (1.1, 8.9)	2.8 (1.1, 7.3)
Rahman (2004, 1)	Pakistan	Case- control	172	SRQ20 (≥ 10)	> 6 weeks postpartum	aOR	9 months		2.8 (1.2, 6.8)
Rahman (2004, 2)	Pakistan	Cohort	265	SCAN	3rd trimester	aOR	6 months	3.2 (1.1, 9.9)	3.5 (1.5, 8.6)
							12 months	2.8 (1.3, 6.1)	3.0 (1.5, 6)
Anoop (2004)	India	Case- control	144	SCID for DSM-III-R	< 6 weeks postpartum	aOR	6–12 months		7.4 (1.6, 38.5)
Tomlinson (2006)	South Africa	Cohort	147	SCID for DSM-IV	2 months postpartum	OR	18 months	2.52 (0.98, 6.47)	2.32 (0.90, 6.00)
Surkan (2008)	Brazil	Cross-sectional	595	CES-D (≥16)	6–24 months postpartum ^c^	aOR	6–24 months	1.8 (1.1, 2.9)	1.8 (0.6, 5.6)
Adewuya (2008)	Nigeria	Case- control	242	SCID for DSM-III-R	< 6 weeks postpartum	OR	6 weeks	1.55 (0.43, 5.65)	2.60 (0.87, 7.62)
							3 months	3.28 (1.03, 10.47)	3.19 (1.21, 8.40)
							6 months	3.34 (1.18, 9.55)	4.21 (1.36, 13.20)
							9 months	2.68 (0.82, 8.80)	2.84 (0.98, 8.24)
Avan (2010)	South Africa	Cohort	892	PDI (≥19)	> 6 weeks postpartum	aRR	24 months	1.61 (1.02, 2.56)	0.76 (0.39, 1.49)
Ndokera (2010)	Zambia	Cross- sectional	278	SRQ20 (≥8)	> 6 weeks postpartum	RR	2–12 months	1.48 (0.35, 6.22)	1.48 (0.35, 6.22)
Nasreen (2013)^h^	Bangladesh	Cohort	652	EPDS	Third trimester	Adjusted beta-coefficients	6–8 months	-0.26^i^ (-0.50, -0.01)	
					2–3 months postpartum			-0.26^i^ (-0.54, 0.01)	-0.38^i^ (-0.72, -0.05)
Brentani and Fink (2016)^h^	Brazil	Cross-sectional	798	EPDS (>13)	1 year	Adjusted beta-coefficients	1 year	0.02 (SE 0.03)	0.01 (SE 0.05)
Kaaya (2016)	Tanzania	Cohort	699	HSCL-25	During pregnancy	aRR	< 2 years	0.83 (0.67, 1.02)	0.86 (0.68, 1.09)
					<2 years postpartum^c^			0.86 (0.72, 1.03)	0.80 (0.54, 0.99)
					Cumulative postpartum			1.03 (1.01, 1.04)	1.02 (1.00, 1.04)
Madeghe (2016)	Kenya	Cross- sectional	200	EPDS (≥13)	6–16 weeks postpartum	aOR	6–16 weeks		5.79 (2.14, 15.62)
Upadhyay and Srivastava (2016)	India	Cohort	1,833	SRQ20 (≥8)	< 6 weeks postpartum	aOR	5–21 months	1.53 (1.21, 1.92)	
Bennett (2016)	India	Cohort	1,930	SRQ20 (≥8)	> 6 weeks postpartum	aRR	1 year	1.18 (1.03, 1.35)	1.11 (0.97, 1.26)
	India		1,930				5 years	1.07 (0.95, 1.22)	1.16 (1.00, 1.29)
	Peru		1,946				1 year	1.06 (0.93, 1.22)	0.85 (0.60, 1.19)
	Peru		1,946				5 years	0.96 (0.85, 1.09)	0.99 (0.67, 1.46)
	Vietnam		1,961				1 year	0.91 (0.81, 1.02)	1.29 (1.03, 1.62)
	Vietnam		1,961				5 years	1.12 (0.95, 1.33)	1.19 (0.95, 1.48)
	Ethiopia		1,885				1 year	0.91 (0.81, 1.02)	1.01 (0.89, 1.15)
	Ethiopia		1,885				5 years	0.94 (0.82, 1.09)	0.95 (0.80, 1.12)
Saeed (2017)	Pakistan	Cross- sectional	325	AKUADS (≥20)	12 months postpartum	aOR	< 2 years	3.15 (1.91, 5.18)	3.26 (1.99, 5.34)
Lima (2017)	Brazil	Cohort	1,124	CES-D (≥22)	2nd trimester	aOR	12–32 months	1.09 (0.62, 1.89)	0.84 (0.54, 1.31)
Wemakor and Iddrisu (2018)	Ghana	Cross-sectional	200	CES-D (≥20)	6–23 months postpartum ^c^	OR	6–23 months	1.05 (0.53, 1.73)	
Joshi and Raut (2019)	India	Cross- sectional	300	EPDS (≥10)	> 6 weeks postpartum	POR	< 1 year	1.49 (0.83, 2.65)	1.08 (0.59, 1.98)
Prado (2019)	Ghana	Cohort	1,003	EPDS^d^	6 months postpartum	OR^f^	18 months	0.73 (0.38, 1.39)	
Christodoulou (2019)^h^	South Africa	Cohort	470	EPDS (>13)	At birth	OR^g^	3 months	1.67	0.75
							6 months	0.50	0.00
							9 months	1.50	1.00
							12 months	1.13	1.00
							24 months	0.75	0.67
Ahmadi Gharaei (2020)	Iran	Cohort	470	DASS^e^	During pregnancy	aRR	6 months	1.10 (0.67, 1.81)	1.71 (1.07, 2.09)
Anato (2020)	Ethiopia	Cross- sectional	232	EPDS (≥13)	6 weeks postpartum	aOR	5–10 months	2.55 (1.24, 5.25)	
Tome (2020)	Zimbabwe	2ndary data analysis (cohort) of RCT	4,025	EPDS (≥12 or suicide)	During pregnancy	aOR	3 months	0.83 (0.53, 1.30)	
						aOR	6 months	0.82 (0.53, 1.28)	
						aOR	12 months	1.13 (0.84, 1.52)	
						aOR	18 months	1.10 (0.81, 1.49)	
Ricci (2023)	South Africa	2ndary data analysis (cross-sectional) of RCT	428	EPDS (>13)	6–9 months postpartum	aRR	6–9 months	1.02 (0.63, 1.66)	0.81 (0.41, 1.63)
Shriyan (2023)	India	Cohort	1,135	EPDS (>13)	During pregnancy and delivery within 48 h	aOR	1 year	1.72 (1.22, 2.43)	
Saleh (2023)	Tanzania	Cohort	1,739 for height 1,525 for weight	HSCL-8	During pregnancy	aRR	1 year	0.92 (0.83, 1.02)	0.80 (0.56, 1.16)

a: For the studies that measured the outcomes repeatedly, we used the measure of association that was reported close to year 1 to prevent the correlation of repeated measurements.

b: Studies except for Brentani and Fink (2016) and Lima et al. (2017) used underweight as an outcome.

c: In the case of PPD and cumulative PPD, the timepoint of maternal depression measure was up until 24 months, which is beyond our inclusion criteria for PND.

d: A locally validated adapted version was used.

e: Authors reported the association between depression score in the DASS and the outcome.

f: The research team constructed an odds ratio and 95% CI based on raw data provided by the original study authors.

h: Studies excluded in the meta-analysis due to heterogeneity of measurement and no availability for CIs or SE.

i: Figs 2 and 3 from Nasreen et al. (2013) showed the mean HAZ and WAZ were lower among mothers with APD compared to mothers without APD, meaning that the negative coefficient predicts the increased risk rather than the protective effect.

Note: AKUADS = Aga Khan University Anxiety and Depression Scale, APD = antepartum depression, aOR = adjusted odds ratio, CES-D = Center for Epidemiological Studies-Depression, DASS = Depression Anxiety Stress Scales, EPDS = Edinburgh Postnatal Depression Scale, HSCL-8/-25 = Hopkins Symptom Checklist-8/-25, PDI = Pitt Depression Inventory, POR = prevalence odds ratio, PND = perinatal depression, PPD = postpartum depression, RCT = randomized controlled trial, aRR = adjusted risk ratio, SCID for DSM-III-R, or -IV = Structured Clinical Interview for Diagnostic and Statistical Manual of Mental Disorders-III-R, or -IV, SCAN = Schedules for Clinical Assessment in Neuropsychiatry, SE = standard error, SRQ20 = Self Reporting Questionnaire-20

Among the total studies included in the systematic review (n = 27), fourteen (52%) were cohort studies, nine (33%) were cross-sectional, three (11%) were case-control studies, and 1 (4%) was a secondary data analysis (cohort study) within an RCT. Only seven studies (26%) utilized APD, including those that measured depression at birth or within two days after birth. All studies that reported the RR between APD and stunting or underweight were cohort studies. Eighteen studies (67%) measured depression as PPD, and two studies (7%) assessed both APD and PPD.

Among the studies included in the meta-analysis for the association between PND and stunting (n = 21), 14 (67%) reported ORs and 7 (33%) studies reported RRs; seven APD studies (33%), 12 PPD studies (57%), one study (5%) that examined APD and PPD separately, and one study (5%) that used PND as a combined exposure. In the case of the meta-analysis for the association between PND and underweight, which included 17 studies, 10 (59%) reported ORs, and 7 (41%) reported RRs. Among these, 3 studies focused on APD (18%), 13 on PPD (76%) studies, and one study (6%) examined APD and PPD separately. For the meta-analysis of the association between PND and wasting, which encompassed 3 studies, all reported RRs: 1 APD study (33%) and 2 PPD (67%) studies.

Various screening tools, cutoff scores, and clinical diagnostic measures were employed to identify mothers with PND. Among the utilized questionnaires, the Edinburgh Postnatal Depression Scale (n = 11) was the most common measure of depression, followed by the Self-Reporting Questionnaire-20 (n = 4), Center for Epidemiological Studies Depression scale (n = 3), Hopkins Symptom Checklist (n = 2), Aga Khan University Anxiety and Depression Scale (n = 1), Depression Anxiety Stress Scales (n = 1), and Pitt Depression Inventory (n = 1) (Table 1). In most cases, the authors described in-detail the reasons for the selected questionnaire, such as reliability and validity, and for the selected cutoff score, the process of the local adaptation, or the use of an interpreter due to literacy issues. The semi-structured or structured clinical interview, such as the Schedules for Clinical Assessment in Neuropsychiatry (n = 1), and Structured Clinical Interview for Diagnostic and Statistical Manual of Mental Disorders-III-R or -IV (n = 3), were also applied by the trained researchers or psychiatrists.

Among the total of 30 sub-studies included in the systematic review, six were conducted in India, four from South Africa, three each from Pakistan and Brazil, two from Ethiopia, Ghana, and Tanzania, and one each from Bangladesh, Vietnam, Iran, Kenya, Nigeria, Zambia, Zimbabwe, and Peru: Asia 11 studies, Middle East I study, Africa 14 studies, and America 4 studies. A descriptive summary of all studies included in the analysis and adjusted covariates in each study are shown in Tables 1 and 2, respectively.

**Table 2:** Adjusted covariates in each study included in the systematic review and meta-analysis.

Measure of Association	First author (year)	Adjusted variables
Odds ratio	Patel (2003)	Birthweight
	Rahman (2004, 1)	Birthweight, number of children, and socioeconomic status
	Rahman (2004, 2)	Low birthweight, breastfeeding, > 5 diarrheal episodes/year, parent education, maternal financial empowerment, relative poverty
	Anoop (2004)	Maternal intelligence, low birthweight, SES, duration of exclusive breastfeeding, duration of supplementary breastfeeding, immunization status, mother’s literacy
	Surkan (2008)	Child age, child gender, birthweight, breast-feeding duration, maternal education attainment, sanitation scale score, SES and living conditions scale score, number of children living in household, participation in Family Health Program, maternal self-efficacy score
	Madeghe (2016)	Maternal age, marital status, parity, income
	Upadhyay and Srivastava (2016)	Pregnancy intention, social supports, birth size, preterm birth, child sex, ever breastfed, serious illness, mother’s age at birth of child, maternal education, maternal working status, antenatal care, iron folic acid tablets, tetanus injection, household head’s education status, sex of household head, wealth index, drinking water, toilet facility, income socks, religion, caste, place of residence
	Saeed (2017)	Maternal age, education, income, occupation, child gender
	Lima (2017)	Monthly family income, maternal age, number of children, maternal schooling
	Anato (2020)	Wealth index, education, occupation, household food security, maternal workload
	Tome (2020)	Cluster, intervention arms, maternal at birth, mother’s height, mother’s mid upper arm circumference, child sex, single or multiple birth, household wealth index, presence of functioning hand washing station with water and soap, gender of household head, number of children under-fives, household dietary diversity, mother’s HIV status at enrolment
	Shriyan (2023)	Gestational age at delivery, age of the respondent, education, SES, parity, weight, height, gestational diabetes, birthweight, and smoking status of participants’ husband.
Risk ratio	Avan (2010)	SES index, maternal age, child gender, preterm delivery
	Kaaya (2016)	Maternal perceived low social support, age, marital status, education, financial dependence, anemia, weight, height, HIV stage at enrollment and counselling session attendance, child’s gender, birthweight, gestational age, breastfeeding, HIV status, vitamin A, multivitamin and other disease events.
	Bennett (2016)	Maternal schooling, sex of child, child age in months, wealth index
	Ahmadi Gharaei (2020)	Stunting: multivitamin intake, maternal education, birth height Underweight: infant sex, having tooth at 6 months, birthweight, infection contraction within 6 months
	Ricci (2023)	Maternal age
	Saleh (2023)	Maternal age, maternal education, household wealth quintile, marital status, maternal employment status, parity, maternal CD4 category, maternal WHO HIV disease stage, taking ART, trial site, child sex, child age, child twin status, and randomized regimen
Beta-coefficient	Nasreen (2013)	Household SES, infant’s sex, infant’s weight at 2–3 months, infants’ temperament (unadaptable)
	Brentani and Fink (2016)	Child age, child sex, twin, born small for gestational age, prematurity, maternal age, mother as a primary caregiver, caregiver primary education, caregiver secondary education, caregiver higher education, caregiver married, asset status

Note: ART = antiretroviral therapy, HIV = human immunodeficiency virus, SES = socioeconomic status

### Primary outcomes

#### Stunting

*Meta-analysis of ORs between PND and stunting*

Children aged 6 to 32 months whose mothers had PND had significantly higher odds of stunting, OR 1.63 (n = 14, 95% CI 1.32, 2.02, I^2^ = 56.0%), compared to those of mothers without PND (Fig 2). A sensitivity analysis that excluded studies with unadjusted ORs or PND measured up to 2 years produced a higher pooled estimate: OR 1.81 (n = 9, 95% CI 1.39, 2.36, I^2^ = 62.3%). Further, when we limited the estimate to the recent publication, the overall effect remained substantial at 1.64 (n = 6, 95% CI 1.22, 2.19, I^2^ = 69.4%).

**Fig 2:**
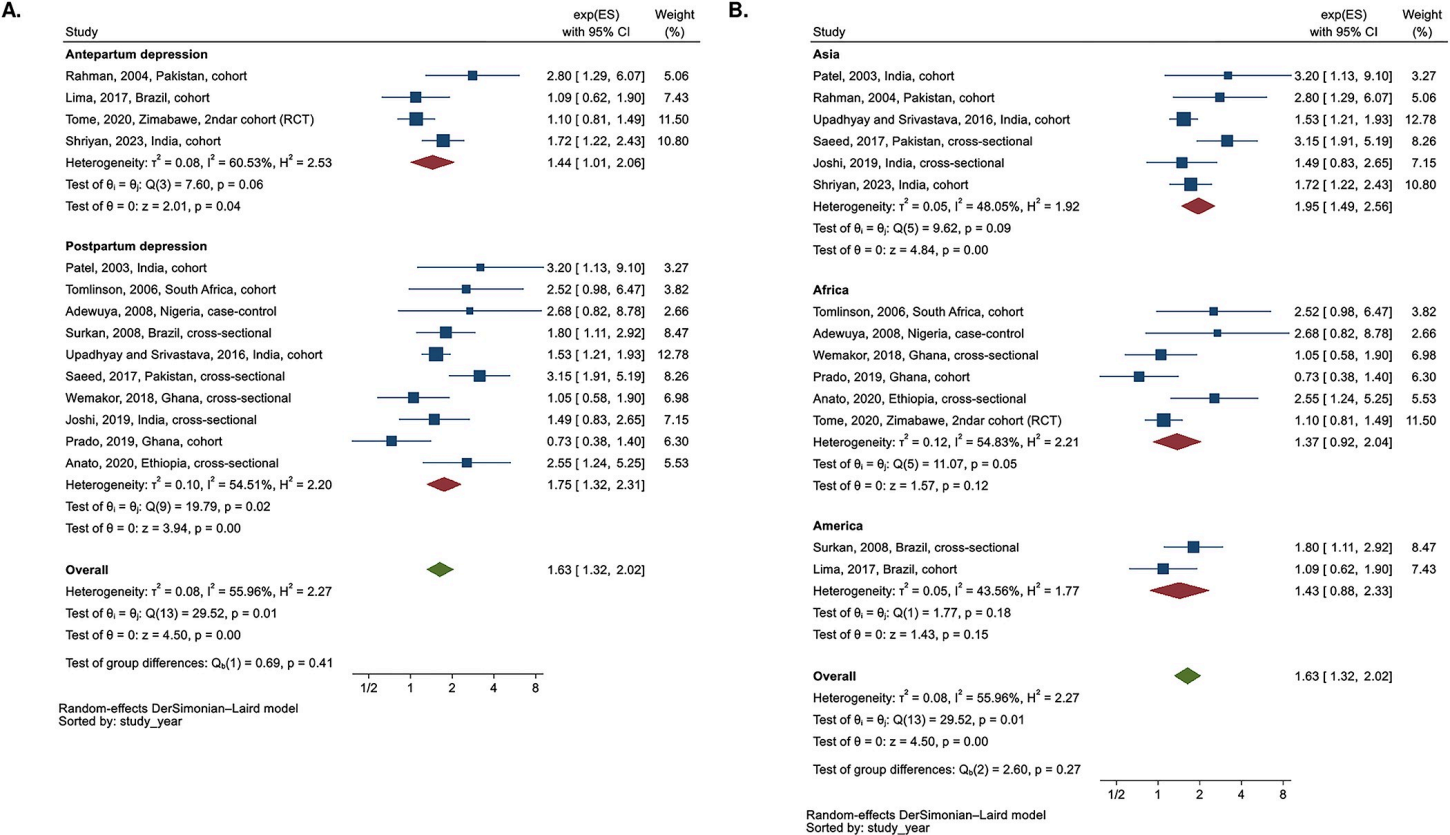
Meta-analysis of odds ratio between perinatal depression and stunting (n = 14)–subgroup analysis according to type of perinatal depression (antepartum vs. postpartum) and region. **A.** Subgroup analysis according to type of perinatal depression. **B.** Subgroup analysis according to region.

In a subgroup analysis based on the type of PND (Fig 2A), APD and PPD were associated with 1.44 times the odds of stunting (n = 4, 95% CI 1.01, 2.06, I^2^ = 60.5%) and 1.75 times the odds of stunting (n = 10, 95% CI 1.32, 2.31, I^2^ = 54.5%), respectively. In a subgroup analysis by region (Fig 2B), studies conducted in Asia showed the greatest overall association of OR 1.95 between PND and stunting (95% CI 1.49, 2.56, I^2^ = 49.1%) than in Africa or America.

When we stratified the estimates according to the outcome timepoint (S1 Fig), the effect of APD was strongest when measured around 1 year postpartum (S1A Fig). The effect of PPD was most pronounced when measured before 1 year, and for both, the effect on stunting at each time point decreased after one year (S1B Fig). In a subgroup analysis by study design (S2 Fig), cohort studies showed an attenuated effect of PPD, OR of 1.55, on stunting compared to other study designs (n = 4, 95% CI 0.92, 2.63, I^2^ = 62.4%).

Meta-analysis of RRs between PND and stunting

Children aged 6 to 24 months whose mothers had PND had similar risks of stunting, with an RR 0.99 (n of sub-studies = 10, 95% CI 0.90, 1.08, I^2^ = 56.0%), compared to children of mothers without PND. In the study by Kaaya et al. (2016), among the three estimates related to the timing of PND (APD, PPD, and cumulative PPD), we selected the one measured for APD that met the inclusion criteria of our study. Sensitivity analyses produced estimates that were similar to those pooled in the main analysis.

**Fig 3:**
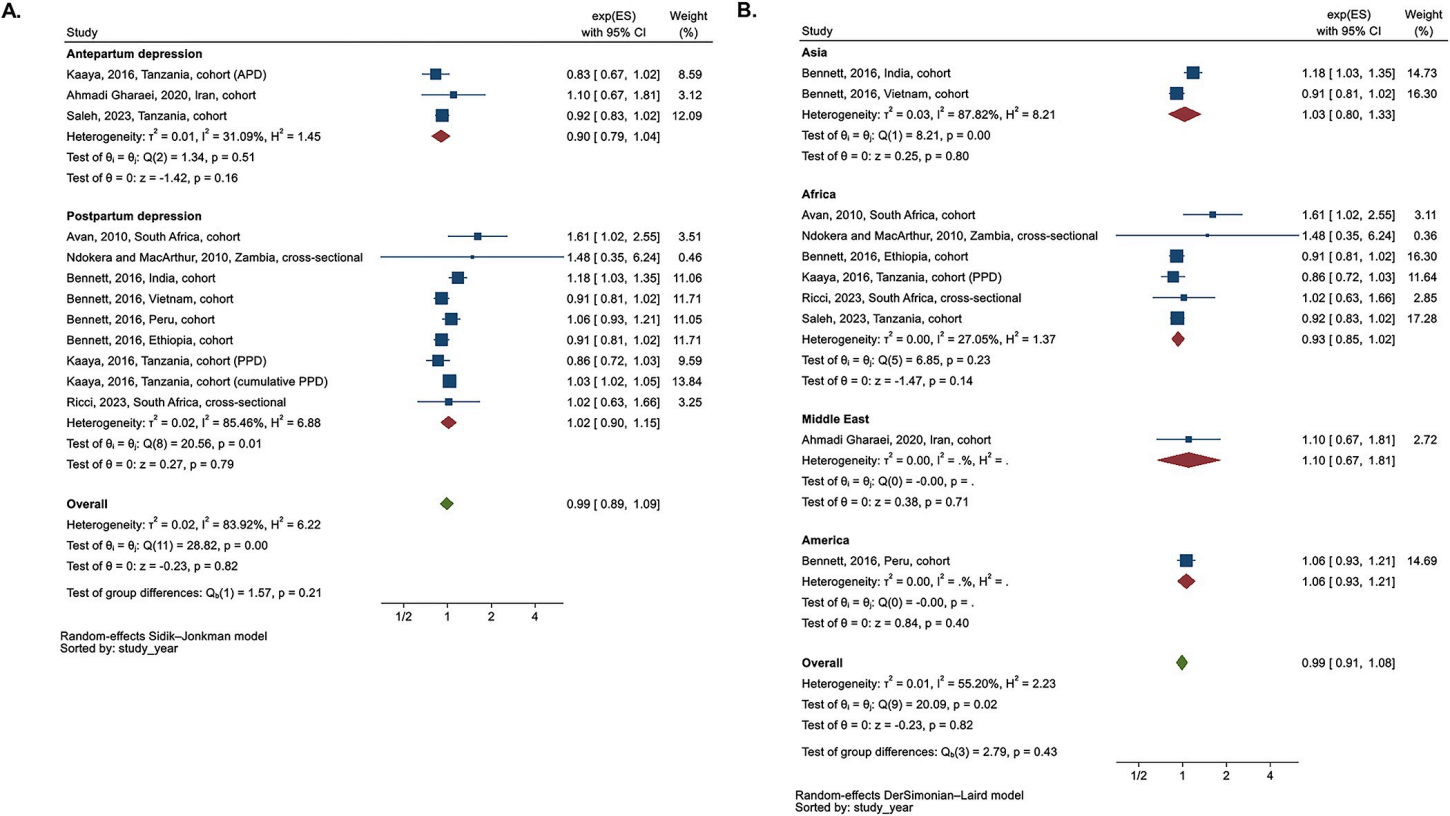
Meta-analysis of the risk ratio between perinatal depression and stunting of children. **A.** Subgroup analysis according to type of perinatal depression ^a^. **B.** Subgroup analysis according to region.

The subgroup analyses based on the type of PND, region, timepoint of outcome measurement, and study design (Fig 3, S3 and S4 Figs) consistently showed the null effects on stunting, while the overall effect of APD on stunting at each time point showed a decreasing trend as child ages (S4A Fig, n = 3).

Additional findings on the association between PND and stunting

Three additional studies were included in the systematic review. Nasreen et al. (2013), a cohort study in Bangladesh (Asia), reported that mothers with APD had statistically significantly higher risk of stunting among children aged 6–8 months than mothers without APD (“antepartum depressive symptoms predicted infant’s stunting”). Brentani and Fink (2016), a cross-sectional study in Brazil, observed a slight increase in the adjusted probabilities of stunting among one-year-old children from mothers with PPD compared to mothers without PPD; however, this difference was not statistically significant. In line with the subgroup analysis of OR based on the timing of stunting measurement, Christodoulou et al. (2019), a cohort study in South Africa, suggested that mothers with APD, measured at birth, had higher odds of stunting among 1-year-old children (OR 1.13) when compared to mothers without APD which was followed by protective odds of stunting among children aged two years (OR 0.75).

#### Underweight

*Meta-analysis of ORs between PND and underweight*

**Fig 4:**
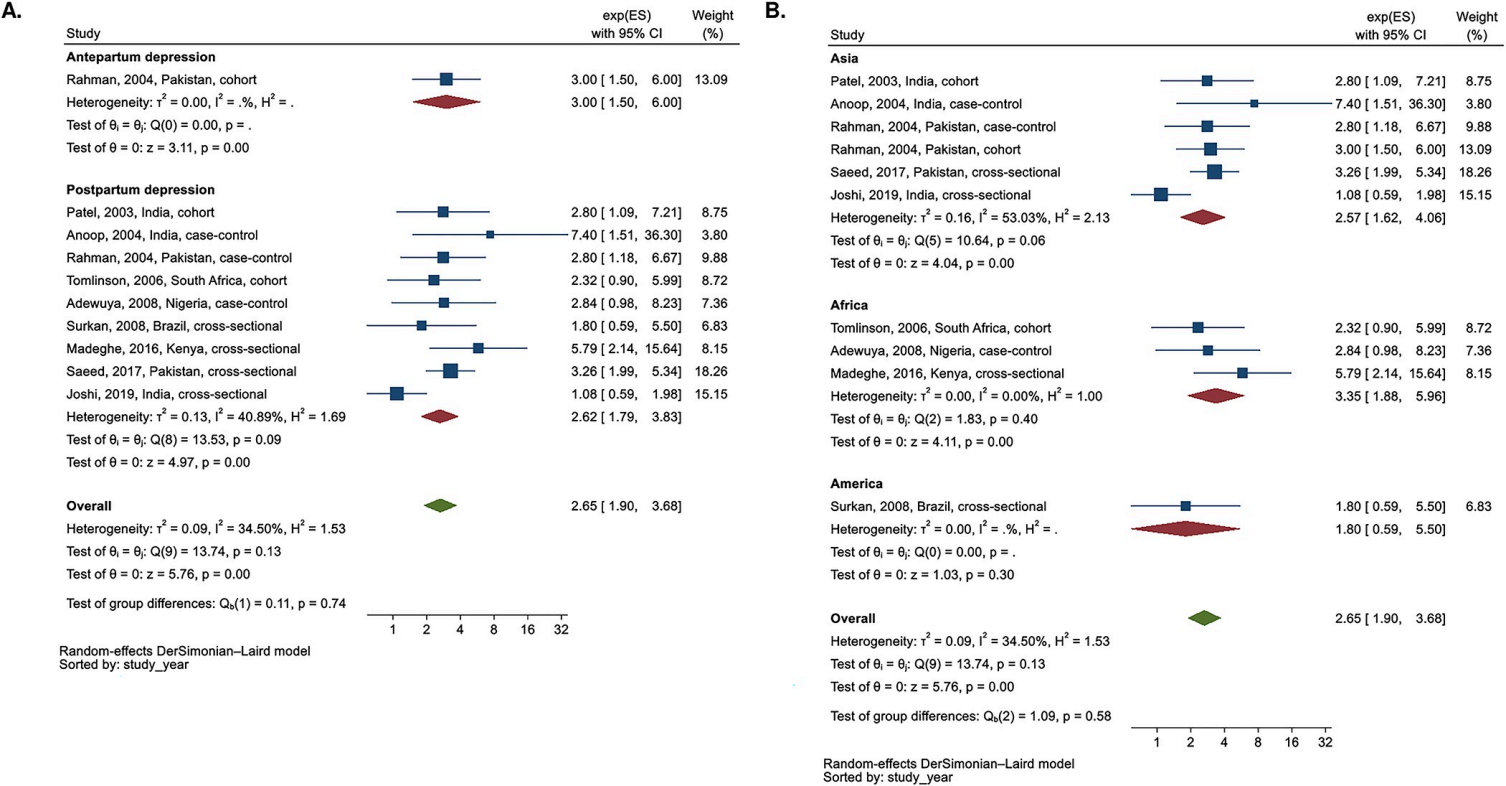
Meta-analysis of odds ratio between perinatal depression and underweight (n = 10)–subgroup analysis according to region and type of perinatal depression (antepartum vs. **postpartum). A.** Subgroup analysis according to type of perinatal depression. **B.** Subgroup analysis according to region.

Children aged 6 to 24 months whose mothers had PND exhibited significantly higher odds of underweight, with an OR of 2.65 (n = 10, 95% CI 1.90, 3.68, I^2^ = 34.5%), when compared to children of mothers without PND (Fig 4). A sensitivity analysis that 1) excluded studies with unadjusted ORs or PND measured up until 2 years and 2) recent publications yielded higher pooled estimates: 1) OR 3.25 (n = 7, 95% CI 2.41, 4.39, I^2^ = 0.0%) and 2) OR 3.67 (n = 2, 95% CI 2.33, 5.79, I^2^ = 2.9%).

The subgroup analysis comparing underweight and APD versus PPD showed a similar trend (Fig 4). In the subgroup analysis based on region (Fig 4B), although the number of included studies was small, the magnitude of the overall OR between PND and underweight followed the order of Africa (n = 3) > Asia (n = 6) > America (n = 1).

When we stratified the estimates according to the timepoint of outcome (S5 Fig), the overall association between PPD and underweight was the highest at the earliest time point (4 months) (n = 1), followed by the decreasing patterns. We presented the subgroup analysis according to the study design in the S6 Fig.

Meta-analysis of RRs between PND and underweight

Children aged 6 to 24 months whose mothers had PND had a similar risk of underweight, with a RR of 1.06 (n of sub-studies = 10 (in Kaaya et al. (2016), we selected APD estimate), 95% CI 0.93, 1.22, I^2^ = 49.5%), compared to those of mothers without PND. A sensitivity analysis, which excluded studies with unadjusted RRs or PND measured up to 2 years and limited to the recent publications, produced almost the same pooled estimates as the main analysis.

**Fig 5:**
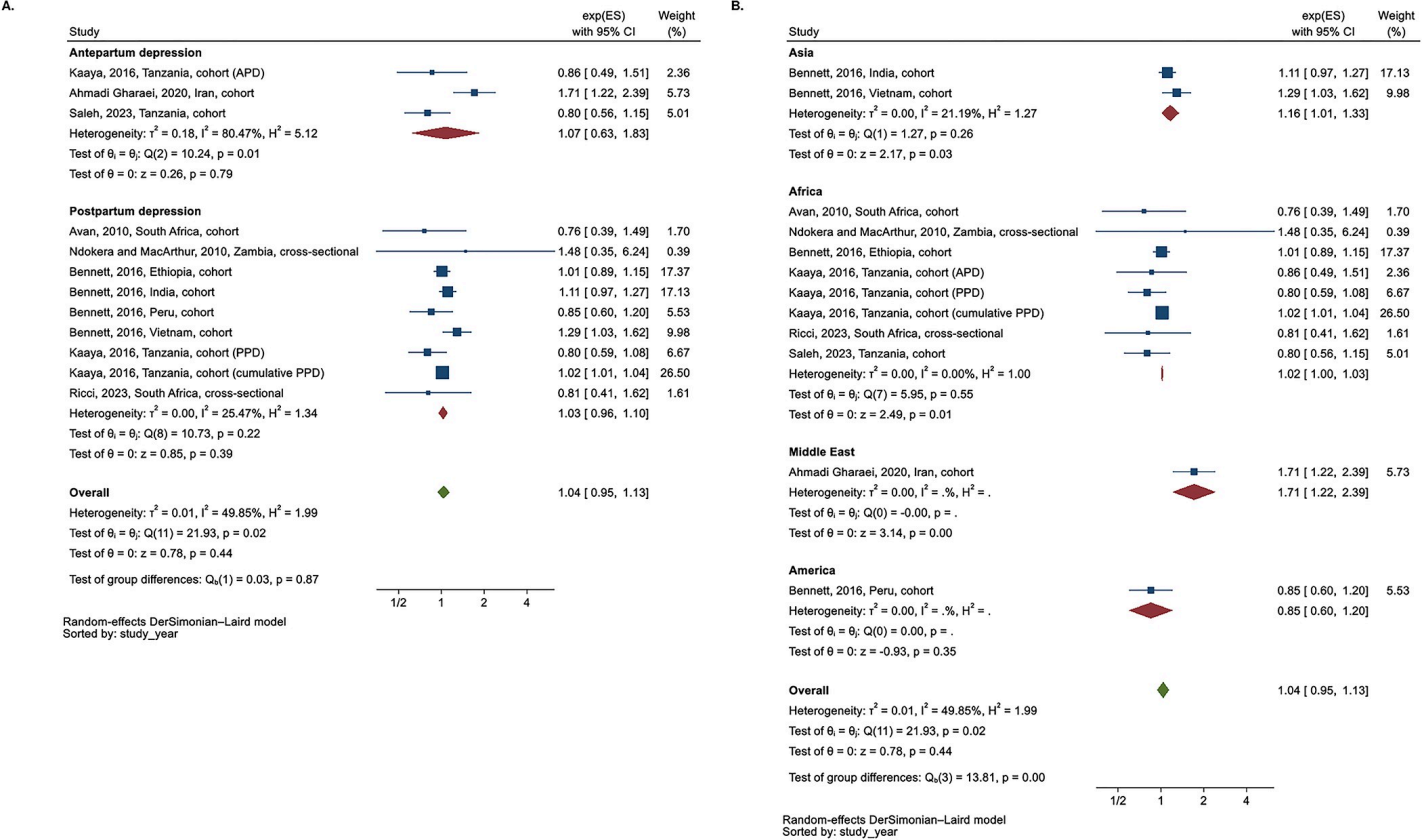
Meta-analysis of the risk ratio between perinatal depression and underweight of children. **A.** Subgroup analysis according to type of perinatal depression ^a^. **B.** Subgroup analysis according to region.

The subgroup analysis comparing underweight and APD versus PPD yielded results that closely resembled the overall analysis (Fig 5A). In the subgroup analysis based on the region (Fig 5B), the effect direction varied by region. In America, one study indicated a potentially protective effect on child underweight among mothers with PND compared to those without PND, though this finding was not statistically significant. Conversely, the overall effect in Asia (RR 1.16 (95% CI 1.01, 1.33)), Africa (RR 1.02 (95% CI 1.00, 1.03)) and one study from the Middle East suggested a higher risk of child underweight among mothers with PND compared to their counterparts.

We presented the subgroup analysis stratified based on the exposure and outcome timepoint and study design in S7 and S8 Figs.

Additional findings on the association between PND and underweight

Three additional studies were included in the systematic review. Nasreen et al. (2013) reported statistically significantly higher risk of underweight among children aged 6–8 months (“Maternal postpartum depressive symptoms independently predicted infant’s underweight”) (67). Christodoulou et al. (2019) presented percent proportions suggesting a null or protective effect of APD measured at birth on underweight over 2 years following birth.

#### Wasting

Five studies that met the inclusion criteria reported wasting with heterogeneous measures of association (34,75,77,83,87) (Table 3). Joshi and Raut (2019) in India reported an almost null prevalence OR for wasting among infants aged less than one year when comparing mothers with and without PPD (prevalence OR = 1.01, 95% CI 0.51, 2.01). In South Africa, Christodoulou showed variations in wasting among mothers with and without APD over two years.

**Table 3:** Summary of the study characteristics included in the systematic review and meta-analysis for the association between perinatal depression and wasting of children in low-income and middle-income countries.

First author (year)	Country	Study Design	Sample size	Depression measure (cutoff)	Time of depression measure	Association measure	Time of outcome measure (after-birth)	Wasting (95% CI)
Avan (2010)	South Africa	Cohort	892	PDI (≥19)	> 6 weeks postpartum	aRR	24 months	0.66 (0.30, 1.42)
Kaaya (2016)	Tanzania	Cohort	699	HSCL-25	During pregnancy	aRR	< 2 years	1.06 (0.74, 1.51)
					<2 years postpartum^a^			0.59 (0.35, 1.01).
					Cumulative postpartum			1.08 (1.04, 1.12)
Joshi and Raut (2019)	India	Cross- Sectional	300	EPDS (≥10)	> 6 weeks postpartum	POR	< 1 year	1.01 (0.51, 2.01)
Christodoulou (2019)^c^	South Africa	Cohort	470	EPDS (>13)	At birth	OR^b^	3 months	1.45
							6 months	1.50
							9 months	0.00
							12 months	0.50
							24 months	2.00
Saleh (2023)	Tanzania	Cohort	1,739 for height 1,525 for weight	HSCL-8	During pregnancy	aRR	1 year	2.61 (1.03, 6.65)

a: In case of PPD and cumulative PPD, the timepoint of maternal depression measure was until 24 months, which is beyond of our inclusion criteria for PND.

b: The authors reported the risk of each stratum of exposure and outcome, and the research team calculated ORs. Since the CIs were not presented, these estimates are only included in the systematic review.

c: Studies excluded in the meta-analysis due to heterogeneity of measurement and no availability for CIs or SE.

Note: CIs = confidence intervals, OR = odds ratio, EPDS = Edinburgh Postnatal Depression Scale, HSCL-25 = Hopkins Symptom Checklist, PDI = Pitt Depression Inventory, POR = prevalence odds ratio, aRR = adjusted risk ratio, SE = standard error

**Fig 6:**
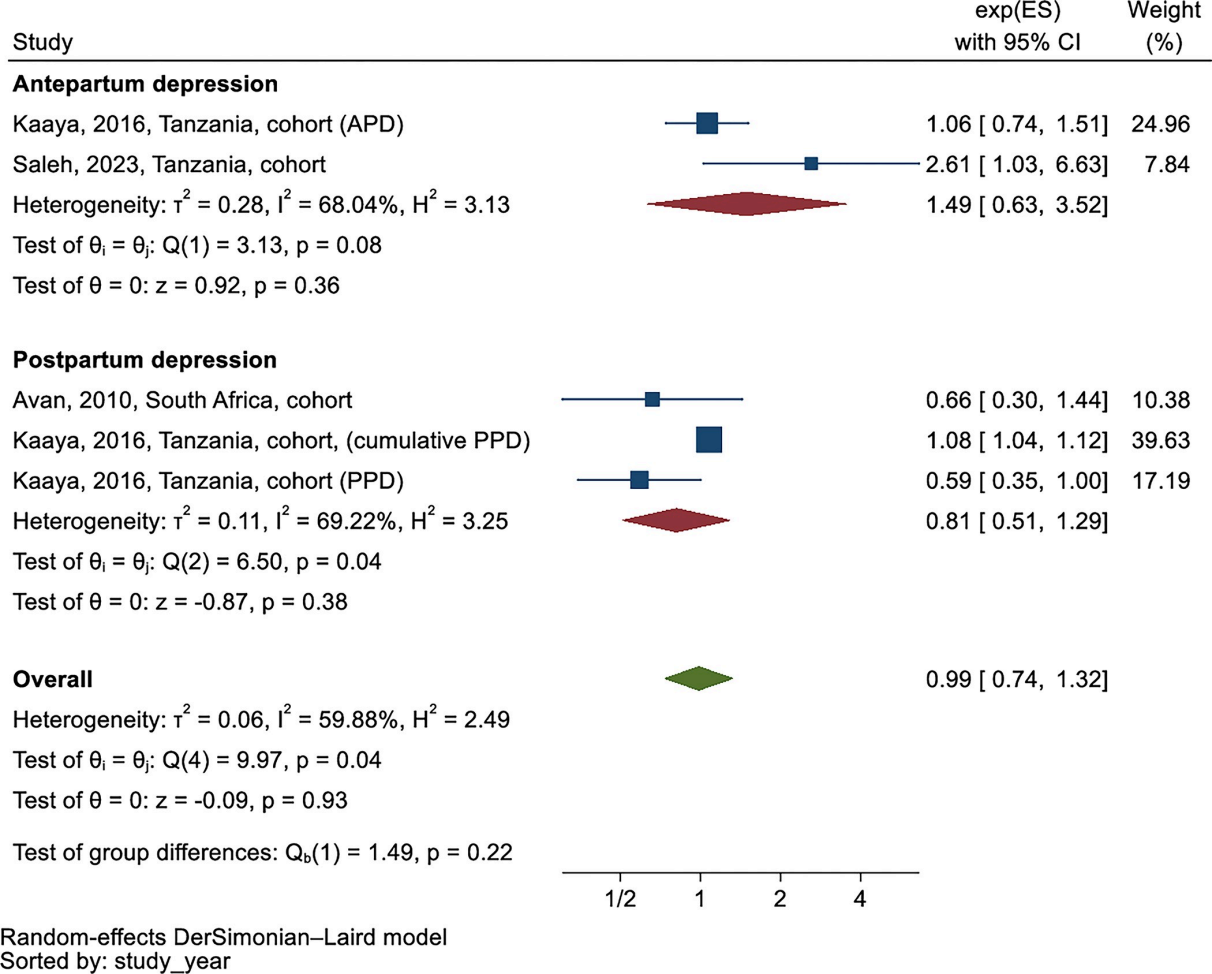
Subgroup analysis of the risk ratio between perinatal depression and wasting according to type of perinatal depression ^a^.

In Kaaya et al., only cumulative PND showed a statistically significant association with wasting among mothers with HIV (aRR 1.08 (95% CI 1.04, 1.12)) among three exposures of APD, PPD, and cumulative PPD. A meta-analysis of RRs from Avan et al. (2010), Kaaya et al. (2016), and Saleh et al. (2023) suggested contrasting effects of APD and PPD on the risk of wasting. The RR of APD on wasting was 1.49 (95% CI 0.63, 3.52, I^2^ = 68.0%), while the RR of PPD on wasting was 0.81 (95% CI 0.51, 1.29, I^2^ = 69.2%) (Fig 6).

#### Overweight/obesity

Only two studies reported overweight or obesity as an outcome. Lima et al. (2017), a cohort study in Brazil, found that mothers with and without APD had an OR of 0.84 for overweight among children aged 12 to 32 months (95% CI 0.54, 1.31), suggesting a potential protective effect of APD on overweight. On the contrary, Brentani and Fink (2016), a cross-sectional study in Brazil, reported a slight increase in the adjusted probabilities of obesity among children aged 1 year among mothers who had PPD compared to those without PPD, potential risk of PPD on obesity [56]. However, both were not statistically significant.

### Meta-regression

In the meta-regression analyses stratified for association type (RRs and ORs) and outcome types (stunting and underweight) (Table 4), the country explained the largest proportion of between-study variance (R^2^ = 74.7%) when examining the OR between PND and stunting. The region of Africa (p-value = 0.046) and the secondary analysis of RCT (p-value = 0.010) were found to be statistically significant predictors for the OR between PND and stunting. For the OR between PND and underweight, the study year explained the highest proportions of the between-study variance (R^2^ = 56.7%, p-value for 2019 = 0.058). For the RR between PND and stunting, the study year emerged as a significant predictor (R^2^ = 34.9%, p-value for the year 2010 = 0.039), and for the RR between PND and underweight, 1) country, 2) region, and 3) study year played significant roles: 1) R^2^ of countries = 79.5%, p-value for Iran = 0.003, p-value for Vietnam = 0.025, 2) R^2^ of region = 49.8%, p-value for Africa = 0.011, and 3) R^2^ of year = 42.9%, p-value for year 2020 = 0.008).

**Table 4:** Table of estimates from the meta-regression.

Association	Variable	Test of residual homogeneity		I^2^ (%)	R^2^ (%)
		Q residual	p-value		
Log OR between PND and stunting	APD vs. PPD	40.65	0.004	66.8	11.2
	Study year	21.15	0.048	60.2	24.6
	Income level	49.27	0.000	71.4	0.0
	Region	35.46	0.012	66.4	10.9
	Country	8.05	0.887	37.9	74.7
	Study design	26.53	0.088	60.8	31.5
	Age months of outcome measured	50.52	0.000	71.5	0.0
Log OR between PND and underweight	APD vs. PPD	14.63	0.331	31.5	0.0
	Study year	2.43	0.965	12.8	56.7
	Income level	14.38	0.347	31.0	0.7
	Region	13.54	0.331	32.2	0.0
	Country	8.25	0.509	31.17	0.0
	Study design	13.38	0.342	30.6	0.0
	Age months of outcome measured	14.84	0.318	30.3	0.0
Log RR between PND and stunting	APD vs. PPD	25.95	0.026	78.6	3.7
	Study year	24.76	0.016	74.1	34.9
	Income level	27.57	0.010	78.1	0.0
	Region	32.16	0.001	78.7	0.0
	Country	20.30	0.009	73.2	20.1
	Study design	32.79	0.003	82.4	0.0
	Age months of outcome measured	32.63	0.003	81.5	0.0
Log RR between PND and underweight	APD vs. PPD	29.43	0.009	83.7	0.0
	Study year	18.79	0.093	76.1	42.9
	Income level	28.15	0.009	79.5	5.8
	Region	8.27	0.764	70.7	49.8
	Country	5.71	0.680	34.6	79.5
	Study design	30.20	0.007	84.2	0.0
	Age months of outcome measured	28.82	0.011	81.7	0.0

Note. APD = antepartum depression, OR = odds ratio, PND = perinatal depression, PPD = postpartum depression, RR = risk ratio

### Publication bias

According to the measure of association and outcome strata, we obtained p-values in the Egger’s tests: OR of stunting (n = 14, p = 0.241), RR of stunting (n of sub-studies = 10, p = 0.292), OR of underweight (n = 10, p = 0.403), and RR of underweight (n of sub-studies = 10, p = 0.685). However, visual inspection of the funnel plot reveals substantial asymmetries in the pooled associations found in this study, suggesting the presence of publication bias due to missing studies (S9 Fig).

## Discussion

This systematic review and meta-analysis included 27 studies published from 2003 to 2023 in LMICs. We could not conduct a meta-analysis for obesity/overweight due to the limited numbers of relevant studies, and the association between PND and the rest of adverse growth outcomes exhibited variations based on the measure of association, country/region, PND types, and study design.

Among the studies reporting ORs, we observed that exposure to PND elevated the odds of stunting throughout the initial 32 months of a child’s life by 63%, while the influence of PPD alone was even more pronounced, increasing the odds of stunting by 75%. Stunting is unique in its association with fetal development during pregnancy, setting it apart from other forms of undernutrition. This condition, intertwined with both the woman’s nutritional status during pregnancy and the baby’s nutritional status after birth, can be primarily prevented through optimal maternal health and child feeding and care practices during the first 1,000 days [8]. PND exhibited an even more pronounced effect on children being underweight, with an OR of 2.73 among the studies reported ORs. Being underweight often indicates insufficient nutrient intake and can result from many environmental and genetic factors.

For both stunting and underweight, studies in India and Pakistan consistently reported high associations with depression [68, 70, 71, 80, 88]. However, studies from Iran and Vietnam also found high associations of PND for the risk of underweight and PND but not for the risk of stunting [58, 84]. While the pooled RR analyses of stunting and underweight differed from the pooled OR analyses, meta-regression showed factors like region, country, and study year explained these results. India and Pakistan have historically had the highest rates of stunting (more than 30%) among children for decades, and women in these regions often experience excessive workloads and high stress levels, particularly during perinatal periods [15, 89]. In line with this, Holm-Larsen et al. (2019), a cohort study in Tanzania, where the prevalence of stunting was high (35% during the study period) (S1 Table) [15], reported that the mean HAZ of 2 to 3-year-old children whose mothers had PPD was significantly lower by 0.32 units (95% CI -0.49, -0.15) compared to children whose mothers did not have PPD [28]. However, in Brazil, where the prevalence of stunting was much lower at 3.6%, Santos et al. (2010) reported no association between maternal PPD symptoms and stunting among 4-year-old children (S1 Table) [44].

Additionally, we could not obtain a consistent association between wasting and PND. Wasting, which heightens the risk of death in young children due to recent, severe weight loss, is a relatively rare outcome with a global prevalence of 6.7% compared to stunting at 22% among children under the age of five [15, 90]. The two studies meeting our criteria resulted in inconclusive associations, even with the qualitative analysis [75, 83]. Results from the studies excluded due to the heterogeneity of measures of association also presented conflicting findings. For example, in a cohort study in Tanzania (N = 1,128), the adjusted mean z-score of weight for height was significantly 0.21 units higher (95% CI 0.02, 0.40) among 2 to 3-year-old children with mothers with PPD compared to children without PPD, suggesting that PPD may be protective against wasting [28]. A Brazilian cohort study (N = 3,792), which we excluded due to the timing of outcome measurement, reported heterogeneous association according to PPD duration (12, 24, and 48 months) [44]. Given that wasting is an acute outcome of malnutrition, wasting may be more influenced by acute food crises, such as natural and man-made disasters, rather than maternal depression-related dysfunction. Additionally, in some LMICs, where the traditional extended family system remains prevalent, the risk of children experiencing wasting might be mitigated by the support of extended family members [89].

In light of the growing worldwide obesity epidemic, there is emerging evidence that PND may be associated with childhood overweight and obesity, although research in LMICs remains limited [91–93]. The mechanisms of child overweight and obesity are not fully understood but are believed to include a complex interplay of genetic, lifestyle, and environmental factors [94]. In LMICs, which face the challenge of a dual burden of malnutrition, overweight was initially associated with higher SES segments of society, while underweight was more common among the poor. However, as energy-dense, nutrient-poor diets become increasingly prevalent among lower SES groups, the prevalence of overweight has also risen [95]. We observed that only one study on overweight/obesity met the criteria for analysis, making it difficult to discern clear patterns. In addition to Lima et al. (2017), Santos et al. (2010) reported no association between PPD and overweight among 4-year-old children whose mothers had PPD symptoms once or twice over during the measurement period [44]. However, when mothers experienced PPD consistently, four-year-old children whose mothers had PPD had 1.6 times the adjusted odds of being overweight (95% CI 1.0, 2.5) compared to children who had mothers without PPD. Consequently, further research is needed to investigate the relationship between PND and overweight/obesity among children in LMICs.

Most meta-analyses demonstrated substantial heterogeneity. We observed significant variations in the characteristics of existing studies, including different measures and their thresholds, types of measure of association, features of the study population (country, region, time), and analytic approaches (selection of confounding factors) (Table 2). The included studies used different tools to measure PND, with different sensitivities and specificities, and this can be one plausible explanation for the observed heterogeneity. Moreover, more than half of the studies incorporated birthweight or preterm birth as adjusting covariates (Table 2). Birthweight or preterm birth, which could be on the causal pathways of PND and outcomes, may inadvertently block one of the causal pathways when we adjust for these factors during the analysis (S10 Fig). Lastly, it is essential to consider that there are often variable lags between depression onset and its clinical diagnosis. A postpartum diagnosis, for instance, does not conclusively rule out the presence of depression during or prior to pregnancy. Untreated APD is a strong predictor of PPD [96]. Roughly one-third of PPD cases have onset during pregnancy, and 27% of PPD started before pregnancy [97]. Therefore, conducting further longitudinal cohort studies that can account for these time-varying characteristics of exposure, outcome, and confounding factors is imperative.

Regardless, among current studies, there is a notable gap in the existing research pertains to assessing the degree of support system, family structure, the extent of a mother’s influence on child growth, and food insecurity. The unique characteristics and dynamics of LMICs, shaped by their demographic transitions, imply that the role of the community and support systems in childcare can vary significantly. Children whose mothers experience PND and the challenges of caregiving might have gained more support from the community, like extended families, to compensate for the insufficient support from mothers. Therefore, the context of social support and the meaning of food and economic insecurity could vary according to diverse LMICs, and future studies need to integrate more diverse socio-cultural contexts when investigating this research question [98].

## Conclusion

Limited studies investigated the association between PND and adverse child growth outcomes in LMICs. Nonetheless, mental health is underprioritized and underfunded, especially in most LMICs. Up to 85% of people with severe mental disorders in LMICs receive no treatment, while average per capita spending on mental health is just US $0.25 [99]. These underscore the long-term implications for child health and development and emphasize the disproportionate impact on children in resource-constrained settings. Despite the availability of evidence for supporting low-resource intervention programs, such as the WHO-recommended Thinking Healthy Program in LMICs, there remains a substantial gap in their implementation and scale-up [100]. As eloquently articulated by Howard et al. (2014), ‘There is no health without perinatal mental health’ [101]. Public health practitioners must prioritize the prevention, screening, and treatment of both prenatal and postpartum depression as integral components of efforts to combat child malnutrition in LMICs within the framework of broader maternal and child health programs.

## Supporting information

S1 Text The structured search strategy utilized. (DOCX)

S1 Table Summary of the findings on the association between maternal depression and child growth outcome. (XLSX)

S2 Table Quality assessment of studies with the Newcastle Ottawa Scale. (XLSX)

S3 Table Pooled associations measured using the Knapp–Hartung (KH) standard-error adjustments. (XLSX)

S1 Data Screened studies. (XLSX)

S2 Data Data for meta-analysis. (CSV)

S1 Fig Subgroup analysis of the odds ratio between perinatal depression and stunting according to the time point of stunting measurement. **A.** The odds ratio between antepartum depression and stunting according to the timepoint of outcome measurement. **B.** The odds ratio between postpartum depression and stunting according to the timepoint of outcome measurement.
(TIF)

S2 Fig Subgroup analysis of the odds ratio between perinatal depression and stunting according to study design. **A.** The odds ratio between antepartum depression and stunting according to the study design (n = 4). **B.** The odds ratio between postpartum depression and stunting (n = 10).
(TIF)

S3 Fig Subgroup analysis of the risk ratio between postpartum depression and stunting according to study design (n of sub-studies = 8). (TIF)

S4 Fig Subgroup analysis of the risk ratio between perinatal depression and stunting according to the time point of stunting measurement. **A.** The risk ratio between antepartum depression and stunting timepoint of outcome measurement. **B.** The risk ratio between postpartum depression and stunting according to the timepoint of outcome measurement.
(TIF)

S5 Fig Subgroup analysis of the odds ratio between postpartum depression and underweight according to underweight timepoint (n = 9). (TIF)

S6 Fig Subgroup analysis of the odds ratio between postpartum depression and underweight according to study design (n = 9). (TIF)

S7 Fig Subgroup analysis of the risk ratio between perinatal depression and underweight according to outcome timepoint. **A.** The risk ratio between antepartum depression and underweight according to outcome timepoint. **B.** The risk ratio between postpartum depression and underweight according to outcome timepoint.
(TIF)

S8 Fig Subgroup analysis of the risk ratio between postpartum depression and underweight according to study design (n of sub-studies = 8). (TIF)

S9 Fig Contour-enhanced Funnel plots of included studies in the meta-analysis. **A.** A funnel plot of studies reporting odds ratios between perinatal depression and stunting. **B.** A funnel plot of studies reporting odds ratios between perinatal depression and underweight. **C.** A funnel plot of studies reporting risk ratios between perinatal depression and stunting. **D.** A funnel plot of studies reporting risk ratios between perinatal depression and underweight.
(TIF)

S10 Fig Directed acyclic graph of time-varying characteristics of exposure, mediator-confounders, and outcomes. Note: APD = antepartum depression, PPD = postpartum depression, L0 = confounder before pregnancy, L1 = time-varying mediator-confounder after delivery (ie, family support). Time-varying exposure: APD and PPD. Time-varying outcome: birth weight and child growth outcome at year 1. Green arrows indicate the causal pathways we want to measure. Red arrows indicate the backdoor path that induces bias when we measure the association between exposure and outcome.
(TIF)

## References

[1] Woody CA, Ferrari AJ, Siskind DJ, Whiteford HA, Harris MG. A systematic review and meta-regression of the prevalence and incidence of perinatal depression. J Affect Disord. 2017 Sep 1;219:86–92. doi: 10.1016/j.jad.2017.05.003 28531848

[2] Shidhaye P, Giri P. Maternal depression: a hidden burden in developing countries. Ann Med Health Sci Res. 2014 Jul;4(4):463–5. doi: 10.4103/2141-9248.139268 25221688 PMC4160664

[3] World Health Organization, Fund UNP. Mental health aspects of women’s reproductive health: a global review of the literature [Internet]. World Health Organization; 2009 [cited 2021 Nov 2]. Available from: https://apps.who.int/iris/handle/10665/43846

[4] Stewart DE, Vigod S. Postpartum Depression. N Engl J Med. 2016;375(22):2177–86. doi: 10.1056/NEJMcp1607649 27959754

[5] The National Institute of Mental Health. National Institute of Mental Health. [cited 2021 Nov 2]. Perinatal Depression. Available from: https://www.nimh.nih.gov/health/publications/perinatal-depression

[6] Parsons CE, Young KS, Rochat TJ, Kringelbach MortenL, Stein A. Postnatal depression and its effects on child development: a review of evidence from low- and middle-income countries. Br Med Bull. 2012 Mar 1;101(1):57–79. doi: 10.1093/bmb/ldr047 22130907

[7] Gelaye B, Rondon MB, Araya R, Williams MA. Epidemiology of maternal depression, risk factors, and child outcomes in low-income and middle-income countries. Lancet Psychiatry. 2016 Oct;3(10):973–82. doi: 10.1016/S2215-0366(16)30284-X 27650773 PMC5155709

[8] Scott JA. The first 1000 days: A critical period of nutritional opportunity and vulnerability. Nutr Diet J Dietit Assoc Aust. 2020 Jul;77(3):295–7. 10.1111/1747-0080.12617 32478460

[9] Patel V, Saxena S, Lund C, Thornicroft G, Baingana F, Bolton P, et al. The Lancet Commission on global mental health and sustainable development. Lancet Br Ed. 2018;392(10157):1553–98. doi: 10.1016/S0140-6736(18)31612-X 30314863

[10] Dadi AF, Akalu TY, Wolde HF, Baraki AG. Effect of perinatal depression on birth and infant health outcomes: a systematic review and meta-analysis of observational studies from Africa. Arch Public Health. 2022 Jan 20;80(1):34. doi: 10.1186/s13690-022-00792-8 35057865 PMC8772173

[11] Kwon EJ, Kim YJ. What is fetal programming?: a lifetime health is under the control of in utero health. Obstet Gynecol Sci. 2017 Nov;60(6):506–19. doi: 10.5468/ogs.2017.60.6.506 29184858 PMC5694724

[12] Burger M, Hoosain M, Einspieler C, Unger M, Niehaus D. Maternal perinatal mental health and infant and toddler neurodevelopment—Evidence from low and middle-income countries. A systematic review. J Affect Disord. 2020 May 1;268:158–72. doi: 10.1016/j.jad.2020.03.023 32174474

[13] Liu Y, Kaaya S, Chai J, McCoy DC, Surkan PJ, Black MM, et al. Maternal depressive symptoms and early childhood cognitive development: a meta-analysis. Psychol Med. 2017 Mar;47(4):680–9. doi: 10.1017/S003329171600283X 27834159

[14] Black RE, Victora CG, Walker SP, Bhutta ZA, Christian P, de Onis M, et al. Maternal and child undernutrition and overweight in low-income and middle-income countries. Lancet Lond Engl. 2013 Aug 3;382(9890):427–51. doi: 10.1016/S0140-6736(13)60937-X 23746772

[15] United Nations Children’s Fund (UNICEF), World Health Organization, International Bank, for Reconstruction and Development/The World Bank. Levels and trends in child malnutrition: key findings of the 2021 edition of the joint child malnutrition estimates. Geneva: World Health Organization; 2021.

[16] WHO. World Health Organization. 2021 [cited 2021 Nov 29]. Obesity and overweight. Available from: https://www.who.int/news-room/fact-sheets/detail/obesity-and-overweight

[17] Baron EC, Hanlon C, Mall S, Honikman S, Breuer E, Kathree T, et al. Maternal mental health in primary care in five low- and middle-income countries: a situational analysis. BMC Health Serv Res. 2016 Feb 16;16(1):53. doi: 10.1186/s12913-016-1291-z 26880075 PMC4754802

[18] Headey D, Heidkamp R, Osendarp S, Ruel M, Scott N, Black R, et al. Impacts of COVID-19 on childhood malnutrition and nutrition-related mortality. The Lancet. 2020 Aug 22;396(10250):519–21. doi: 10.1016/S0140-6736(20)31647-0 32730743 PMC7384798

[19] Mertens E, Peñalvo JL. The Burden of Malnutrition and Fatal COVID-19: A Global Burden of Disease Analysis. Front Nutr. 2020;7:619850. doi: 10.3389/fnut.2020.619850 33553234 PMC7858665

[20] Martin. Goal 3: Ensure healthy lives and promote well-being for all at all ages [Internet]. United Nations Sustainable Development. 2021 [cited 2021 Nov 2]. Available from: https://www.un.org/sustainabledevelopment/health/

[21] WHO. Children: improving survival and well-being [Internet]. [cited 2021 Nov 2]. Available from: https://www.who.int/news-room/fact-sheets/detail/children-reducing-mortality

[22] Pelletier DL, Frongillo EA, Schroeder DG, Habicht JP. The effects of malnutrition on child mortality in developing countries. Bull World Health Organ. 1995;73(4):443–8. 7554015 PMC2486780

[23] Page MJ, McKenzie JE, Bossuyt PM, Boutron I, Hoffmann TC, Mulrow CD, et al. The PRISMA 2020 statement: an updated guideline for reporting systematic reviews. BMJ Online. 2021;372:n71–n71. doi: 10.1136/bmj.n71 33782057 PMC8005924

[24] World Health Organization. WHO Child Growth Standards: length/height-for-age, weight-for-age, weight-for-length, weight-for-height and body mass index-for-age: methods and development. WHO Press; 2006.

[25] Covidence systematic review software. Melbourne, Australia: Veritas Health Innovation;

[26] Country Classification–World Bank Data Help Desk [Internet]. [cited 2021 Nov 2]. Available from: https://datahelpdesk.worldbank.org/knowledgebase/topics/19280-country-classification

[27] Rotheram-Fuller EJ, Tomlinson M, Scheffler A, Weichle TW, Hayati Rezvan P, Comulada WS, et al. Maternal patterns of antenatal and postnatal depressed mood and the impact on child health at 3-years postpartum. J Consult Clin Psychol. 2018 Mar;86(3):218–30. doi: 10.1037/ccp0000281 29504791 PMC5842813

[28] Holm-Larsen CE, Madsen FK, Rogathi JJ, Manongi R, Mushi D, Meyrowitsch DW, et al. Postpartum depression and child growth in Tanzania: a cohort study. BJOG Int J Obstet Gynaecol. 2019 Apr;126(5):590–8. doi: 10.1111/1471-0528.15495 30290065

[29] Gordon S, Rotheram-Fuller E, Rezvan P, Stewart J, Christodoulou J, Tomlinson M. Maternal depressed mood and child development over the first five years of life in South Africa. J Affect Disord. 2021 Nov 1;294:346–56. doi: 10.1016/j.jad.2021.07.027 34315096

[30] Li X, Gao R, Dai X, Liu H, Zhang J, Liu X, et al. The association between symptoms of depression during pregnancy and low birth weight: a prospective study. Bmc Pregnancy Childbirth. 2020 Mar 6;20(1):147. doi: 10.1186/s12884-020-2842-1 32138708 PMC7059656

[31] Choi H, Lim JY, Lim NK, Ryu HM, Kwak DW, Chung JH, et al. Impact of pre-pregnancy body mass index and gestational weight gain on the risk of maternal and infant pregnancy complications in Korean women. Int J Obes 2005. 2022 Jan;46(1):59–67. doi: 10.1038/s41366-021-00946-8 34489525 PMC8748202

[32] Wu S, Gennings C, Wright RJ, Wilson A, Burris HH, Just AC, et al. Prenatal stress, methylation in inflammation-related genes, and adiposity measures in early childhood: The Programming Research in Obesity, Growth Environment and Social Stress cohort study. Psychosom Med. 2018 Jan;80(1):34–41. doi: 10.1097/PSY.0000000000000517 28787364 PMC5741481

[33] Wang H, Zhou H, Zhang Y, Wang Y, Sun J. Association of maternal depression with dietary intake, growth, and development of preterm infants: a cohort study in Beijing, China. Front Med. 2018 Oct;12(5):533–41. doi: 10.1007/s11684-017-0591-y 29181690

[34] Christodoulou J, Le Roux K, Tomlinson M, Le Roux IM, Katzen LS, Rotheram-Borus MJ. Perinatal maternal depression in rural South Africa: Child outcomes over the first two years. J Affect Disord. 2019 Mar 15;247:168–74. doi: 10.1016/j.jad.2019.01.019 30682695 PMC6377283

[35] Tome J, Mbuya MNN, Makasi RR, Ntozini R, Prendergast AJ, Dickin KL, et al. Maternal caregiving capabilities are associated with child linear growth in rural Zimbabwe. Matern Child Nutr. 2021 Apr;17(2):e13122. doi: 10.1111/mcn.13122 33350100 PMC7988870

[36] Odendaal HJ, Human M, van der Merwe C, Brink LT, Nel DG, Goldstein RD. The Association between Maternal Depression, Infant Characteristics and Need for Assistance in A Low-Income-Country. J Subst Abuse Alcohol. 2021;8(2):1090. PMC9718379 36466546

[37] Harper A, Rothberg A, Chirwa E, Mall S. Examining maternal depression, birthweight and linear growth: Findings from the South African National Income Dynamics Study. SAJCH South Afr J Child Health. 2022;16(4):215–9.

[38] Apriliana T, Keliat BA, Mustikasari M, Primasari Y. A contributing factor of maternal pregnancy depression in the occurrence of stunting on toddlers. J Public Health Res. 2021 Dec 2;11(2). doi: 10.4081/jphr.2021.2738 35244359 PMC8941310

[39] Shriyan P, Khetrapal S, van Schayck OCP, Babu GR. Maternal depressiveness and infant growth outcomes: Findings from the MAASTHI cohort study in India. J Psychosom Res. 2023 Jul;170:111378. doi: 10.1016/j.jpsychores.2023.111378 37244068 PMC7614640

[40] Kaiyo-Utete M, Langhaug L, Chingono A, Dambi JM, Magwali T, Henderson C, et al. Antenatal depression: Associations with birth and neonatal outcomes among women attending maternity care in Harare, Zimbabwe. PloS One. 2023;18(7):e0270873. doi: 10.1371/journal.pone.0270873 37418441 PMC10328234

[41] Baker-Henningham H, Powell C, Walker S, Grantham-McGregor S. Mothers of undernourished Jamaican children have poorer psychosocial functioning and this is associated with stimulation provided in the home. Eur J Clin Nutr. 2003;57(6):786–92. doi: 10.1038/sj.ejcn.1601611 12792663

[42] Surkan PJ, Ryan LM, Carvalho Vieira LM, Berkman LF, Peterson KE. Maternal social and pyschological conditions and physical growth in low-income children in Piauí, Northeast Brazil. Soc Sci Med 1982. 2007 Jan;64(2):375–88. 10.1016/j.socscimed.2006.09.021 17084498

[43] Surkan PJ, Kawachi I, Peterson KE. Childhood overweight and maternal depressive symptoms. J Epidemiol Community Health. 2008 May;62(5):e11. doi: 10.1136/jech.2007.065664 18431837

[44] Santos IS, Matijasevich A, Domingues MR, Barros AJD, Barros FCF. Long-lasting maternal depression and child growth at 4 years of age: a cohort study. J Pediatr. 2010 Sep;157(3):401–6. doi: 10.1016/j.jpeds.2010.03.008 20400093 PMC2937222

[45] Hassan BK, Werneck GL, Hasselmann MH. Maternal mental health and nutritional status of six-month-old infants. Rev Saúde Pública Online. 2016;50:7–7. doi: 10.1590/S1518-8787.2016050006237 27007683 PMC4794770

[46] Motlhatlhedi K, Setlhare V, Ganiyu AB, Firth JA. Association between depression in carers and malnutrition in children aged 6 months to 5 years. Afr J Prim Health Care Fam Med. 2017;9(1):a1270. doi: 10.4102/phcfm.v9i1.1270 28155288 PMC5291081

[47] Choi KW, Sikkema KJ, Vythilingum B, Geerts L, Faure SC, Watt MH, et al. Maternal childhood trauma, postpartum depression, and infant outcomes: Avoidant affective processing as a potential mechanism. J Affect Disord. 2017 Mar 15;211:107–15. doi: 10.1016/j.jad.2017.01.004 28110156

[48] Neamah HH, Sudfeld C, McCoy DC, Fink G, Fawzi WW, Masanja H, et al. Intimate partner violence, depression, and child growth and development. Pediatrics. 2018 Jul;142(1):1–10. 10.1542/peds.2017-3457 29891566

[49] Nguyen PH, Friedman J, Kak M, Menon P, Alderman H. Maternal depressive symptoms are negatively associated with child growth and development: Evidence from rural India. Matern Child Nutr. 2018 Oct;14(4):e12621. doi: 10.1111/mcn.12621 29770998 PMC6175434

[50] Sharmin KN, Sarwar N, Mumu SJ, Taleb DA, Flora MS. Postnatal depression and infant growth in an urban area of Bangladesh. Midwifery. 2019 Jul;74:57–67. doi: 10.1016/j.midw.2019.03.014 30927633

[51] Garman EC, Cois A, Tomlinson M, Rotheram-Borus MJ, Lund C. Course of perinatal depressive symptoms among South African women: associations with child outcomes at 18 and 36 months. Soc Psychiatry Psychiatr Epidemiol. 2019 Sep;54(9):1111–23. 30805694 10.1007/s00127-019-01665-2

[52] Mohammedahmed ASA, Koko AEA, Arabi AME, Ibrahim MA. Maternal depression, a hidden predictor for severe acute malnutrition in children aged 6–59 months: a case-control study at Omdurman Paediatrics Teaching Hospital, Sudan. Sudan J Paediatr. 2020;20(2):111–21. doi: 10.24911/SJP.106-1590606922 32817731 PMC7423302

[53] Emerson JA, Caulfield LE, Musafiri Kishimata E, Nzanzu JP, Doocy S. Maternal mental health symptoms are positively associated with child dietary diversity and meal frequency but not nutritional status in Eastern Democratic Republic of Congo. Public Health Nutr. 2020 Jul;23(10):1810–9. doi: 10.1017/S1368980019004087 32285766 PMC10200561

[54] Susiloretni K, Smith E, Suparmi, Marsum, Agustina R, Shankar A. The psychological distress of parents is associated with reduced linear growth of children: Evidence from a nationwide population survey. PLOS ONE. 2021 Oct 26;16(10). doi: 10.1371/journal.pone.0246725 34699530 PMC8547691

[55] He Q, Cheng G, He S, Tian G, Xie X, Jiang N, et al. Association between maternal postpartum depression and children’s physical growth in early childhood: a birth cohort study. Front Pediatr. 2023;11:1135876. doi: 10.3389/fped.2023.1135876 37565240 PMC10410140

[56] Brentani A, Fink G. Maternal depression and child development: Evidence from São Paulo’s Western Region Cohort Study. Rev Assoc Med Bras 1992. 2016 Sep;62(6):524–9. 27849229 10.1590/1806-9282.62.06.524

[57] Luchini C, Stubbs B, Solmi M, Veronese N. Assessing the quality of studies in meta-analyses: Advantages and limitations of the Newcastle Ottawa Scale. World J Meta-Anal. 2017 Aug 26;5(4):80–4.

[58] Bennett IM, Schott W, Krutikova S, Behrman JR. Maternal mental health, and child growth and development, in four low-income and middle-income countries. J Epidemiol Community Health. 2016;70(2):168–73. doi: 10.1136/jech-2014-205311 26359503 PMC5392254

[59] Higgins J, Li T, Deeks J, editors. Chapter 6: Choosing effect measures and computing estimates of effect. In: Cochrane Handbook for Systematic Reviews of Interventions version 64 (updated August 2023) [Internet]. Cochrane; 2023. Available from: www.training.cochrane.org/handbook

[60] DerSimonian R, Laird N. Meta-analysis in clinical trials. Control Clin Trials. 1986 Sep;7(3):177–88. doi: 10.1016/0197-2456(86)90046-2 3802833

[61] Knapp G, Hartung J. Improved tests for a random effects meta-regression with a single covariate. Stat Med. 2003 Sep 15;22(17):2693–710. doi: 10.1002/sim.1482 12939780

[62] Egger M, Smith GD, Schneider M, Minder C. Bias in meta-analysis detected by a simple, graphical test. BMJ. 1997 Sep 13;315(7109):629–34. doi: 10.1136/bmj.315.7109.629 9310563 PMC2127453

[63] Higgins JPT, Thompson SG, Deeks JJ, Altman DG. Measuring inconsistency in meta-analyses. BMJ. 2003 Sep 6;327(7414):557–60. doi: 10.1136/bmj.327.7414.557 12958120 PMC192859

[64] Higgins J, Thomas J, Chadler J, Cumpston M, Page M, Welch V, editors. Cochrane Handbook for Systematic Reviews of Interventions version 6.3 [Internet]. Cochrane; 2022. Available from: www.training.cochrane.org/handbook

[65] Stata Statistical Software. College Station, TX: StataCorp LLC; 2021. (StataCorp).

[66] Prado EL, Yakes Jimenez E, Vosti S, Stewart R, Stewart CP, Somé J, et al. Path analyses of risk factors for linear growth faltering in four prospective cohorts of young children in Ghana, Malawi and Burkina Faso. BMJ Glob Health. 2019;4(1):e001155. doi: 10.1136/bmjgh-2018-001155 30775005 PMC6350712

[67] Nasreen HE, Nahar Kabir Z, Forsell Y, Edhborg M. Impact of maternal depressive symptoms and infant temperament on early infant growth and motor development: Results from a population based study in Bangladesh. J Affect Disord. 2013 Apr 5;146(2):254–61. doi: 10.1016/j.jad.2012.09.013 23063237

[68] Patel V, DeSouza N, Rodrigues M. Postnatal depression and infant growth and development in low income countries: a cohort study from Goa, India. Arch Dis Child. 2003 Jan;88(1):34–7. doi: 10.1136/adc.88.1.34 12495957 PMC1719257

[69] Rahman A, Iqbal Z, Bunn J, Lovel H, Harrington R. Impact of maternal depression on infant nutritional status and illness. Arch Gen Psychiatry. 2004 Sep;61(9):946–52. 15351773 10.1001/archpsyc.61.9.946

[70] Rahman A, Lovel H, Bunn J, Iqbal Z, Harrington R. Mothers’ mental health and infant growth: a case-control study from Rawalpindi, Pakistan. Child Care Health Dev. 2004 Jan;30(1):21–7. doi: 10.1111/j.1365-2214.2004.00382.x 14678308

[71] Anoop S, Saravanan B, Joseph A, Cherian A, Jacob KS. Maternal depression and low maternal intelligence as risk factors for malnutrition in children: A community based case-control study from South India. Arch Dis Child. 2004;89(4):325–9. doi: 10.1136/adc.2002.009738 15033840 PMC1719856

[72] Tomlinson M. Parenting and poverty: A complex interaction. In: Tyano S, Keren M, Herrman H, Cox J, editors. Parenthood and mental health: A bridge between infant and adult psychiatry [Internet]. Wiley-Blackwell; 2010. p. 337–48. (World Psychiatric Association evidence and experience in psychiatry). Available from: http://ezp-prod1.hul.harvard.edu/login?url=http://search.ebscohost.com/login.aspx?direct=true&db=psyh&AN=2010-10943-030&site=ehost-live&scope=site

[73] Surkan PJ, Kawachi I, Ryan LM, Berkman LF, Vieira LMC, Peterson KE. Maternal depressive symptoms, parenting self-efficacy, and child growth. Am J Public Health. 2008 Jan;98(1):125–32. doi: 10.2105/AJPH.2006.108332 18048782 PMC2156066

[74] Adewuya AO, Ola BO, Aloba OO, Mapayi BM, Okeniyi JAO. Impact of postnatal depression on infants’ growth in Nigeria. J Affect Disord. 2008 May;108(1–2):191–3. doi: 10.1016/j.jad.2007.09.013 17997163

[75] Avan B, Richter LM, Ramchandani PG, Norris SA, Stein A. Maternal postnatal depression and children’s growth and behaviour during the early years of life: exploring the interaction between physical and mental health. Arch Dis Child. 2010 Sep;95(9):690–5. doi: 10.1136/adc.2009.164848 20660522

[76] Ndokera R, MacArthur C. The relationship between maternal depression and adverse infant health outcomes in Zambia: A cross-sectional feasibility study. Child Care Health Dev. 2011 Jan;37(1):74–81. doi: 10.1111/j.1365-2214.2010.01129.x 20637022

[77] Kaaya S, Garcia ME, Li N, Lienert J, Twayigize W, Spiegelman D, et al. Association of maternal depression and infant nutritional status among women living with HIV in Tanzania. Matern Child Nutr. 2016 Jul;12(3):603–13. doi: 10.1111/mcn.12154 25382710 PMC6240341

[78] Madeghe BA, Kimani VN, Vander Stoep A, Nicodimos S, Kumar M. Postpartum depression and infant feeding practices in a low income urban settlement in Nairobi-Kenya. BMC Res Notes. 2016 Dec 8;9(1):506. doi: 10.1186/s13104-016-2307-9 27931248 PMC5146885

[79] Upadhyay AK, Srivastava S. Effect of pregnancy intention, postnatal depressive symptoms and social support on early childhood stunting: Findings from India. BMC Pregnancy Childbirth [Internet]. 2016;16(1). Available from: https://www.embase.com/search/results?subaction=viewrecord&id=L613897116&from=export doi: 10.1186/s12884-016-0909-9 27184026 PMC4867507

[80] Saeed Q, Shah N, Inam S, Shafique K. Maternal depressive symptoms and child nutritional status: A cross-sectional study in socially disadvantaged Pakistani community. J Child Health Care Prof Work Child Hosp Community. 2017;21(3):331–42. doi: 10.1177/1367493517721063 29119823

[81] Lima TFM, Maciel WM, Alencar MN de, Cruz JA da S, Carvalho CA de, Silva AAM da. Association between maternal depressive symptoms with child malnutrition or child excess weight. Rev Bras Saúde Mater Infant Online. 2017 Jul;17(3):591–601.

[82] Wemakor A, Iddrisu H. Maternal depression does not affect complementary feeding indicators or stunting status of young children (6–23 months) in Northern Ghana. BMC Res Notes. 2018;11(408):(25 June 2018). 10.1186/s13104-018-3528-x PMC6019211 29941023

[83] Joshi MN, Raut AV. Maternal depression and its association with responsive feeding and nutritional status of infants: A cross-sectional study from a rural medical college in central India. J Postgrad Med. 2019;65(4):212–8. doi: 10.4103/jpgm.JPGM_479_18 31204726 PMC6813688

[84] Ahmadi Gharaei H, Nematollahi S, Moameri H, Madani A, Parsaeian M, Holakouie-Naieni K. Effect of maternal mental health during pregnancy on infant growth at six months of age in Suburban communities in South of Iran. Med J Islam Repub Iran. 2020;34:157. doi: 10.47176/mjiri.34.157 33500884 PMC7813148

[85] Anato A, Baye K, Tafese Z, Stoecker BJ. Maternal depression is associated with child undernutrition: A cross-sectional study in Ethiopia. Matern Child Nutr. 2020 Jul;16(3):e12934. doi: 10.1111/mcn.12934 31833231 PMC7296785

[86] Ricci H, Nakiranda R, Malan L, Kruger HS, Visser M, Ricci C, et al. Association between maternal postpartum depressive symptoms, socioeconomic factors, and birth outcomes with infant growth in South Africa. Sci Rep. 2023 Apr 7;13(1):5696. doi: 10.1038/s41598-023-32653-x 37029213 PMC10080513

[87] Saleh A, Perumal N, Muhihi A, Duggan CP, Ulenga N, Al-Beity FMA, et al. Associations Between Social Support and Symptoms of Antenatal Depression with Infant Growth and Development Among Mothers Living with HIV in Tanzania. AIDS Behav. 2023 May 4; doi: 10.1007/s10461-023-04073-5 37140717 PMC10592034

[88] Rahman A, Iqbal Z, Bunn J, Lovel H, Harrington R. Impact of maternal depression on infant nutritional status and illness: A cohort study. Arch Gen Psychiatry. 2004;61(9):946–52. doi: 10.1001/archpsyc.61.9.946 15351773

[89] Lingam R, Gupta P, Zafar S, Hill Z, Yousafzai A, Iyengar S, et al. Understanding care and feeding practices: building blocks for a sustainable intervention in India and Pakistan. Ann N Y Acad Sci. 2014;1308(1):204–17. doi: 10.1111/nyas.12326 24392960

[90] WHO. Fact sheets—Malnutrition [Internet]. [cited 2021 Nov 2]. Available from: https://www.who.int/news-room/fact-sheets/detail/malnutrition

[91] Audelo J, Kogut K, Harley KG, Rosas LG, Stein L, Eskenazi B. Maternal Depression and Childhood Overweight in the CHAMACOS Study of Mexican-American Children. Matern Child Health J. 2016;20(7):1405–14. doi: 10.1007/s10995-016-1937-9 27007986

[92] Wang L, Anderson JL, Dalton WTI, Wu T, Liu X, Zheng S, et al. Maternal depressive symptoms and the risk of overweight in their children. Matern Child Health J. 2013 Jul;17(5):940–8. doi: 10.1007/s10995-012-1080-1 22833333

[93] Constantino B, Silva JC da, Michels M de S, Mastroeni MF. Symptoms of maternal depression and anxiety and their effect on the child’s weight status four years after delivery. Early Child Dev Care [Internet]. 2019 Oct 15; Available from: http://ezp-prod1.hul.harvard.edu/login?url=http://search.ebscohost.com/login.aspx?direct=true&db=psyh&AN=2019-61933-001&site=ehost-live&scope=site

[94] Sahoo K, Sahoo B, Choudhury AK, Sofi NY, Kumar R, Bhadoria AS. Childhood obesity: causes and consequences. J Fam Med Prim Care. 2015;4(2):187–92. doi: 10.4103/2249-4863.154628 25949965 PMC4408699

[95] Tzioumis E, Adair LS. Childhood dual burden of under- and over-nutrition in low- and middle-income countries: a critical review. Food Nutr Bull. 2014 Jun;35(2):230–43. doi: 10.1177/156482651403500210 25076771 PMC4313560

[96] Robertson E, Grace S, Wallington T, Stewart DE. Antenatal risk factors for postpartum depression: a synthesis of recent literature. Gen Hosp Psychiatry. 2004 Aug;26(4):289–95. doi: 10.1016/j.genhosppsych.2004.02.006 15234824

[97] Wisner KL, Sit DKY, McShea MC, Rizzo DM, Zoretich RA, Hughes CL, et al. Onset Timing, Thoughts of Self-harm, and Diagnoses in Postpartum Women With Screen-Positive Depression Findings. JAMA Psychiatry. 2013 May 1;70(5):490–8. doi: 10.1001/jamapsychiatry.2013.87 23487258 PMC4440326

[98] Scorza P, Owusu-Agyei S, Asampong E, Wainberg ML. The expression of perinatal depression in rural Ghana. Int J Cult Ment Health. 2015;8(4):370–81. doi: 10.1080/17542863.2015.1037849 26539247 PMC4629504

[99] World Health Assembly 65. Global burden of mental disorders and the need for a comprehensive, coordinated response from health and social sectors at the country level: report by the Secretariat [Internet]. World Health Organization; 2012 [cited 2021 Nov 2]. Report No.: A65/10. Available from: https://apps.who.int/iris/handle/10665/78898

[100] Eappen BS, Aguilar M, Ramos K, Contreras C, Prom MC, Scorza P, et al. Preparing to launch the ‘Thinking Healthy Programme’ perinatal depression intervention in Urban Lima, Peru: experiences from the field. Glob Ment Health. 2018;5:e41–e41. doi: 10.1017/gmh.2018.32 30637114 PMC6315282

[101] Howard LM, Piot P, Stein A. No health without perinatal mental health. Lancet Br Ed. 2014;384(9956):1723–4. 10.1016/S0140-6736(14)62040-7 25455235

