# Supplementary material for: Perinatal depression and adverse child growth outcomes in low-income and middle-income countries (LMICs): A systematic review and meta-analysis
Source: PLOS Glob Public Health. 2024 Oct 28;4(10):e0003586. doi: 10.1371/journal.pgph.0003586 (PMC11516009; doi:10.1371/journal.pgph.0003586)
Supplement: S1 Text — (DOCX) [file pgph.0003586.s001.docx]

**S1 Text. The structured search strategy utilized.**

PubMed, EMBASE, Web of Science, CINAHL Plus, Google Scholar, Global Health Database (EBSCO), WHO Regional Databases (Global Index Medicus), PsycINFO, and LILACS.

**A: PubMed**

("Depression, Postpartum"[Mesh] OR (("Depressive Disorder"[Mesh] OR "Depression"[mesh] OR depression[tiab] OR depressed[tiab] OR depressive[tiab]) AND ("Pregnancy"[Mesh] OR "Pregnancy Complications"[Mesh:NoExp] OR "Pregnant Women"[Mesh] OR pregnant[tiab] OR pregnancy[tiab] OR perinatal[tiab] OR peripartum[tiab] OR antenatal[tiab] OR postpartum[tiab] OR post-partum[tiab] OR post partum[tiab])) OR antenatal depression[tiab] OR prenatal depression[tiab] OR pre natal depression[tiab] OR perinatal depression[tiab] OR peri natal depression[tiab] OR puerperal depression[tiab] OR postnatal depression[tiab] OR post natal depression[tiab] OR postpartum depression[tiab] OR post-partum depression[tiab] OR maternal depression[tiab] OR maternal depressive symptoms[tiab] OR parent depression[tiab] OR parent depressive symptoms[tiab] OR maternal postpartum depression[tiab] OR maternal postpartum depressive symptoms[tiab] OR maternal postnatal depression[tiab] OR maternal postnatal depressive symptoms[tiab])

AND

("Growth Disorders"[Mesh] OR growth outcome*[tiab] OR stunt*[tiab] OR wasting[tiab] OR kwashiorkor*[tiab] OR weight for age[tiab] OR bmi for age[tiab] OR “pediatric obesity”[mesh] OR “Child Development”[Mesh] OR ((("child"[MeSH Terms] OR child*[tiab]) AND ("growth and development"[tiab] OR (growth[tiab] AND development[tiab]) OR “growth and development”[Mesh]) ) AND ("reference standards"[MeSH Terms] OR (reference*[tiab] AND standard*[tiab])) ) OR (("child"[MeSH Terms] OR child*[tiab] OR “infant”[mesh] OR infant* OR baby*[tiab]) AND (weight*[tiab] OR “overweight”[mesh] OR “obesity”[mesh] OR body mass index [mesh] OR “adiposity”[mesh])))

AND

("Developing Countries"[mesh] OR “Poverty”[mesh] OR developing countr*[tiab] OR developing nation*[tiab] OR less developed countr*[tiab] OR less developed nation*[tiab] OR third world nation*[tiab] OR third world countr*[tiab] OR under developed nation*[tiab] OR underdeveloped nation*[tiab] OR under developed countr*[tiab] OR underdeveloped nation*[tiab] OR underserved countr*[tiab] OR underserved area*[tiab] OR developing econom*[tiab] OR resource poor[tiab] OR resource limit*[tiab] OR limited resource*[tiab] OR limiting resource*[tiab] OR low resource[tiab] OR resource constrain*[tiab] OR constrained resource*[tiab] OR middle income countr*[tiab] OR middle income nation*[tiab] OR low income countr*[tiab] OR low income nation*[tiab] OR poor countr*[tiab] OR poor nation*[tiab] OR lmic[tiab] OR lmics[tiab] OR "Africa"[mesh] OR "Asia"[mesh] OR "South America"[mesh] OR "Latin America"[mesh] OR "Central America"[mesh] OR africa[tiab] OR asia[tiab] OR south america*[tiab] OR latin america*[tiab] OR central america*[tiab] OR afghanistan*[tiab] OR albania*[tiab] OR algeria*[tiab] OR samoa*[tiab] OR angola*[tiab] OR argentina*[tiab] OR armenia*[tiab] OR azerbaijan*[tiab] OR bangladesh*[tiab] OR belarus*[tiab] OR belize*[tiab] OR benin*[tiab] OR bhutan*[tiab] OR bolivia*[tiab] OR bosnia*[tiab] OR herzegovina*[tiab] OR botswana*[tiab] OR brazil*[tiab] OR bulgaria*[tiab] OR burkin*[tiab] OR burundi*[tiab] OR cabo verde*[tiab] OR cape verde*[tiab] OR cambodia*[tiab] OR cameroon*[tiab] OR central africa*[tiab] OR chad*[tiab] OR china[tiab] OR chinese[tiab] OR colombia*[tiab] OR comoros[tiab] OR comorian*[tiab] OR congo*[tiab] OR costa rica*[tiab] OR côte d'ivoire[tiab] OR ivorian*[tiab] OR ivory coast[tiab] OR cuba*[tiab] OR djibouti*[tiab] OR dominica*[tiab] OR ecuador*[tiab] OR egypt*[tiab] OR el salvador[tiab] OR salvadoran*[tiab] OR guinea*[tiab] OR eritrea*[tiab] OR eswatini*[tiab] OR swaziland*[tiab] OR ethiopia*[tiab] OR fiji*[tiab] OR gabon*[tiab] OR gambia*[tiab] OR republic of georgia[tiab] OR ghana*[tiab] OR grenada*[tiab] OR guatemala*[tiab] OR guyana*[tiab] OR haiti*[tiab] OR honduras*[tiab] OR india*[tiab] OR indonesia*[tiab] OR iran*[tiab] OR iraq*[tiab] OR jamaica*[tiab] OR jordan*[tiab] OR kazakhstan*[tiab] OR kenya*[tiab] OR kiribati*[tiab] OR democratic people s republic of korea[tiab] OR north korea*[tiab] OR kosovo[tiab] OR kosovar*[tiab] OR kyrgyz*[tiab] OR lao[tiab] OR laos[tiab] OR laotian*[tiab] OR lebanon*[tiab] OR lesotho[tiab] OR liberia*[tiab] OR libya*[tiab] OR madagascar*[tiab] OR malawi*[tiab] OR malaysia*[tiab] OR maldives*[tiab] OR mali[tiab] OR malian[tiab] OR malians[tiab] OR marshall island*[tiab] OR mauritania*[tiab] OR mexico[tiab] OR mexican*[tiab] OR micronesia*[tiab] OR moldova*[tiab] OR mongolia*[tiab] OR montenegr*[tiab] OR morocc*[tiab] OR mozambi*[tiab] OR myanmar*[tiab] OR namibia*[tiab] OR nepal*[tiab] OR nicaragua*[tiab] OR niger[tiab] OR nigerien*[tiab] OR nigeria*[tiab] OR macedonia*[tiab] OR pakistan*[tiab] OR paragua*[tiab] OR peru*[tiab] OR philippines[tiab] OR filipino*[tiab] OR russia*[tiab] OR rwanda*[tiab] OR são tomé and principe[tiab] OR senegal*[tiab] OR serbia*[tiab] OR sierra leone*[tiab] OR solomon island*[tiab] OR somalia*[tiab] OR south africa*[tiab] OR sudan*[tiab] OR sri lank*[tiab] OR st lucia*[tiab] OR saint lucia*[tiab] OR st. vincent*[tiab] OR saint vincent*[tiab] OR grenadines[tiab] OR surinam*[tiab] OR syria*[tiab] OR tajikistan*[tiab] OR tanzania*[tiab] OR thailand[tiab] OR thai[tiab] OR thais[tiab] OR timor*[tiab] OR togo[tiab] OR togolese*[tiab] OR tonga*[tiab] OR tunisia*[tiab] OR turkey[tiab] OR turks[tiab] OR turkish[tiab] OR turkmenistan*[tiab] OR tuvalu*[tiab] OR uganda*[tiab] OR ukrain*[tiab] OR uzbekistan*[tiab] OR vanuatu*[tiab] OR venezuela*[tiab] OR vietnam*[tiab] OR viet nam*[tiab] OR west bank[tiab] OR gaza[tiab] OR gazan*[tiab] OR yemen*[tiab] OR zambia*[tiab] OR zimbabwe*[tiab])

**B. Embase**

#1 ('perinatal depression'/exp OR ('pregnant woman'/exp AND 'depression'/exp) OR ((postpartum OR 'post partum' OR perinatal OR pre-natal OR ‘pre natal’ OR postnatal OR prenatal OR 'peri natal' OR 'post natal' OR maternal) AND (depression OR depressive)):ab,kw,ti)

AND

#2 ('stunting'/exp OR 'stunting' OR 'failure to thrive'/exp OR 'failure to thrive' OR 'failure to thrive':ab,ti,kw OR 'short stature'/exp OR 'short stature' OR 'short stature':ab,ti,kw OR 'short body stature'/exp OR 'short body stature' OR 'short body stature':ab,ti,kw OR 'small stature'/exp OR 'small stature' OR 'small stature':ab,ti,kw OR 'malnutrition inflammation atherosclerosis syndrome'/exp OR 'malnutrition inflammation atherosclerosis syndrome' OR 'malnutrition':ab,ti,kw OR 'protein deficiency'/exp OR 'protein deficiency' OR 'protein deficiency':ab,ti,kw OR 'nutritional disorder'/exp OR 'nutritional disorder' OR 'nutritional disorder':ab,ti,kw OR 'infant nutrition disorder':ab,ti,kw OR 'nutrition disorder, infant':ab,ti,kw OR 'nutrition disorders, infant':ab,ti,kw OR 'infant overnutrition':ab,ti,kw OR 'overnutrition, infant':ab,ti,kw OR 'infant malnutrition':ab,ti,kw OR 'malnutrition in infant':ab,ti,kw OR 'malnutrition in infants':ab,ti,kw OR 'malnutrition, infant':ab,ti,kw OR 'infantile malnutrition':ab,ti,kw OR 'malnutrition, infantile':ab,ti,kw OR 'deficient nutrition'/exp OR 'deficient nutrition' OR 'deficient nutrition':ab,ti,kw OR 'malnourishment'/exp OR 'malnourishment' OR 'malnourishment':ab,ti,kw OR 'severe acute malnutrition'/exp OR 'severe acute malnutrition' OR 'severe acute malnutrition':ab,ti,kw OR 'underfeeding'/exp OR 'underfeeding' OR 'underfeeding':ab,ti,kw OR 'undernourishment'/exp OR 'undernourishment' OR 'undernourishment':ab,ti,kw OR 'deficiency, protein'/exp OR 'deficiency, protein' OR 'deficiency, protein':ab,ti,kw OR 'dietary protein deficiency'/exp OR 'dietary protein deficiency' OR 'dietary protein deficiency':ab,ti,kw OR 'alimentary deficiency'/exp OR 'alimentary deficiency' OR 'alimentary deficiency':ab,ti,kw OR 'defective diet'/exp OR 'defective diet' OR 'defective diet':ab,ti,kw OR 'deficiency disease'/exp OR 'deficiency disease' OR 'deficiency disease':ab,ti,kw OR 'deficiency diseases'/exp OR 'deficiency diseases' OR 'deficiency diseases':ab,ti,kw OR 'deficiency, nutritional'/exp OR 'deficiency, nutritional' OR 'deficiency, nutritional':ab,ti,kw OR 'deficient diet'/exp OR 'deficient diet' OR 'deficient diet':ab,ti,kw OR 'diet deficiency'/exp OR 'diet deficiency' OR 'diet deficiency':ab,ti,kw OR 'diet insufficiency'/exp OR 'diet insufficiency' OR 'diet insufficiency':ab,ti,kw OR 'diet, defective'/exp OR 'diet, defective' OR 'diet, defective':ab,ti,kw OR 'dietary deficiency'/exp OR 'dietary deficiency' OR 'dietary deficiency':ab,ti,kw OR 'dietary insufficiency'/exp OR 'dietary insufficiency' OR 'dietary insufficiency':ab,ti,kw OR 'food deficiency'/exp OR 'food deficiency' OR 'micronutrient deficiency'/exp OR 'micronutrient deficiency' OR 'micronutrient deficiency':ab,ti,kw OR 'micronutrient insufficiency'/exp OR 'micronutrient insufficiency' OR 'micronutrient insufficiency':ab,ti,kw OR 'nutrient deficiency'/exp OR 'nutrient deficiency' OR 'nutrient deficiency':ab,ti,kw OR 'nutritional deficiency'/exp OR 'nutritional deficiency' OR 'nutritional deficiency':ab,ti,kw OR 'nutrition deficiency'/exp OR 'nutrition deficiency' OR 'nutrition deficiency':ab,ti,kw OR 'nutritional deficit'/exp OR 'nutritional deficit' OR 'nutritional deficit':ab,ti,kw OR 'nutritive deficiency'/exp OR 'nutritive deficiency' OR 'nutritive deficiency':ab,ti,kw OR 'overweight':ab,ti,kw OR 'obesity'/exp OR 'obesity' OR 'wasting':ab,ti,kw OR ‘adipos*’:ab,ti,kw OR ‘body weight’:ab,ti,kw OR ‘fat overload syndrome’:ab,ti,kw OR ‘nutritional obesity’:ab,ti,kw)

AND

#3 ('developing country'/exp OR 'low income country'/de OR 'middle income country'/de OR ((developing OR 'less developed' OR 'third world' OR 'under developed' OR 'middle income' OR 'low income' OR underserved OR 'under served' OR deprived OR poor*) NEAR/1 (countr* OR nation* OR state* OR population* OR area*)):ab,kw,ti OR (resource* NEAR/2 (poor OR limiting OR limited OR low OR constrain*)):ab,kw,ti OR 'Africa'/de OR 'Africa south of the Sahara'/de OR 'North Africa'/de OR 'Asia'/de OR 'Far East'/de OR 'Middle East'/de OR 'South Asia'/de OR 'South and Central America'/de OR 'South America'/de OR 'Central America'/de OR 'Caribbean'/de OR (lmic OR lmics OR africa OR asia OR 'south america*' OR 'latin america*' OR 'central america*' OR afghanistan* OR albania* OR algeria* OR samoa* OR angola* OR argentina* OR armenia* OR azerbaijan* OR bangladesh* OR belarus* OR belize* OR benin* OR bhutan* OR bolivia* OR bosnia* OR herzegovina* OR botswana* OR brazil* OR bulgaria* OR burkin* OR burundi* OR 'cabo verde*' OR 'cape verde*' OR cambodia* OR cameroon* OR 'central africa*' OR chad* OR china OR chinese OR colombia* OR comoros OR comorian* OR congo* OR costa rica* OR 'cote d ivoire*' OR ivorian* OR 'ivory coast' OR cuba* OR djibouti* OR dominica* OR ecuador* OR egypt* OR 'el salvador' OR salvadoran* OR guinea* OR eritrea* OR eswatini* OR swaziland* OR ethiopia* OR fiji* OR gabon* OR gambia* OR 'republic of georgia' OR ghana* OR grenada* OR guatemala* OR guyana* OR haiti* OR honduras* OR india* OR indonesia* OR iran* OR iraq* OR jamaica* OR jordan* OR kazakhstan* OR kenya* OR kiribati* OR 'democratic people s republic of korea' OR 'north korea*' OR kosovo OR kosovar* OR kyrgyz* OR lao OR laos OR laotian* OR lebanon* OR lesotho OR liberia* OR libya* OR madagascar* OR malawi* OR malaysia* OR maldives* OR mali OR malian OR malians OR 'marshall island*' OR mauritania* OR mexico OR mexican* OR micronesia* OR moldova* OR mongolia* OR montenegr* OR morocc* OR mozambi* OR myanmar* OR namibia* OR nepal* OR nicaragua* OR niger OR nigerien* OR nigeria* OR macedonia* OR pakistan* OR paragua* OR peru* OR philippines OR filipino* OR russia* OR rwanda* OR 'sao tome*' OR senegal* OR serbia* OR 'sierra leone*' OR 'solomon island*' OR somalia* OR 'south africa*' OR sudan* OR 'sri lank*' OR 'st lucia*' OR 'saint lucia*' OR 'st vincent*' OR 'saint vincent*' OR grenadines OR surinam* OR syria* OR tajikistan* OR tanzania* OR thailand OR thai OR thais OR timor* OR togo OR togolese* OR tonga* OR tunisia* OR turkey OR turks OR turkish OR turkmenistan* OR tuvalu* OR uganda* OR ukrain* OR uzbekistan* OR vanuatu* OR venezuela* OR vietnam* OR 'viet nam*' OR 'west bank' OR gaza OR gazan* OR yemen* OR zambia* OR zimbabwe*):ab,kw,ti)

**C. Web of Science**

#1 TOPIC: (antenatal depression OR prenatal depression OR pre natal depression OR perinatal depression OR peri natal depression OR puerperal depression OR postnatal depression OR post natal depression OR postpartum depression OR postpartum depression OR maternal depression OR maternal depressive symptoms OR parent depression OR parent depressive symptoms OR maternal postpartum depression OR maternal postpartum depressive symptoms OR maternal postnatal depression OR maternal postnatal depressive symptoms)

#2 TOPIC: (growth outcome* OR fetal growth OR stunt* OR stunted growth OR growth disorder* OR wasting OR weight for age OR bmi for age OR ((child* OR infant* OR baby* OR pediatr*) AND (nutritional disorder OR nutrition disorder OR overnutrition OR undernutrition OR malnutrition OR deficienc* OR malnourish* OR underfeed*)) OR (child* AND ("growth and development" OR growth) AND (reference AND standard*))) OR kwashiorkor* OR (child* AND (growth and development OR (growth AND development)) AND (reference standard* OR (reference* AND standard*))) OR ((child* OR infant* OR baby* OR pediatr*) AND (weight* OR overweight OR obesity OR body mass index OR bmi OR adiposit*))

#3 TOPIC: (developing countr* OR developing nation* OR less developed countr* OR less developed nation* OR third world nation* OR third world countr* OR under developed nation* OR underdeveloped nation* OR under developed countr* OR underdeveloped nation* OR underserved countr* OR underserved area* OR developing econom* OR resource poor OR resource limit* OR limited resource* OR limiting resource* OR low resource OR resource constrain* OR constrained resource* OR middle income countr* OR middle income nation* OR low income countr* OR low income nation* OR poor countr* OR poor nation* OR lmic OR lmics OR africa OR asia OR south america* OR latin america* OR central america* OR afghanistan* OR albania* OR algeria* OR samoa* OR angola* OR argentina* OR armenia* OR azerbaijan* OR bangladesh* OR belarus* OR belize* OR benin* OR bhutan* OR bolivia* OR bosnia* OR herzegovina* OR botswana* OR brazil* OR bulgaria* OR burkin* OR burundi* OR cabo verde* OR cape verde* OR cambodia* OR cameroon* OR central africa* OR chad* OR china OR chinese OR colombia* OR comoros OR comorian* OR congo* OR costa rica* OR côte d'ivoire OR ivorian* OR ivory coast OR cuba* OR djibouti* OR dominica* OR ecuador* OR egypt* OR el salvador OR salvadoran* OR guinea* OR eritrea* OR eswatini* OR swaziland* OR ethiopia* OR fiji* OR gabon* OR gambia* OR republic of georgia OR ghana* OR grenada* OR guatemala* OR guyana* OR haiti* OR honduras* OR india* OR indonesia* OR iran* OR iraq* OR jamaica* OR jordan* OR kazakhstan* OR kenya* OR kiribati* OR democratic people's republic of korea OR north korea* OR kosovo OR kosovar* OR kyrgyz* OR lao OR laos OR laotian* OR lebanon* OR lesotho OR liberia* OR libya* OR madagascar* OR malawi* OR malaysia* OR maldives* OR mali OR malian OR malians OR marshall island* OR mauritania* OR mexico OR mexican* OR micronesia* OR moldova* OR mongolia* OR montenegr* OR morocc* OR mozambi* OR myanmar* OR namibia* OR nepal* OR nicaragua* OR niger OR nigerien* OR nigeria* OR macedonia* OR pakistan* OR paragua* OR peru* OR philippines OR filipino* OR russia* OR rwanda* OR são tomé and principe OR senegal* OR serbia* OR sierra leone* OR solomon island* OR somalia* OR south africa* OR sudan* OR sri lank* OR st lucia* OR saint lucia* OR st. vincent* OR saint vincent* OR grenadines OR surinam* OR syria* OR tajikistan* OR tanzania* OR thailand OR thai OR thais OR timor* OR togo OR togolese* OR tonga* OR tunisia* OR turkey OR turks OR turkish OR turkmenistan* OR tuvalu* OR uganda* OR ukrain* OR uzbekistan* OR vanuatu* OR venezuela* OR vietnam* OR viet nam* OR west bank OR gaza OR gazan* OR yemen* OR zambia* OR zimbabwe*)

**D. Global Health Database (EBSCO)**

( (TI (depression AND (Postpartum OR "post partum" OR post-partum OR peri natal OR perinatal OR "peri natal" OR antenatal OR ante-natal OR "ante natal" OR pregn* OR maternal))) OR (AB (depression AND (Postpartum OR "port partum" OR post-partum OR peri natal OR perinatal OR "peri natal" OR antenatal OR ante-natal OR "ante natal" OR pregn* OR maternal))) OR (SU (depression AND (Postpartum OR "port partum" OR post-partum OR peri natal OR perinatal OR "peri natal" OR antenatal OR ante-natal OR "ante natal" OR pregn* OR maternal))))

AND

( (TI (Growth Disorders OR growth outcome* wasting OR weight for age OR bmi for age OR weight-for-height OR weight-for-age OR length-for-age OR weight-for-length)) OR (SU (Growth Disorders OR growth outcome* OR stunt* OR wasting OR weight for age OR bmi for age OR weight-for-height OR weight-for-age OR length-for-age OR weight-for-length)) OR (AB (Growth Disorders OR growth outcome* OR stunt* OR wasting OR weight for age OR bmi for age OR weight-for-height OR weight-for-age OR length-for-age OR weight-for-length)) )

AND

(( TI(“developing countr*” OR “developing nation*” OR “low-income countr*” OR “low income countr*” OR “low-income nation*” OR “low income nation*” OR “third world countr*” OR “third-world countr*” OR “third world nation*” OR “third-world nation*” OR “under-developed countr*” OR “under developed countr*” OR “under developed nation*” OR “under-developed nation*” OR “middle-income countr*” OR “middle-income nation*” OR “middle-income nation*” OR “middle income countr*” OR underserved area* OR developing econom* OR resource poor OR resource limit* OR limited resource* OR limiting resource* OR low resource OR resource constrain* OR constrained resource* OR middle income countr* OR middle income nation* OR low income countr* OR low income nation* OR poor countr* OR poor nation* OR lmic OR lmics OR africa OR asia OR south america* OR latin america* OR central america* OR afghanistan* OR albania* OR algeria* OR samoa* OR angola* OR argentina* OR armenia* OR azerbaijan* OR bangladesh* OR belarus* OR belize* OR benin* OR bhutan* OR bolivia* OR bosnia* OR herzegovina* OR botswana* OR brazil* OR bulgaria* OR burkin* OR burundi* OR cabo verde* OR cape verde* OR cambodia* OR cameroon* OR central africa* OR chad* OR china OR chinese OR colombia* OR comoros OR comorian* OR congo* OR costa rica* OR côte d'ivoire OR ivorian* OR ivory coast OR cuba* OR djibouti* OR dominica* OR ecuador* OR egypt* OR el salvador OR salvadoran* OR guinea* OR eritrea* OR eswatini* OR swaziland* OR ethiopia* OR fiji* OR gabon* OR gambia* OR republic of georgia OR ghana* OR grenada* OR guatemala* OR guyana* OR haiti* OR honduras* OR india* OR indonesia* OR iran* OR iraq* OR jamaica* OR jordan* OR kazakhstan* OR kenya* OR kiribati* OR democratic people's republic of korea OR north korea* OR kosovo OR kosovar* OR kyrgyz* OR lao OR laos OR laotian* OR lebanon* OR lesotho OR liberia* OR libya* OR madagascar* OR malawi* OR malaysia* OR maldives* OR mali OR malian OR malians OR marshall island* OR mauritania* OR mexico OR mexican* OR micronesia* OR moldova* OR mongolia* OR montenegr* OR morocc* OR mozambi* OR myanmar* OR namibia* OR nepal* OR nicaragua* OR niger OR nigerien* OR nigeria* OR macedonia* OR pakistan* OR paragua* OR peru* OR philippines OR filipino* OR russia* OR rwanda* OR são tomé and principe OR senegal* OR serbia* OR sierra leone* OR solomon island* OR somalia* OR south africa* OR sudan* OR sri lank* OR st lucia* OR saint lucia* OR st. vincent* OR saint vincent* OR grenadines OR surinam* OR syria* OR tajikistan* OR tanzania* OR thailand OR thai OR thais OR timor* OR togo OR togolese* OR tonga* OR tunisia* OR turkey OR turks OR turkish OR turkmenistan* OR tuvalu* OR uganda* OR ukrain* OR uzbekistan* OR vanuatu* OR venezuela* OR vietnam* OR viet nam* OR west bank OR gaza OR gazan* OR yemen* OR zambia* OR zimbabwe*)) OR

(AB (“developing countr*” OR “developing nation*” OR “low-income countr*” OR “low income countr*” OR “low-income nation*” OR “low income nation*” OR “third world countr*” OR “third-world countr*” OR “third world nation*” OR “third-world nation*” OR “under-developed countr*” OR “under developed countr*” OR “under developed nation*” OR “under-developed nation*” OR “middle-income countr*” OR “middle-income nation*” OR “middle-income nation*” OR “middle income countr*” OR underserved area* OR developing econom* OR resource poor OR resource limit* OR limited resource* OR limiting resource* OR low resource OR resource constrain* OR constrained resource* OR middle income countr* OR middle income nation* OR low income countr* OR low income nation* OR poor countr* OR poor nation* OR lmic OR lmics OR africa OR asia OR south america* OR latin america* OR central america* OR afghanistan* OR albania* OR algeria* OR samoa* OR angola* OR argentina* OR armenia* OR azerbaijan* OR bangladesh* OR belarus* OR belize* OR benin* OR bhutan* OR bolivia* OR bosnia* OR herzegovina* OR botswana* OR brazil* OR bulgaria* OR burkin* OR burundi* OR cabo verde* OR cape verde* OR cambodia* OR cameroon* OR central africa* OR chad* OR china OR chinese OR colombia* OR comoros OR comorian* OR congo* OR costa rica* OR côte d'ivoire OR ivorian* OR ivory coast OR cuba* OR djibouti* OR dominica* OR ecuador* OR egypt* OR el salvador OR salvadoran* OR guinea* OR eritrea* OR eswatini* OR swaziland* OR ethiopia* OR fiji* OR gabon* OR gambia* OR republic of georgia OR ghana* OR grenada* OR guatemala* OR guyana* OR haiti* OR honduras* OR india* OR indonesia* OR iran* OR iraq* OR jamaica* OR jordan* OR kazakhstan* OR kenya* OR kiribati* OR democratic people's republic of korea OR north korea* OR kosovo OR kosovar* OR kyrgyz* OR lao OR laos OR laotian* OR lebanon* OR lesotho OR liberia* OR libya* OR madagascar* OR malawi* OR malaysia* OR maldives* OR mali OR malian OR malians OR marshall island* OR mauritania* OR mexico OR mexican* OR micronesia* OR moldova* OR mongolia* OR montenegr* OR morocc* OR mozambi* OR myanmar* OR namibia* OR nepal* OR nicaragua* OR niger OR nigerien* OR nigeria* OR macedonia* OR pakistan* OR paragua* OR peru* OR philippines OR filipino* OR russia* OR rwanda* OR são tomé and principe OR senegal* OR serbia* OR sierra leone* OR solomon island* OR somalia* OR south africa* OR sudan* OR sri lank* OR st lucia* OR saint lucia* OR st. vincent* OR saint vincent* OR grenadines OR surinam* OR syria* OR tajikistan* OR tanzania* OR thailand OR thai OR thais OR timor* OR togo OR togolese* OR tonga* OR tunisia* OR turkey OR turks OR turkish OR turkmenistan* OR tuvalu* OR uganda* OR ukrain* OR uzbekistan* OR vanuatu* OR venezuela* OR vietnam* OR viet nam* OR west bank OR gaza OR gazan* OR yemen* OR zambia* OR zimbabwe*)) OR

(SU (“developing countr*” OR “developing nation*” OR “low-income countr*” OR “low income countr*” OR “low-income nation*” OR “low income nation*” OR “third world countr*” OR “third-world countr*” OR “third world nation*” OR “third-world nation*” OR “under-developed countr*” OR “under developed countr*” OR “under developed nation*” OR “under-developed nation*” OR “middle-income countr*” OR “middle-income nation*” OR “middle-income nation*” OR “middle income countr*” OR underserved area* OR developing econom* OR resource poor OR resource limit* OR limited resource* OR limiting resource* OR low resource OR resource constrain* OR constrained resource* OR middle income countr* OR middle income nation* OR low income countr* OR low income nation* OR poor countr* OR poor nation* OR lmic OR lmics OR africa OR asia OR south america* OR latin america* OR central america* OR afghanistan* OR albania* OR algeria* OR samoa* OR angola* OR argentina* OR armenia* OR azerbaijan* OR bangladesh* OR belarus* OR belize* OR benin* OR bhutan* OR bolivia* OR bosnia* OR herzegovina* OR botswana* OR brazil* OR bulgaria* OR burkin* OR burundi* OR cabo verde* OR cape verde* OR cambodia* OR cameroon* OR central africa* OR chad* OR china OR chinese OR colombia* OR comoros OR comorian* OR congo* OR costa rica* OR côte d'ivoire OR ivorian* OR ivory coast OR cuba* OR djibouti* OR dominica* OR ecuador* OR egypt* OR el salvador OR salvadoran* OR guinea* OR eritrea* OR eswatini* OR swaziland* OR ethiopia* OR fiji* OR gabon* OR gambia* OR republic of georgia OR ghana* OR grenada* OR guatemala* OR guyana* OR haiti* OR honduras* OR india* OR indonesia* OR iran* OR iraq* OR jamaica* OR jordan* OR kazakhstan* OR kenya* OR kiribati* OR democratic people's republic of korea OR north korea* OR kosovo OR kosovar* OR kyrgyz* OR lao OR laos OR laotian* OR lebanon* OR lesotho OR liberia* OR libya* OR madagascar* OR malawi* OR malaysia* OR maldives* OR mali OR malian OR malians OR marshall island* OR mauritania* OR mexico OR mexican* OR micronesia* OR moldova* OR mongolia* OR montenegr* OR morocc* OR mozambi* OR myanmar* OR namibia* OR nepal* OR nicaragua* OR niger OR nigerien* OR nigeria* OR macedonia* OR pakistan* OR paragua* OR peru* OR philippines OR filipino* OR russia* OR rwanda* OR são tomé and principe OR senegal* OR serbia* OR sierra leone* OR solomon island* OR somalia* OR south africa* OR sudan* OR sri lank* OR st lucia* OR saint lucia* OR st. vincent* OR saint vincent* OR grenadines OR surinam* OR syria* OR tajikistan* OR tanzania* OR thailand OR thai OR thais OR timor* OR togo OR togolese* OR tonga* OR tunisia* OR turkey OR turks OR turkish OR turkmenistan* OR tuvalu* OR uganda* OR ukrain* OR uzbekistan* OR vanuatu* OR venezuela* OR vietnam* OR viet nam* OR west bank OR gaza OR gazan* OR yemen* OR zambia* OR zimbabwe*)))

**E. WHO Regional Database (Global Index Medicus)**

tw:((tw:(depress*)) AND (tw:(matern* OR postpartum OR post-partum OR post partum OR antenatal OR ante-natal OR ante natal OR pregn*)) AND (tw:(child development OR infant development OR child growth OR infant growth OR gestational size OR birth weight OR growth outcome OR fetal growth OR stunt* OR wasting OR weight for age OR bmi for age OR weight for height OR weight for age OR length for age OR weight for length)))

**F. LILACS**

(((Depression, Postpartum) OR (Depresión Posparto) OR (maternal depression) OR (Depresión Postnatal) OR (perinatal depression) OR (Depresión Puerperal) OR (postnatal depression) OR (Depresión Postparto) OR (prenatal depression) OR (antenatal depression)) OR ((Depression OR depression$ OR Depresión OR (Síntomas Depresivos)) AND (mother$ OR madre$ OR (pregnant women) OR pregnancy OR (postpartum period) OR postpartum$ OR post-partum$ OR (post partum)$ OR (post natal)$ OR post-natal$ OR postnatal$ OR (peripartum period) OR (peri partum)$ OR peri-partum$ OR peripartum$ OR pre natal$ OR pre-natal$ OR prenatal$ OR (ante natal)$ OR ante-natal$ OR antenatal$ OR (Mujeres Embarazadas) OR (embarazadas) OR embarazo$ OR (mujer embarazada) OR gestante$ OR (Periodo Posparto) OR (periodo postparto) OR (periodo de posparto) OR puerperio OR (periodo periparto) OR postparto$ OR posparto$ OR (peri parto)$ OR periparto$ OR prenatal$)))

AND

((child nutrition disorders) OR (Trastornos de la Nutrición del Niño) OR (Desnutrición Infantil) OR (infant nutrition disorders) OR (desnutrición crónica) OR (wasting syndrome) OR Emaciación OR (Desnutrición Aguda Severa) OR ((child$ OR infant$ OR (children under 5) OR (children under five)) AND (malnutrition$ OR undernutrition$ OR overnutrition$)) OR ((niño$ OR infante$) AND (desnutrición$ OR sobrenutrición$ OR crecimiento)) OR (child nutrition) OR (nutrición infantil) OR (infant nutrition) OR (pediatric obesity) OR (obesidad pediátrica) OR Hipernutrición OR (growth, stunted) OR (stunted growth) OR (stunting))

**G. CINAHL**

((TI ((postpartum OR “post partum” OR perinatal OR “peri natal” OR antenatal OR pre-natal OR prenatal OR “pre natal” OR maternal) AND (depression OR depressive OR depressed))) OR (AB ((postpartum OR “post partum” OR perinatal OR “peri natal” OR antenatal OR pre-natal OR prenatal OR “pre natal” OR maternal) AND (depression OR depressive OR depressed))) OR (SU ((postpartum OR “post partum” OR perinatal OR “peri natal” OR antenatal OR pre-natal OR prenatal OR “pre natal” OR maternal) AND (depression OR depressive OR depressed))) OR (MM (“Depression, Postpartum” OR (“Depression” AND “Pregnancy”) OR “Pregnancy Complications, Psychiatric”)))

AND

((TI (“growth outcomes” OR “growth complications” OR “underweight” OR “under weight” OR “overweight” OR “over weight” OR “stunting” OR “malnutrition” OR “nutritional defici*” OR “neonatal outcomes” OR “growth delay” OR “wasting”)) OR (AB (“growth outcomes” OR “growth complications” OR “underweight” OR “under weight” OR “overweight” OR “over weight” OR “stunting” OR “malnutrition” OR “nutritional defici*” OR “neonatal outcomes” OR “growth delay” OR “wasting”)) OR (SU (“growth outcomes” OR “growth complications” OR “underweight” OR “under weight” OR “overweight” OR “over weight” OR “stunting” OR “malnutrition” OR “nutritional defici*” OR “neonatal outcomes” OR “growth delay” OR “wasting”)) OR (MM(“Growth” OR “Body Weight” OR “Malnutrition” OR “Wasting”)))

AND

(( TI(“developing countr*” OR “developing nation*” OR “low-income countr*” OR “low income countr*” OR “low-income nation*” OR “low income nation*” OR “third world countr*” OR “third-world countr*” OR “third world nation*” OR “third-world nation*” OR “under-developed countr*” OR “under developed countr*” OR “under developed nation*” OR “under-developed nation*” OR “middle-income countr*” OR “middle-income nation*” OR “middle-income nation*” OR “middle income countr*” OR underserved area* OR developing econom* OR resource poor OR resource limit* OR limited resource* OR limiting resource* OR low resource OR resource constrain* OR constrained resource* OR middle income countr* OR middle income nation* OR low income countr* OR low income nation* OR poor countr* OR poor nation* OR lmic OR lmics OR africa OR asia OR south america* OR latin america* OR central america* OR afghanistan* OR albania* OR algeria* OR samoa* OR angola* OR argentina* OR armenia* OR azerbaijan* OR bangladesh* OR belarus* OR belize* OR benin* OR bhutan* OR bolivia* OR bosnia* OR herzegovina* OR botswana* OR brazil* OR bulgaria* OR burkin* OR burundi* OR cabo verde* OR cape verde* OR cambodia* OR cameroon* OR central africa* OR chad* OR china OR chinese OR colombia* OR comoros OR comorian* OR congo* OR costa rica* OR côte d'ivoire OR ivorian* OR ivory coast OR cuba* OR djibouti* OR dominica* OR ecuador* OR egypt* OR el salvador OR salvadoran* OR guinea* OR eritrea* OR eswatini* OR swaziland* OR ethiopia* OR fiji* OR gabon* OR gambia* OR republic of georgia OR ghana* OR grenada* OR guatemala* OR guyana* OR haiti* OR honduras* OR india* OR indonesia* OR iran* OR iraq* OR jamaica* OR jordan* OR kazakhstan* OR kenya* OR kiribati* OR democratic people's republic of korea OR north korea* OR kosovo OR kosovar* OR kyrgyz* OR lao OR laos OR laotian* OR lebanon* OR lesotho OR liberia* OR libya* OR madagascar* OR malawi* OR malaysia* OR maldives* OR mali OR malian OR malians OR marshall island* OR mauritania* OR mexico OR mexican* OR micronesia* OR moldova* OR mongolia* OR montenegr* OR morocc* OR mozambi* OR myanmar* OR namibia* OR nepal* OR nicaragua* OR niger OR nigerien* OR nigeria* OR macedonia* OR pakistan* OR paragua* OR peru* OR philippines OR filipino* OR russia* OR rwanda* OR são tomé and principe OR senegal* OR serbia* OR sierra leone* OR solomon island* OR somalia* OR south africa* OR sudan* OR sri lank* OR st lucia* OR saint lucia* OR st. vincent* OR saint vincent* OR grenadines OR surinam* OR syria* OR tajikistan* OR tanzania* OR thailand OR thai OR thais OR timor* OR togo OR togolese* OR tonga* OR tunisia* OR turkey OR turks OR turkish OR turkmenistan* OR tuvalu* OR uganda* OR ukrain* OR uzbekistan* OR vanuatu* OR venezuela* OR vietnam* OR viet nam* OR west bank OR gaza OR gazan* OR yemen* OR zambia* OR zimbabwe*)) OR

(AB (“developing countr*” OR “developing nation*” OR “low-income countr*” OR “low income countr*” OR “low-income nation*” OR “low income nation*” OR “third world countr*” OR “third-world countr*” OR “third world nation*” OR “third-world nation*” OR “under-developed countr*” OR “under developed countr*” OR “under developed nation*” OR “under-developed nation*” OR “middle-income countr*” OR “middle-income nation*” OR “middle-income nation*” OR “middle income countr*” OR underserved area* OR developing econom* OR resource poor OR resource limit* OR limited resource* OR limiting resource* OR low resource OR resource constrain* OR constrained resource* OR middle income countr* OR middle income nation* OR low income countr* OR low income nation* OR poor countr* OR poor nation* OR lmic OR lmics OR africa OR asia OR south america* OR latin america* OR central america* OR afghanistan* OR albania* OR algeria* OR samoa* OR angola* OR argentina* OR armenia* OR azerbaijan* OR bangladesh* OR belarus* OR belize* OR benin* OR bhutan* OR bolivia* OR bosnia* OR herzegovina* OR botswana* OR brazil* OR bulgaria* OR burkin* OR burundi* OR cabo verde* OR cape verde* OR cambodia* OR cameroon* OR central africa* OR chad* OR china OR chinese OR colombia* OR comoros OR comorian* OR congo* OR costa rica* OR côte d'ivoire OR ivorian* OR ivory coast OR cuba* OR djibouti* OR dominica* OR ecuador* OR egypt* OR el salvador OR salvadoran* OR guinea* OR eritrea* OR eswatini* OR swaziland* OR ethiopia* OR fiji* OR gabon* OR gambia* OR republic of georgia OR ghana* OR grenada* OR guatemala* OR guyana* OR haiti* OR honduras* OR india* OR indonesia* OR iran* OR iraq* OR jamaica* OR jordan* OR kazakhstan* OR kenya* OR kiribati* OR democratic people's republic of korea OR north korea* OR kosovo OR kosovar* OR kyrgyz* OR lao OR laos OR laotian* OR lebanon* OR lesotho OR liberia* OR libya* OR madagascar* OR malawi* OR malaysia* OR maldives* OR mali OR malian OR malians OR marshall island* OR mauritania* OR mexico OR mexican* OR micronesia* OR moldova* OR mongolia* OR montenegr* OR morocc* OR mozambi* OR myanmar* OR namibia* OR nepal* OR nicaragua* OR niger OR nigerien* OR nigeria* OR macedonia* OR pakistan* OR paragua* OR peru* OR philippines OR filipino* OR russia* OR rwanda* OR são tomé and principe OR senegal* OR serbia* OR sierra leone* OR solomon island* OR somalia* OR south africa* OR sudan* OR sri lank* OR st lucia* OR saint lucia* OR st. vincent* OR saint vincent* OR grenadines OR surinam* OR syria* OR tajikistan* OR tanzania* OR thailand OR thai OR thais OR timor* OR togo OR togolese* OR tonga* OR tunisia* OR turkey OR turks OR turkish OR turkmenistan* OR tuvalu* OR uganda* OR ukrain* OR uzbekistan* OR vanuatu* OR venezuela* OR vietnam* OR viet nam* OR west bank OR gaza OR gazan* OR yemen* OR zambia* OR zimbabwe*)) OR

(SU (“developing countr*” OR “developing nation*” OR “low-income countr*” OR “low income countr*” OR “low-income nation*” OR “low income nation*” OR “third world countr*” OR “third-world countr*” OR “third world nation*” OR “third-world nation*” OR “under-developed countr*” OR “under developed countr*” OR “under developed nation*” OR “under-developed nation*” OR “middle-income countr*” OR “middle-income nation*” OR “middle-income nation*” OR “middle income countr*” OR underserved area* OR developing econom* OR resource poor OR resource limit* OR limited resource* OR limiting resource* OR low resource OR resource constrain* OR constrained resource* OR middle income countr* OR middle income nation* OR low income countr* OR low income nation* OR poor countr* OR poor nation* OR lmic OR lmics OR africa OR asia OR south america* OR latin america* OR central america* OR afghanistan* OR albania* OR algeria* OR samoa* OR angola* OR argentina* OR armenia* OR azerbaijan* OR bangladesh* OR belarus* OR belize* OR benin* OR bhutan* OR bolivia* OR bosnia* OR herzegovina* OR botswana* OR brazil* OR bulgaria* OR burkin* OR burundi* OR cabo verde* OR cape verde* OR cambodia* OR cameroon* OR central africa* OR chad* OR china OR chinese OR colombia* OR comoros OR comorian* OR congo* OR costa rica* OR côte d'ivoire OR ivorian* OR ivory coast OR cuba* OR djibouti* OR dominica* OR ecuador* OR egypt* OR el salvador OR salvadoran* OR guinea* OR eritrea* OR eswatini* OR swaziland* OR ethiopia* OR fiji* OR gabon* OR gambia* OR republic of georgia OR ghana* OR grenada* OR guatemala* OR guyana* OR haiti* OR honduras* OR india* OR indonesia* OR iran* OR iraq* OR jamaica* OR jordan* OR kazakhstan* OR kenya* OR kiribati* OR democratic people's republic of korea OR north korea* OR kosovo OR kosovar* OR kyrgyz* OR lao OR laos OR laotian* OR lebanon* OR lesotho OR liberia* OR libya* OR madagascar* OR malawi* OR malaysia* OR maldives* OR mali OR malian OR malians OR marshall island* OR mauritania* OR mexico OR mexican* OR micronesia* OR moldova* OR mongolia* OR montenegr* OR morocc* OR mozambi* OR myanmar* OR namibia* OR nepal* OR nicaragua* OR niger OR nigerien* OR nigeria* OR macedonia* OR pakistan* OR paragua* OR peru* OR philippines OR filipino* OR russia* OR rwanda* OR são tomé and principe OR senegal* OR serbia* OR sierra leone* OR solomon island* OR somalia* OR south africa* OR sudan* OR sri lank* OR st lucia* OR saint lucia* OR st. vincent* OR saint vincent* OR grenadines OR surinam* OR syria* OR tajikistan* OR tanzania* OR thailand OR thai OR thais OR timor* OR togo OR togolese* OR tonga* OR tunisia* OR turkey OR turks OR turkish OR turkmenistan* OR tuvalu* OR uganda* OR ukrain* OR uzbekistan* OR vanuatu* OR venezuela* OR vietnam* OR viet nam* OR west bank OR gaza OR gazan* OR yemen* OR zambia* OR zimbabwe*)) OR

(MM(“Low and Middle Income Countries” OR “Developing Countries”)))

1. **PsycINFO**

('perinatal depression' OR ('pregnant woman' AND 'depression') OR ((postpartum OR 'post partum' OR perinatal OR pre-natal OR ‘pre natal’ OR postnatal OR prenatal OR 'peri natal' OR 'post natal' OR maternal) AND (depression OR depressive)))

AND ('stunting' OR 'stunting' OR 'failure to thrive' OR 'failure to thrive' OR 'failure to thrive' OR 'short stature' OR 'short stature' OR 'short stature' OR 'short body stature'/exp OR 'short body stature' OR 'short body stature' OR 'small stature' OR 'small stature' OR 'small stature' OR 'malnutrition inflammation atherosclerosis syndrome' OR OR 'malnutrition' OR 'protein deficiency' OR 'protein deficiency' OR 'protein deficiency' OR 'nutritional disorder' OR 'nutritional disorder' OR 'nutritional disorder' OR 'infant nutrition disorder' OR 'nutrition disorder, infant' OR 'nutrition disorders, infant' OR 'infant overnutrition' OR 'overnutrition, infant' OR 'infant malnutrition' OR 'malnutrition in infant' OR 'malnutrition in infants' OR 'malnutrition, infant' OR 'infantile malnutrition' OR 'malnutrition, infantile' OR 'deficient nutrition' OR 'deficient nutrition' OR 'deficient nutrition' OR 'malnourishment' OR 'malnourishment' OR 'malnourishment' OR 'severe acute malnutrition' OR 'severe acute malnutrition' OR 'severe acute malnutrition' OR 'underfeeding' OR 'underfeeding' OR 'underfeeding' OR 'undernourishment' OR 'undernourishment' OR 'undernourishment' OR 'deficiency, protein' OR 'deficiency, protein' OR 'deficiency, protein' OR 'dietary protein deficiency' OR 'dietary protein deficiency' OR 'dietary protein deficiency' OR 'alimentary deficiency’ OR 'alimentary deficiency' OR 'alimentary deficiency' OR 'defective diet' OR 'defective diet' OR 'defective diet' OR 'deficiency disease' OR 'deficiency disease' OR 'deficiency disease' OR 'deficiency diseases' OR 'deficiency diseases' OR 'deficiency diseases' OR 'deficiency, nutritional' OR 'deficiency, nutritional' OR 'deficiency, nutritional' OR 'deficient diet' OR 'deficient diet' OR 'deficient diet' OR 'diet deficiency' OR 'diet deficiency' OR 'diet deficiency' OR 'diet insufficiency' OR 'diet insufficiency' OR 'diet insufficiency' OR 'diet, defective' OR 'diet, defective' OR 'diet, defective' OR 'dietary deficiency' OR 'dietary deficiency' OR 'dietary deficiency' OR 'dietary insufficiency' OR 'dietary insufficiency' OR 'dietary insufficiency' OR 'food deficiency' OR 'food deficiency' OR 'micronutrient deficiency' OR 'micronutrient deficiency' OR 'micronutrient deficiency' OR 'micronutrient insufficiency' OR 'micronutrient insufficiency' OR 'micronutrient insufficiency' OR 'nutrient deficiency' OR 'nutrient deficiency' OR 'nutrient deficiency' OR 'nutritional deficiency' OR 'nutritional deficiency' OR 'nutritional deficiency' OR 'nutrition deficiency' OR 'nutrition deficiency' OR 'nutrition deficiency' OR 'nutritional deficit' OR 'nutritional deficit' OR 'nutritional deficit' OR 'nutritive deficiency' OR 'nutritive deficiency' OR 'nutritive deficiency' OR 'overweight' OR 'obesity’ OR 'obesity' OR 'wasting' OR ‘adipos*’ OR ‘body weight’ OR ‘fat overload syndrome’ OR ‘nutritional obesity’)

AND(“developing countr*” OR “developing nation*” OR “low-income countr*” OR “low income countr*” OR “low-income nation*” OR “low income nation*” OR “third world countr*” OR “third-world countr*” OR “third world nation*” OR “third-world nation*” OR “under-developed countr*” OR “under developed countr*” OR “under developed nation*” OR “under-developed nation*” OR “middle-income countr*” OR “middle-income nation*” OR “middle-income nation*” OR “middle income countr*” OR underserved area* OR developing econom* OR resource poor OR resource limit* OR limited resource* OR limiting resource* OR low resource OR resource constrain* OR constrained resource* OR middle income countr* OR middle income nation* OR low income countr* OR low income nation* OR poor countr* OR poor nation* OR lmic OR lmics OR africa OR asia OR south america* OR latin america* OR central america* OR afghanistan* OR albania* OR algeria* OR samoa* OR angola* OR argentina* OR armenia* OR azerbaijan* OR bangladesh* OR belarus* OR belize* OR benin* OR bhutan* OR bolivia* OR bosnia* OR herzegovina* OR botswana* OR brazil* OR bulgaria* OR burkin* OR burundi* OR cabo verde* OR cape verde* OR cambodia* OR cameroon* OR central africa* OR chad* OR china OR chinese OR colombia* OR comoros OR comorian* OR congo* OR costa rica* OR côte d'ivoire OR ivorian* OR ivory coast OR cuba* OR djibouti* OR dominica* OR ecuador* OR egypt* OR el salvador OR salvadoran* OR guinea* OR eritrea* OR eswatini* OR swaziland* OR ethiopia* OR fiji* OR gabon* OR gambia* OR republic of georgia OR ghana* OR grenada* OR guatemala* OR guyana* OR haiti* OR honduras* OR india* OR indonesia* OR iran* OR iraq* OR jamaica* OR jordan* OR kazakhstan* OR kenya* OR kiribati* OR democratic people's republic of korea OR north korea* OR kosovo OR kosovar* OR kyrgyz* OR lao OR laos OR laotian* OR lebanon* OR lesotho OR liberia* OR libya* OR madagascar* OR malawi* OR malaysia* OR maldives* OR mali OR malian OR malians OR marshall island* OR mauritania* OR mexico OR mexican* OR micronesia* OR moldova* OR mongolia* OR montenegr* OR morocc* OR mozambi* OR myanmar* OR namibia* OR nepal* OR nicaragua* OR niger OR nigerien* OR nigeria* OR macedonia* OR pakistan* OR paragua* OR peru* OR philippines OR filipino* OR russia* OR rwanda* OR são tomé and principe OR senegal* OR serbia* OR sierra leone* OR solomon island* OR somalia* OR south africa* OR sudan* OR sri lank* OR st lucia* OR saint lucia* OR st. vincent* OR saint vincent* OR grenadines OR surinam* OR syria* OR tajikistan* OR tanzania* OR thailand OR thai OR thais OR timor* OR togo OR togolese* OR tonga* OR tunisia* OR turkey OR turks OR turkish OR turkmenistan* OR tuvalu* OR uganda* OR ukrain* OR uzbekistan* OR vanuatu* OR venezuela* OR vietnam* OR viet nam* OR west bank OR gaza OR gazan* OR yemen* OR zambia* OR zimbabwe*)) OR

(AB (“developing countr*” OR “developing nation*” OR “low-income countr*” OR “low income countr*” OR “low-income nation*” OR “low income nation*” OR “third world countr*” OR “third-world countr*” OR “third world nation*” OR “third-world nation*” OR “under-developed countr*” OR “under developed countr*” OR “under developed nation*” OR “under-developed nation*” OR “middle-income countr*” OR “middle-income nation*” OR “middle-income nation*” OR “middle income countr*” OR underserved area* OR developing econom* OR resource poor OR resource limit* OR limited resource* OR limiting resource* OR low resource OR resource constrain* OR constrained resource* OR middle income countr* OR middle income nation* OR low income countr* OR low income nation* OR poor countr* OR poor nation* OR lmic OR lmics OR africa OR asia OR south america* OR latin america* OR central america* OR afghanistan* OR albania* OR algeria* OR samoa* OR angola* OR argentina* OR armenia* OR azerbaijan* OR bangladesh* OR belarus* OR belize* OR benin* OR bhutan* OR bolivia* OR bosnia* OR herzegovina* OR botswana* OR brazil* OR bulgaria* OR burkin* OR burundi* OR cabo verde* OR cape verde* OR cambodia* OR cameroon* OR central africa* OR chad* OR china OR chinese OR colombia* OR comoros OR comorian* OR congo* OR costa rica* OR côte d'ivoire OR ivorian* OR ivory coast OR cuba* OR djibouti* OR dominica* OR ecuador* OR egypt* OR el salvador OR salvadoran* OR guinea* OR eritrea* OR eswatini* OR swaziland* OR ethiopia* OR fiji* OR gabon* OR gambia* OR republic of georgia OR ghana* OR grenada* OR guatemala* OR guyana* OR haiti* OR honduras* OR india* OR indonesia* OR iran* OR iraq* OR jamaica* OR jordan* OR kazakhstan* OR kenya* OR kiribati* OR democratic people's republic of korea OR north korea* OR kosovo OR kosovar* OR kyrgyz* OR lao OR laos OR laotian* OR lebanon* OR lesotho OR liberia* OR libya* OR madagascar* OR malawi* OR malaysia* OR maldives* OR mali OR malian OR malians OR marshall island* OR mauritania* OR mexico OR mexican* OR micronesia* OR moldova* OR mongolia* OR montenegr* OR morocc* OR mozambi* OR myanmar* OR namibia* OR nepal* OR nicaragua* OR niger OR nigerien* OR nigeria* OR macedonia* OR pakistan* OR paragua* OR peru* OR philippines OR filipino* OR russia* OR rwanda* OR são tomé and principe OR senegal* OR serbia* OR sierra leone* OR solomon island* OR somalia* OR south africa* OR sudan* OR sri lank* OR st lucia* OR saint lucia* OR st. vincent* OR saint vincent* OR grenadines OR surinam* OR syria* OR tajikistan* OR tanzania* OR thailand OR thai OR thais OR timor* OR togo OR togolese* OR tonga* OR tunisia* OR turkey OR turks OR turkish OR turkmenistan* OR tuvalu* OR uganda* OR ukrain* OR uzbekistan* OR vanuatu* OR venezuela* OR vietnam* OR viet nam* OR west bank OR gaza OR gazan* OR yemen* OR zambia* OR zimbabwe*)) OR

(SU (“developing countr*” OR “developing nation*” OR “low-income countr*” OR “low income countr*” OR “low-income nation*” OR “low income nation*” OR “third world countr*” OR “third-world countr*” OR “third world nation*” OR “third-world nation*” OR “under-developed countr*” OR “under developed countr*” OR “under developed nation*” OR “under-developed nation*” OR “middle-income countr*” OR “middle-income nation*” OR “middle-income nation*” OR “middle income countr*” OR underserved area* OR developing econom* OR resource poor OR resource limit* OR limited resource* OR limiting resource* OR low resource OR resource constrain* OR constrained resource* OR middle income countr* OR middle income nation* OR low income countr* OR low income nation* OR poor countr* OR poor nation* OR lmic OR lmics OR africa OR asia OR south america* OR latin america* OR central america* OR afghanistan* OR albania* OR algeria* OR samoa* OR angola* OR argentina* OR armenia* OR azerbaijan* OR bangladesh* OR belarus* OR belize* OR benin* OR bhutan* OR bolivia* OR bosnia* OR herzegovina* OR botswana* OR brazil* OR bulgaria* OR burkin* OR burundi* OR cabo verde* OR cape verde* OR cambodia* OR cameroon* OR central africa* OR chad* OR china OR chinese OR colombia* OR comoros OR comorian* OR congo* OR costa rica* OR côte d'ivoire OR ivorian* OR ivory coast OR cuba* OR djibouti* OR dominica* OR ecuador* OR egypt* OR el salvador OR salvadoran* OR guinea* OR eritrea* OR eswatini* OR swaziland* OR ethiopia* OR fiji* OR gabon* OR gambia* OR republic of georgia OR ghana* OR grenada* OR guatemala* OR guyana* OR haiti* OR honduras* OR india* OR indonesia* OR iran* OR iraq* OR jamaica* OR jordan* OR kazakhstan* OR kenya* OR kiribati* OR democratic people's republic of korea OR north korea* OR kosovo OR kosovar* OR kyrgyz* OR lao OR laos OR laotian* OR lebanon* OR lesotho OR liberia* OR libya* OR madagascar* OR malawi* OR malaysia* OR maldives* OR mali OR malian OR malians OR marshall island* OR mauritania* OR mexico OR mexican* OR micronesia* OR moldova* OR mongolia* OR montenegr* OR morocc* OR mozambi* OR myanmar* OR namibia* OR nepal* OR nicaragua* OR niger OR nigerien* OR nigeria* OR macedonia* OR pakistan* OR paragua* OR peru* OR philippines OR filipino* OR russia* OR rwanda* OR são tomé and principe OR senegal* OR serbia* OR sierra leone* OR solomon island* OR somalia* OR south africa* OR sudan* OR sri lank* OR st lucia* OR saint lucia* OR st. vincent* OR saint vincent* OR grenadines OR surinam* OR syria* OR tajikistan* OR tanzania* OR thailand OR thai OR thais OR timor* OR togo OR togolese* OR tonga* OR tunisia* OR turkey OR turks OR turkish OR turkmenistan* OR tuvalu* OR uganda* OR ukrain* OR uzbekistan* OR vanuatu* OR venezuela* OR vietnam* OR viet nam* OR west bank OR gaza OR gazan* OR yemen* OR zambia* OR zimbabwe*)) OR

(MM(“Low and Middle Income Countries” OR “Developing Countries”)

1. **Google Scholar (the first 100 articles)**

(perinatal depression OR postpartum OR mother* OR antenatal OR peripartum OR antepartum) AND (growth OR height OR weight OR BMI OR malnutrition OR nutritional OR obesity OR overweight) AND (low income OR africa OR asia OR america OR middle income OR LMICs)
