# Supplementary figures and images for: Perinatal depression and adverse child growth outcomes in low-income and middle-income countries (LMICs): A systematic review and meta-analysis
Source: PLOS Glob Public Health. 2024 Oct 28;4(10):e0003586. doi: 10.1371/journal.pgph.0003586 (PMC11516009; doi:10.1371/journal.pgph.0003586)

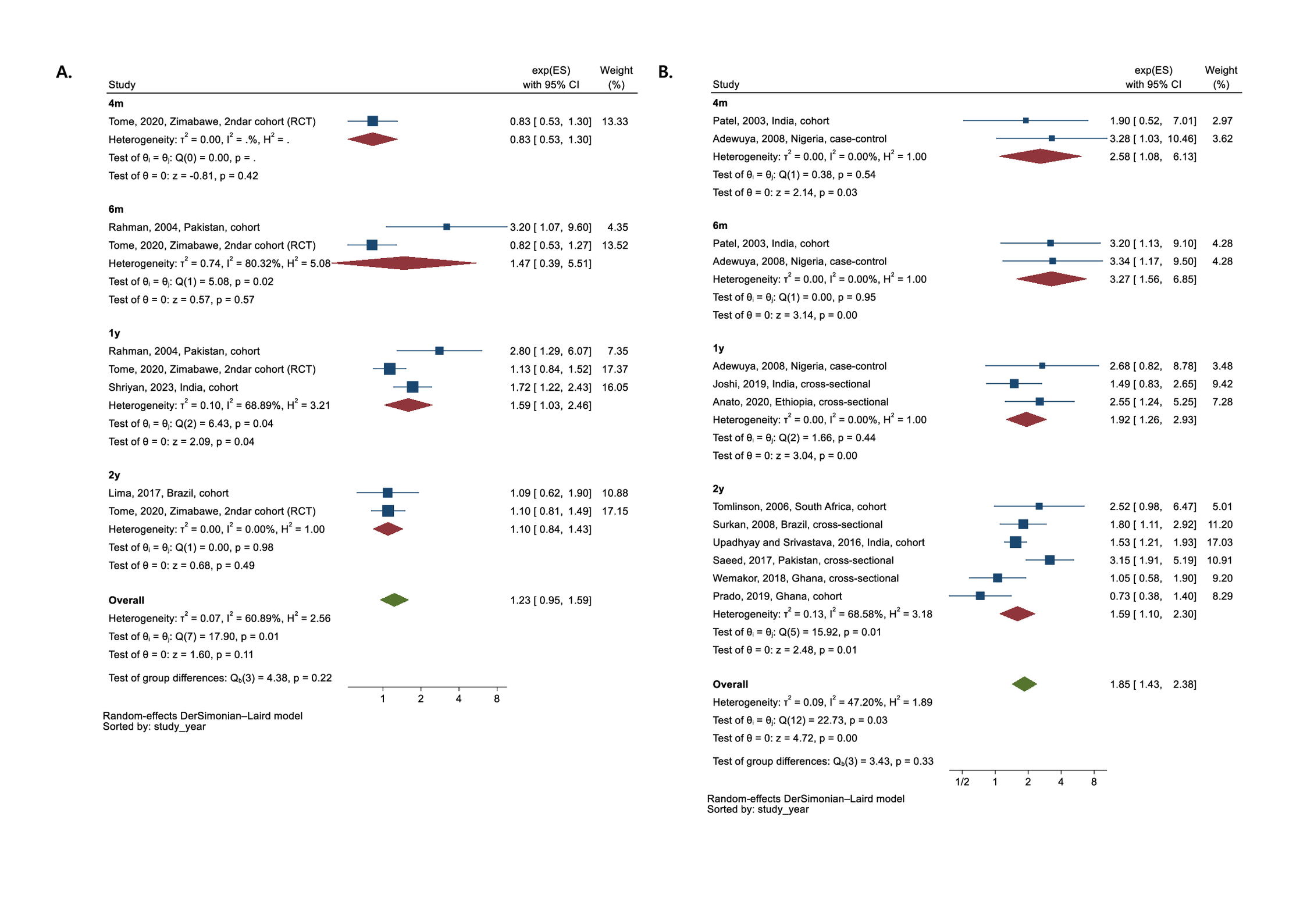

Supplement: S1 Fig — A. The odds ratio between antepartum depression and stunting according to the timepoint of outcome measurement. B. The odds ratio between postpartum depression and stunting according to the timepoint of outcome measurement. (TIF) [file pgph.0003586.s007.tif]

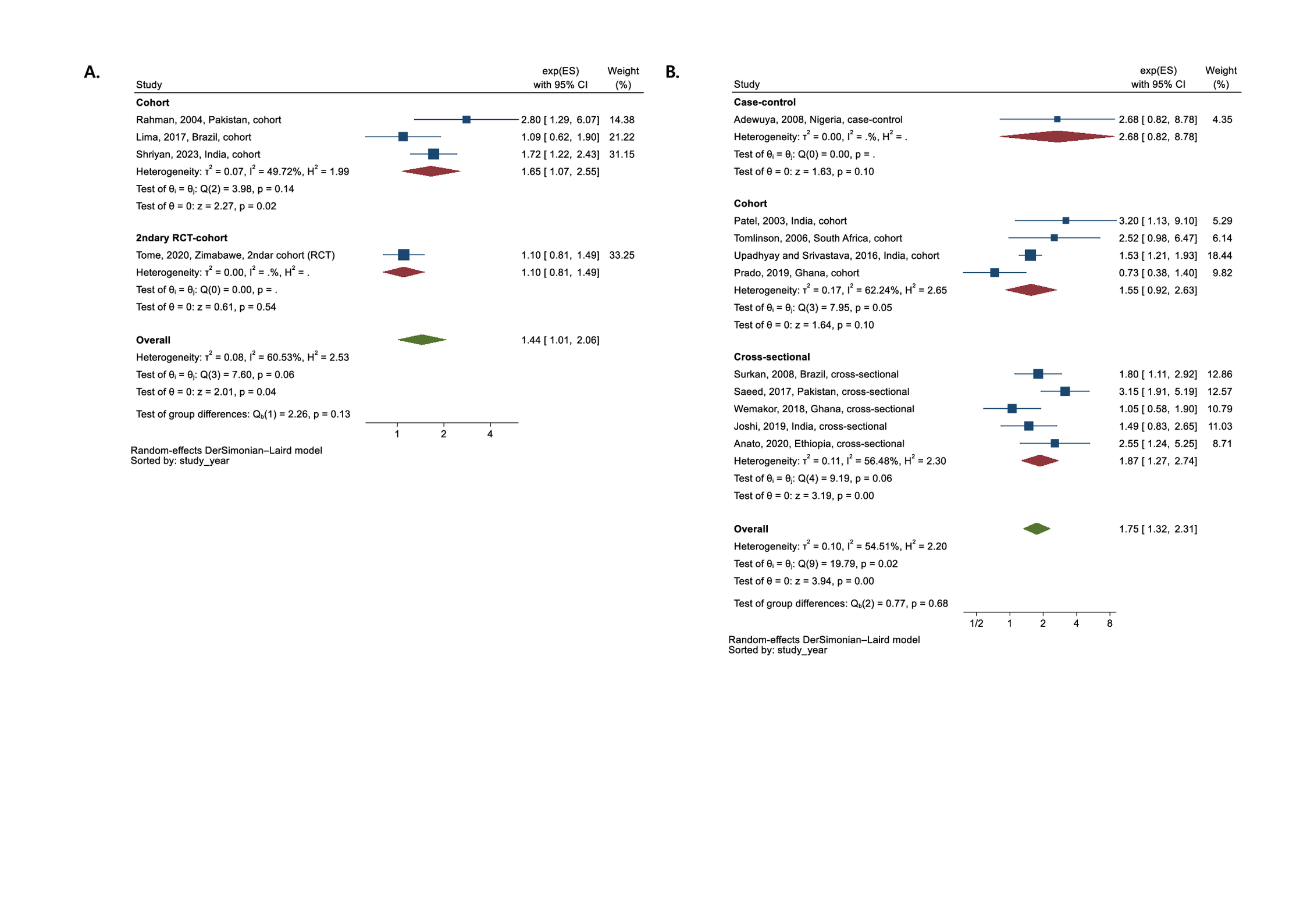

Supplement: S2 Fig — A. The odds ratio between antepartum depression and stunting according to the study design (n = 4). B. The odds ratio between postpartum depression and stunting (n = 10). (TIF) [file pgph.0003586.s008.tif]

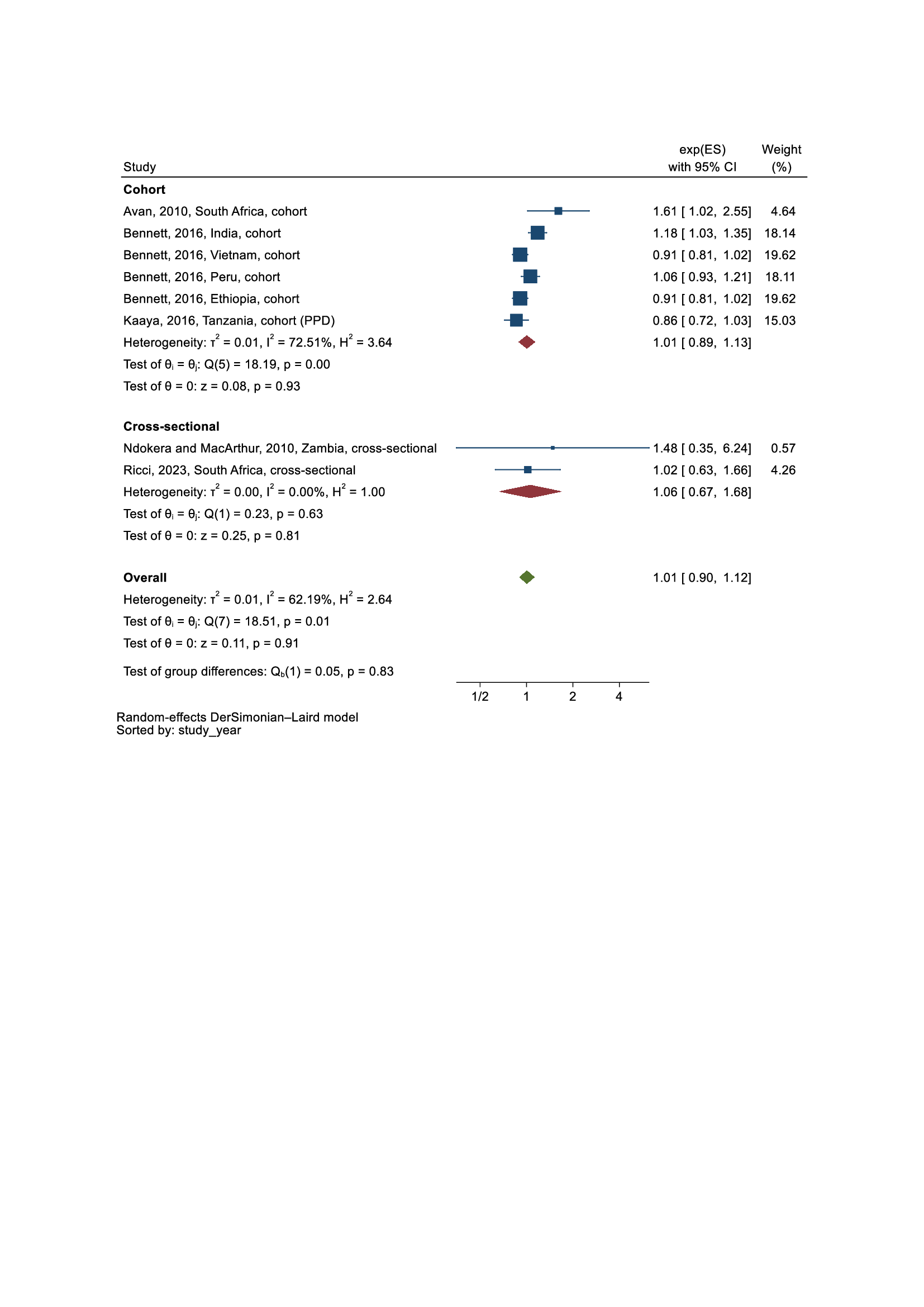

Supplement: S3 Fig — (TIF) [file pgph.0003586.s009.tif]

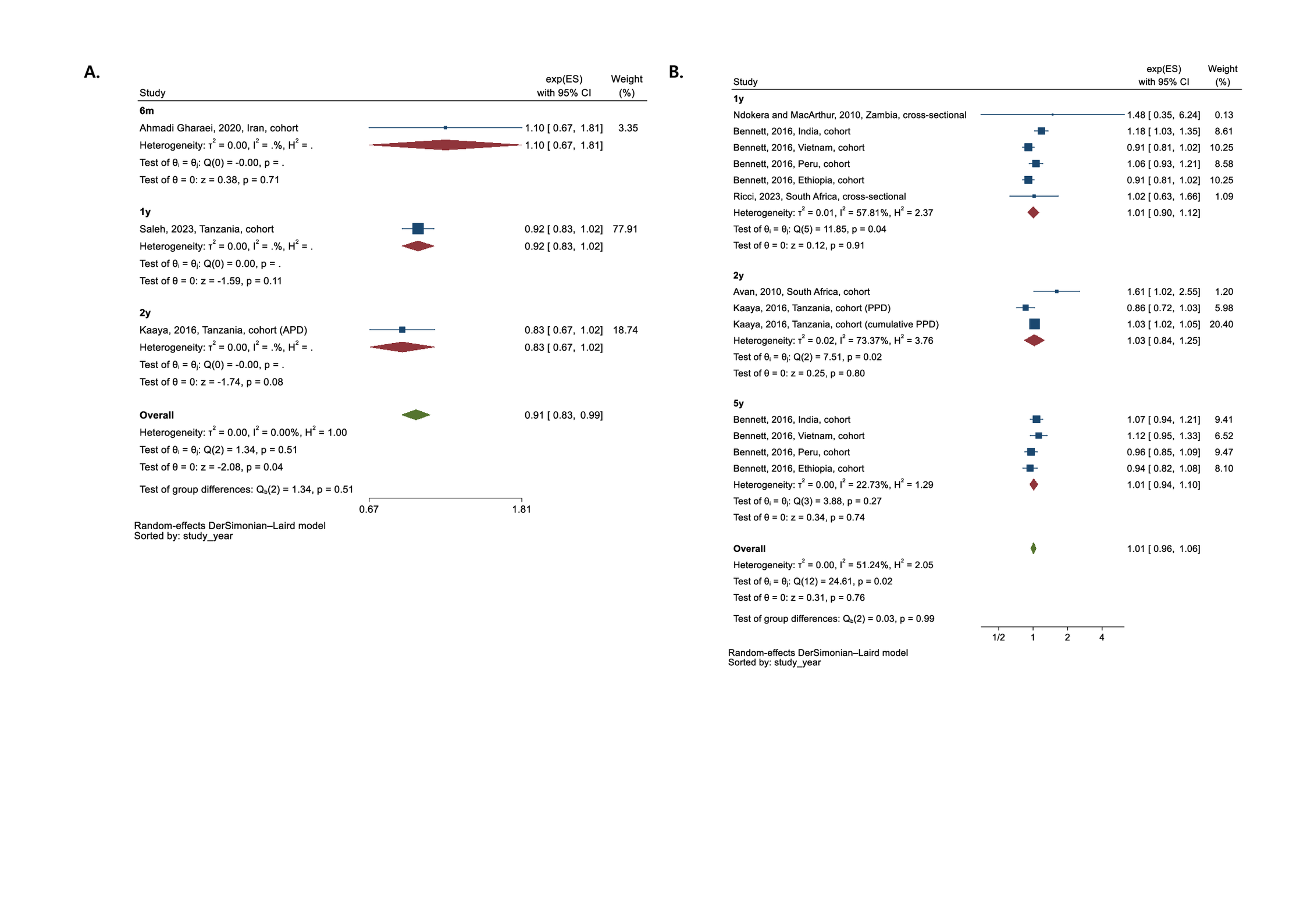

Supplement: S4 Fig — A. The risk ratio between antepartum depression and stunting timepoint of outcome measurement. B. The risk ratio between postpartum depression and stunting according to the timepoint of outcome measurement. (TIF) [file pgph.0003586.s010.tif]

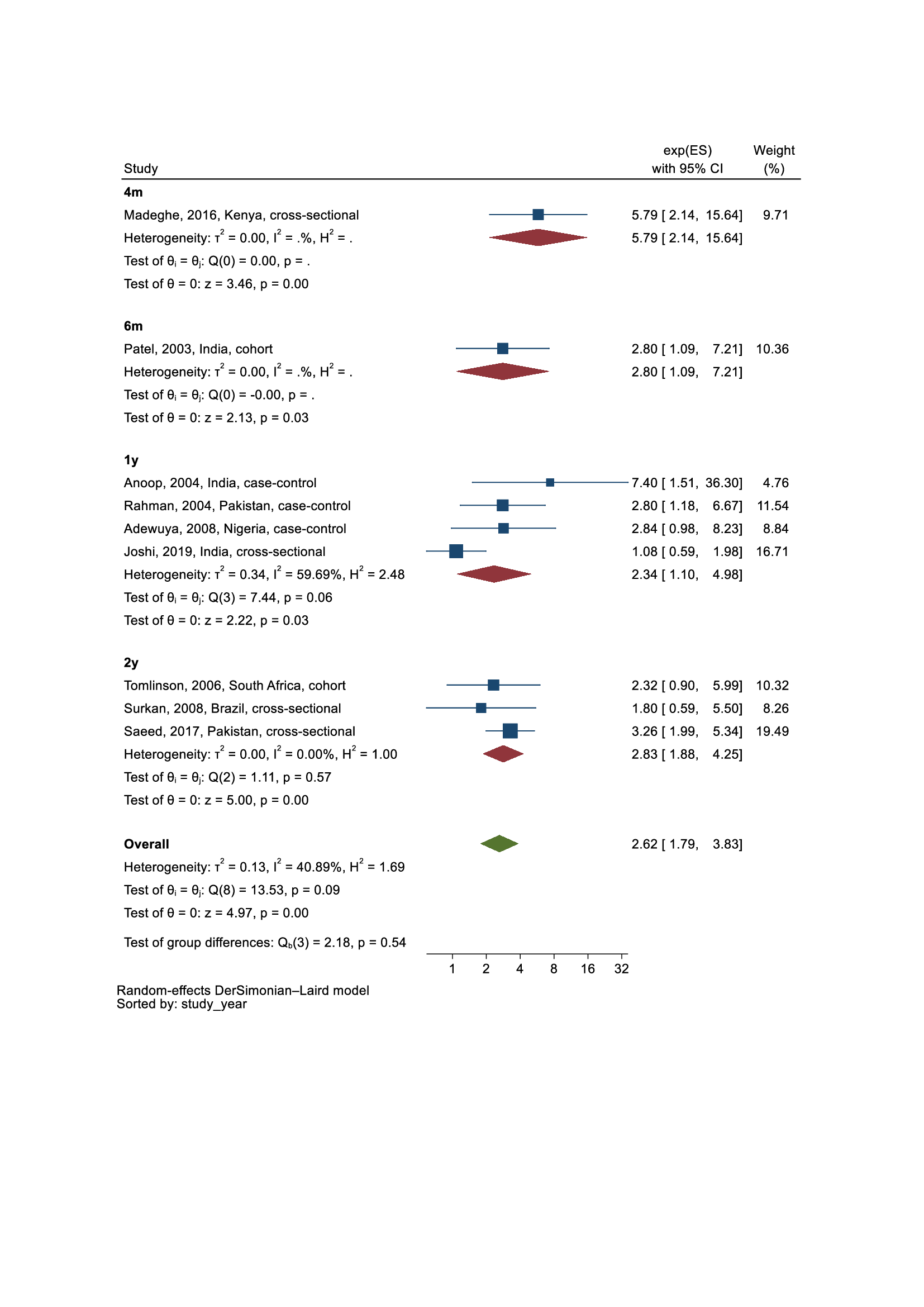

Supplement: S5 Fig — (TIF) [file pgph.0003586.s011.tif]

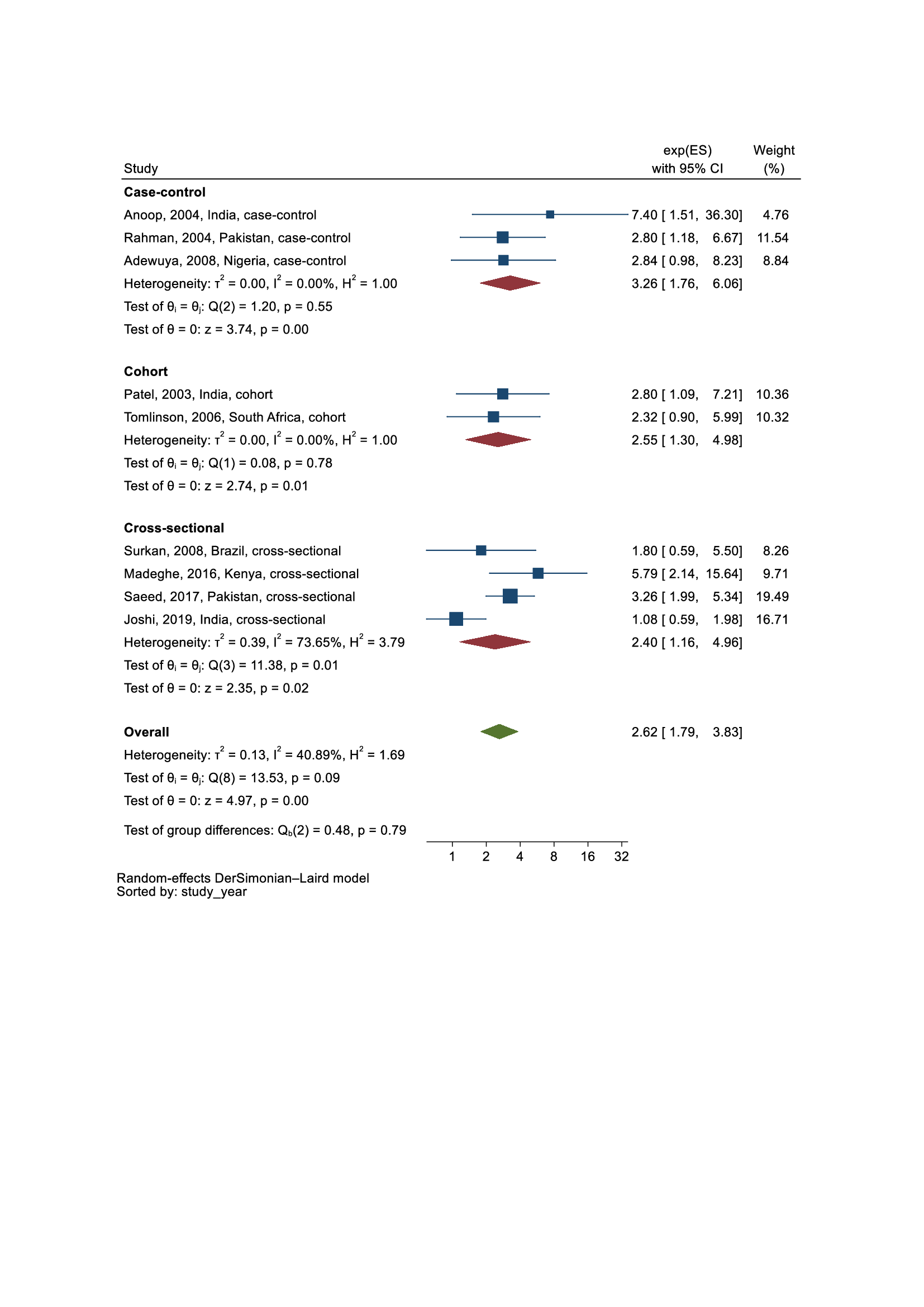

Supplement: S6 Fig — (TIF) [file pgph.0003586.s012.tif]

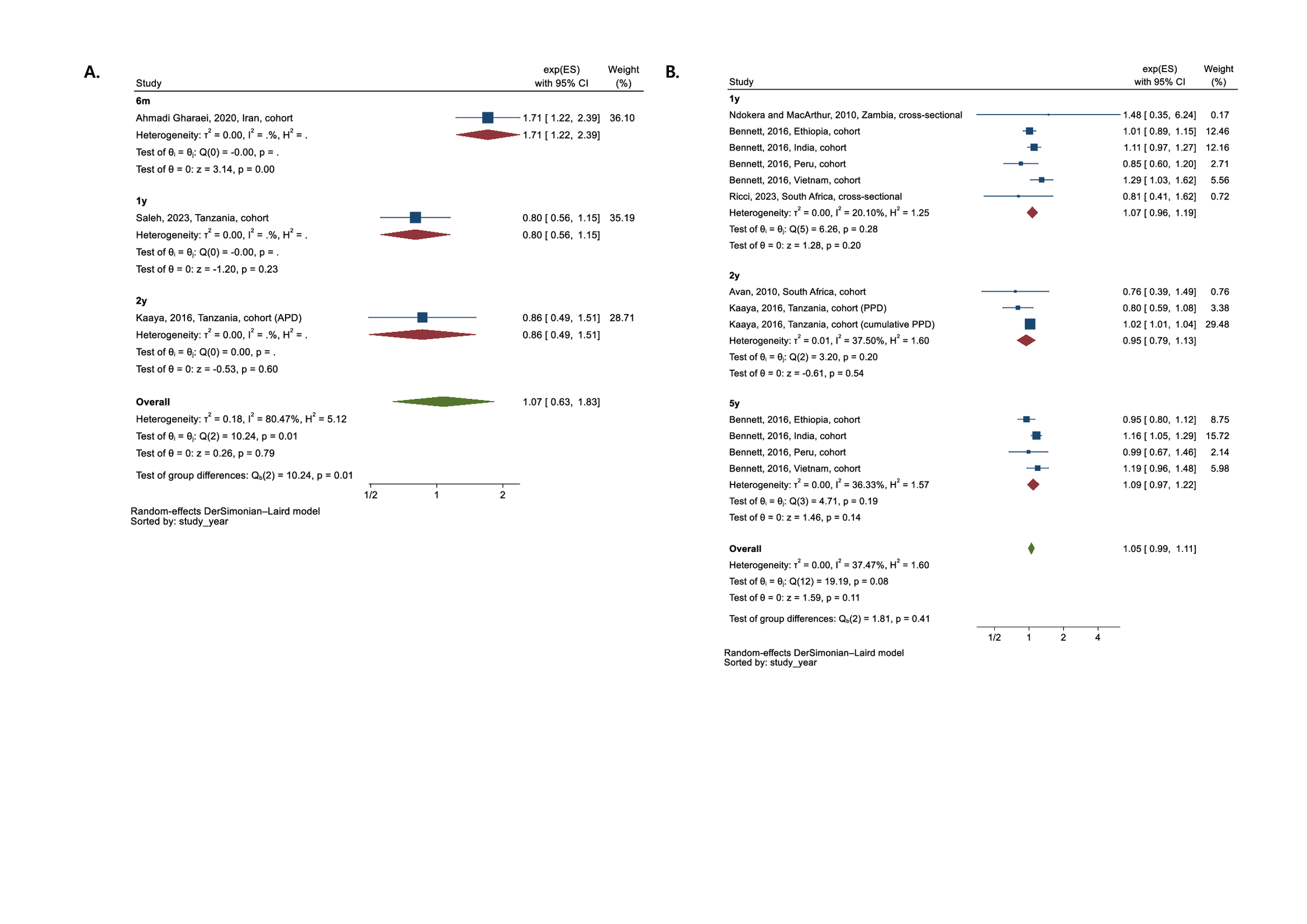

Supplement: S7 Fig — A. The risk ratio between antepartum depression and underweight according to outcome timepoint. B. The risk ratio between postpartum depression and underweight according to outcome timepoint. (TIF) [file pgph.0003586.s013.tif]

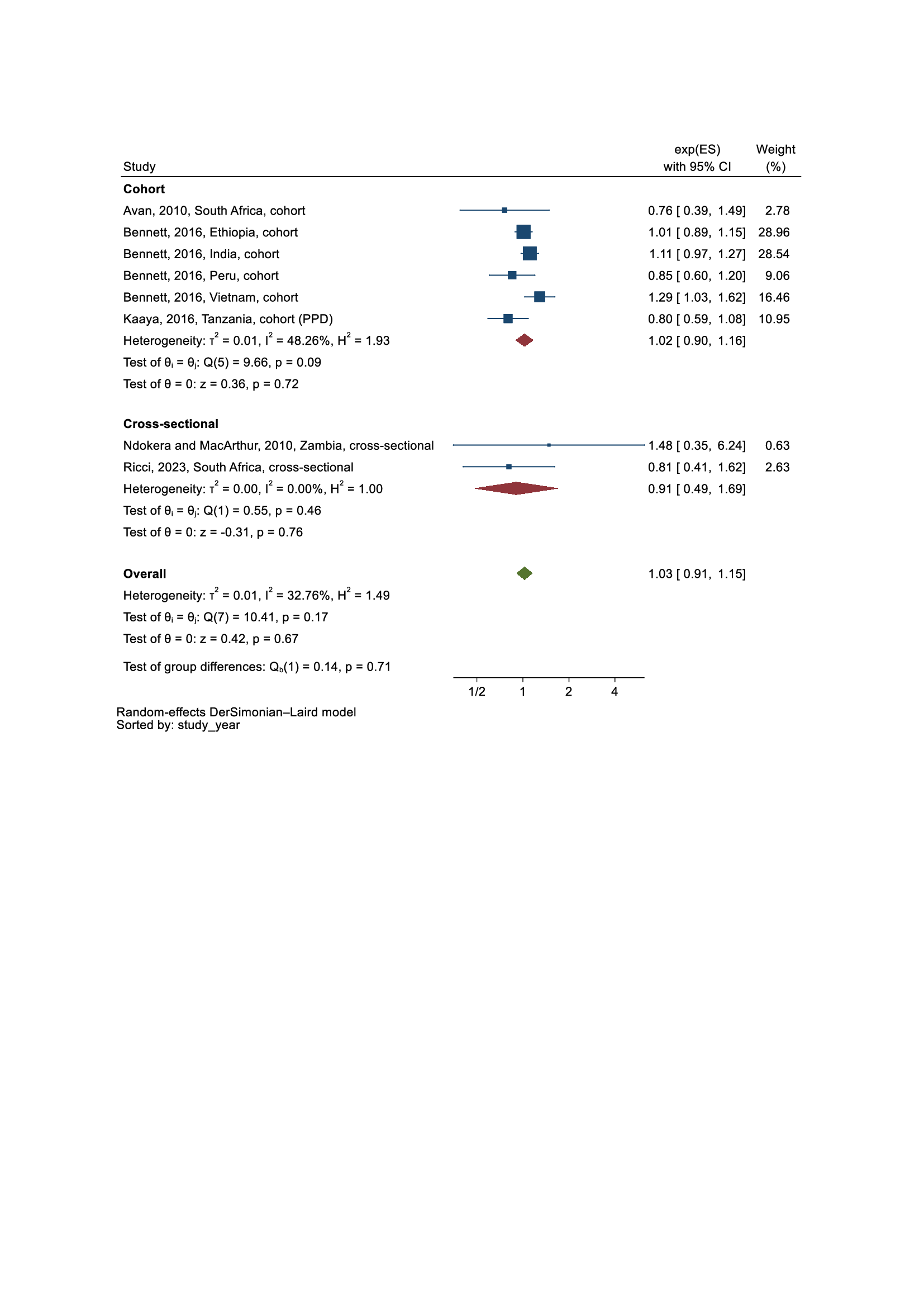

Supplement: S8 Fig — (TIF) [file pgph.0003586.s014.tif]

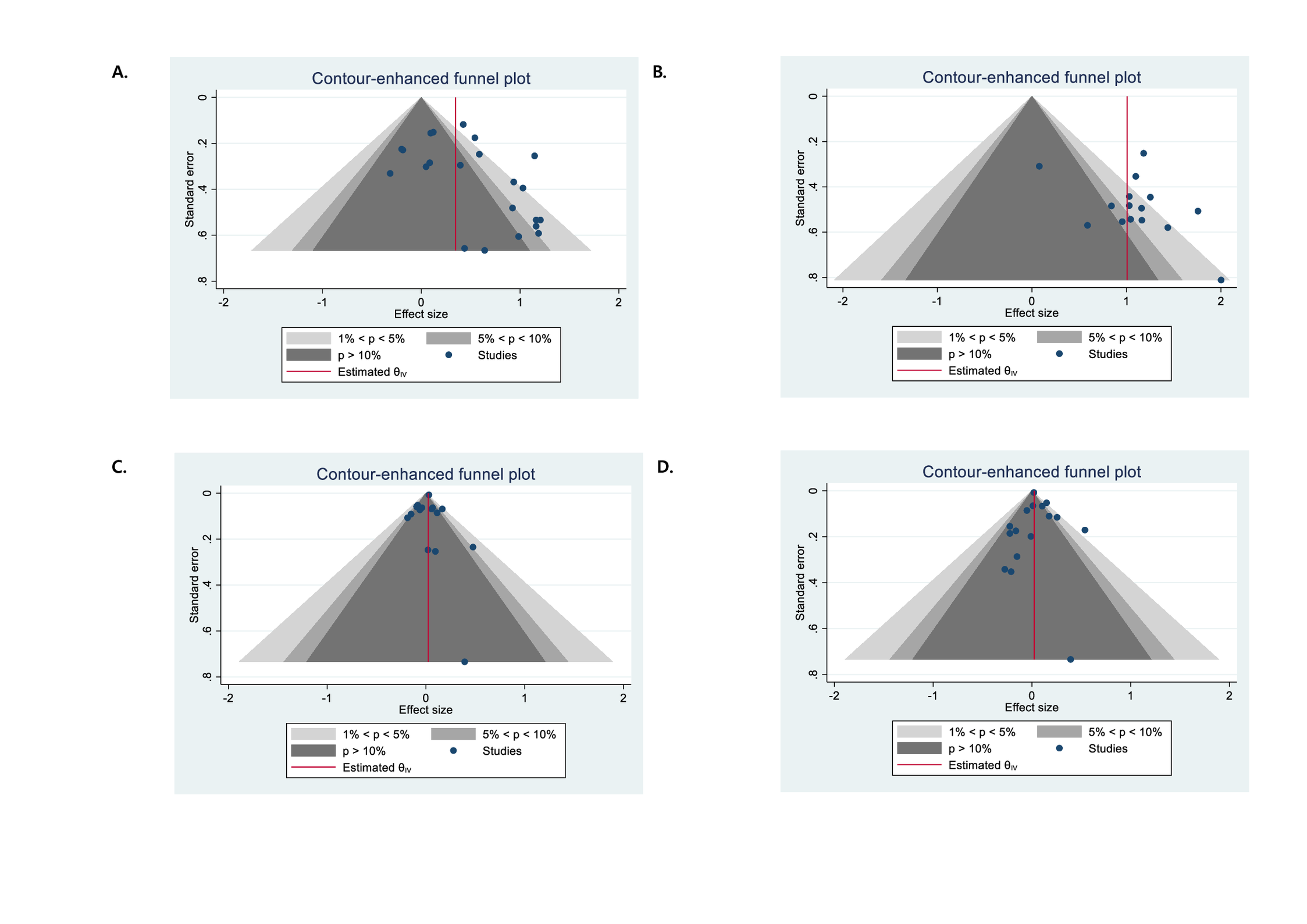

Supplement: S9 Fig — A. A funnel plot of studies reporting odds ratios between perinatal depression and stunting. B. A funnel plot of studies reporting odds ratios between perinatal depression and underweight. C. A funnel plot of studies reporting risk ratios between perinatal depression and stunting. D. A funnel plot of studies reporting risk ratios between perinatal depression and underweight. (TIF) [file pgph.0003586.s015.tif]

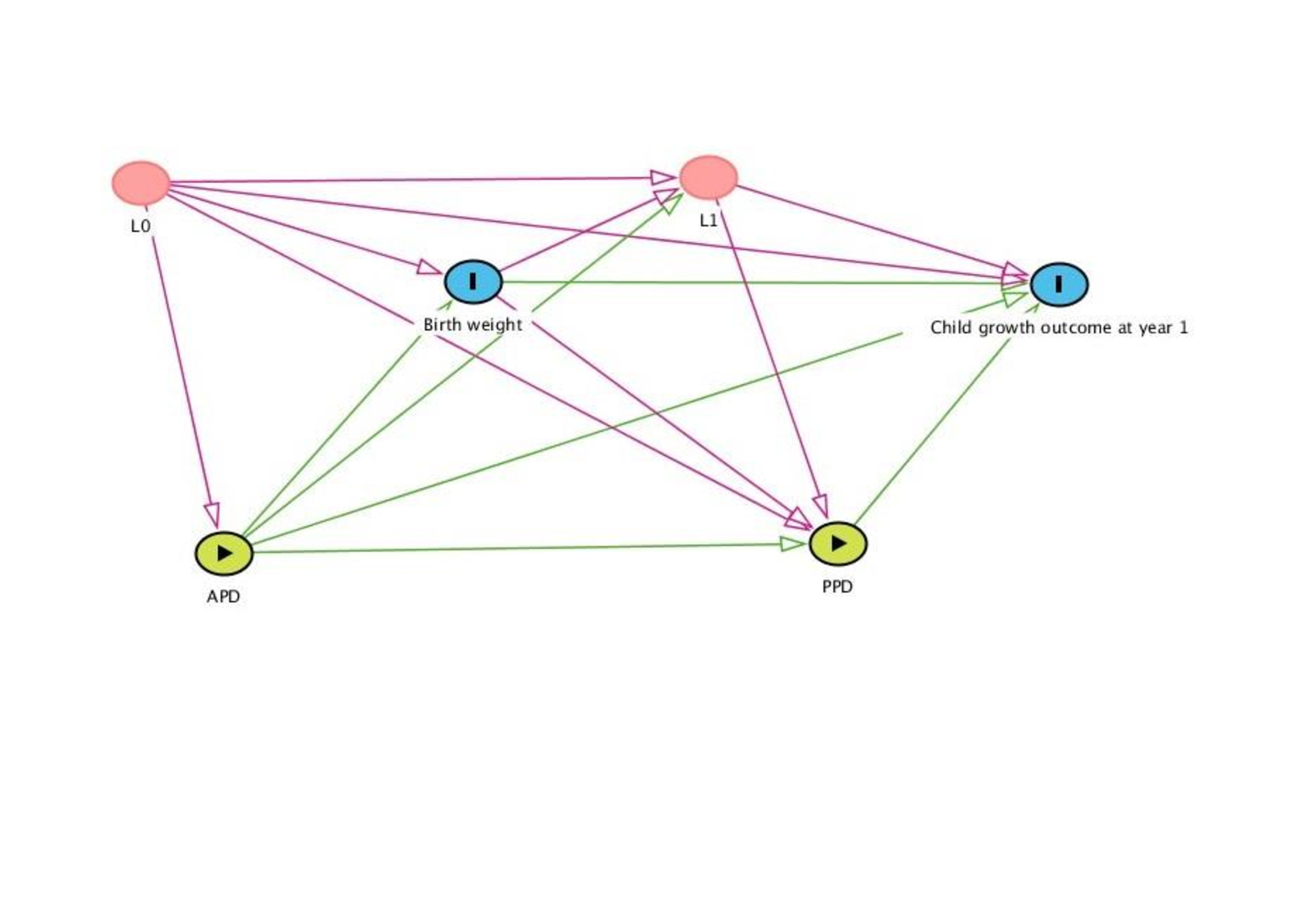

Supplement: S10 Fig — Note: APD = antepartum depression, PPD = postpartum depression, L0 = confounder before pregnancy, L1 = time-varying mediator-confounder after delivery (ie, family support). Time-varying exposure: APD and PPD. Time-varying outcome: birth weight and child growth outcome at year 1. Green arrows indicate the causal pathways we want to measure. Red arrows indicate the backdoor path that induces bias when we measure the association between exposure and outcome. (TIF) [file pgph.0003586.s016.tif]
