# Supplementary material for: Perinatal depression and adverse child growth outcomes in low-income and middle-income countries (LMICs): A systematic review and meta-analysis
Source: PLOS Glob Public Health. 2024 Oct 28;4(10):e0003586. doi: 10.1371/journal.pgph.0003586 (PMC11516009; doi:10.1371/journal.pgph.0003586)
Supplement: Attachment — Submitted filename: PGPH-D-23-02443_reviewer_Adumbire.pdf [file pgph.0003586.s018.pdf]

PLOS Global Public Health

Perinatal depression and adverse child growth outcomes in low- and middle-income countries (LMICs): a systematic review and meta-analysis

--Manuscript Draft--

**Manuscript Number:**	PGPH-D-23-02443
**Article Type:**	Research Article
**Full Title:**	Perinatal depression and adverse child growth outcomes in low- and middle-income countries (LMICs): a systematic review and meta-analysis
**Short Title:**	Perinatal depression and adverse child growth outcomes in low- and middle-income countries
**Corresponding Author:**	Hyelee Kim, MD, MAS, MS<br>University of California San Francisco<br>UNITED STATES
**Order of Authors:**	Elizabeth Carosella, MPH
	Shradha Chhabria, MD, MPH
	Hyelee Kim, MD, MAS, MS
	Aliya Moreira, MD, MPH
	Dana Naamani, MD, MPH
	Brennan Ninesling, MD, MPH
	Aimee Lansdale, MS
	Lakshmi Gopalakrishnan, PhD
	Bizu Gelaye, PhD
	Aisha Yousafzai, PhD
	Stefania Papatheodorou, MD, PhD
**Keywords:**	Perinatal depression; maternal depression; antepartum depression; postpartum depression; child growth outcome; child malnutrition; Stunting; underweight; overweight; obesity; wasting; low and middle income countries; LMICs
**Abstract:**	Perinatal depression (PND), which encompasses the antepartum and postpartum depression (APD and PPD) of mothers, is a neglected crisis in low- and middle-income countries (LMICs). We aimed to systematically search for and meta-analyze existing evidence to determine whether a mother’s PND affects adverse growth outcomes in children in LMICs (PROSPERO protocol: CRD42021246803).<br>We conducted searches across nine databases (PubMed, EMBASE, Web of Science, CINAHL Plus, Global Health Database, Google Scholar, WHO Regional Databases, PsycINFO, and LILACS) from January 2000 to September 2023. We restricted studies that assessed PND using validated screening tools or clinical interviews during pregnancy or within 12 months postpartum. We included studies that reported four types of adverse child growth outcomes, stunting, wasting, underweight, and overweight/obesity, in children younger than 5 years. We pooled risk ratios (RRs) and odds ratios (ORs) between PND and each adverse growth outcome using random-effects models.<br>In total, 27 studies met the inclusion criteria for systematic review, with 24 eligible for meta-analysis, spanning data from 15 countries and 26,261 mother-baby pairs. Based on the studies that reported ORs, children below the age of 3 years with mothers experiencing PND had higher odds for stunting (OR 1.63, 95% CI 1.32, 2.02, I2 = 56.0%) and underweight (OR 2.65, 95% CI 1.90, 3.68, I2 = 34.5%) compared to children of mothers without PND. The pooled RRs for stunting, wasting, and underweight did not show significant differences between mothers with and without PND, though the effects varied by region, type of PND, timepoint of the outcome, and study designs.<br>While we found heterogeneous effects when using different measures of association,

Powered by Editorial Manager® and ProduXion Manager® from Aries Systems Corporation

	the number of studies, especially those addressing overweight/obesity as an outcome and those conducted in diverse LMICs, was limited, calling the need to allocate resources to better understand this link in LMICs.
Additional Information:	
Question	Response
**Financial Disclosure**<br><br>Enter a financial disclosure statement that describes the sources of funding for the work included in this submission and the role the funder(s) played. This includes grants and any commercial funding of the work or authors.<br><br>This statement will be typeset if the manuscript is accepted for publication.<br><br>*Please review the submission guidelines and the instructions link below for detailed requirements and guidance.*	The authors received no specific funding for this work.
**Competing Interests**<br><br>On behalf of all authors, disclose any competing interests that could be perceived to bias this work.<br><br>This statement will be typeset if the manuscript is accepted for publication.<br><br>*Please review the instructions link below and PLOS Global Public Health's competing interests policy to determine what information must be disclosed at submission.*	The authors reported that they are any financial, personal, or professional interests that could be construed to have influenced the work.
**Data Availability**<br><br>Before publication, Authors are required to make fully available and without restriction all data underlying their findings. Please see our PLOS Data Policy page for detailed information on this policy.	The systematic review and meta-analysis were conducted based on publicly available data from multiple electronic databases. The search strategies, inclusion and exclusion criteria, and data extraction forms used in this study are provided in the main manuscript and supplements. Please contact the corresponding author if further information is needed.

A **Data Availability Statement**, detailing where the data can be accessed, is required at first submission. Insert your Data Availability Statement in the box below.<br><br>Please see the data reporting section of our submission guidelines for instructions on what you need to include in your Data Availability Statement.<br><br>This statement will be typeset if the manuscript is accepted for publication.<br><br>*PLOS allows rare exemptions to address legal and ethical concerns. If you have legal or ethical restrictions, please use the box below to detail these in full sentences for the Journal team to consider.*	

Powered by Editorial Manager® and ProduXion Manager® from Aries Systems Corporation

Manuscript

Click here to access/download;Manuscript;PLOS_meta-analysis_revision_final.docx

Perinatal depression and adverse child growth outcomes in low- and middle-income countries (LMICs): a systematic review and meta-analysis

Elizabeth Carosella[1¶], Shradha Chhabria[1#a¶], Hyelee Kim[1#b¶*], Aliya Moreira[1#c¶], Dana Naamani[1#d¶], Brennan Ninesling[1#e¶], Aimee Lansdale[2†], Lakshmi Gopalakrishnan[2†], Bizu Gelaye[1], Aisha Yousafzai[1], Stefania Papatheodorou[1]

[1] Harvard T.H. Chan School of Public Health, Boston, Massachusetts, United States of America

[2] University of California San Francisco, California, United States of America

[#a] Current address: University Hospitals Cleveland Medical Center and Rainbow Babies and Children's Hospital, Cleveland, Ohio, United States of America

[#b] Current address: University of California San Francisco, California, United States of America

[#c] Current address: Beth Israel Deaconess Medical Center, Boston, Massachusetts, United States of America

[#d] Current address: Duke University Medical Center, Durham, North Carolina, United States of America

[#e] Current address: University of California San Diego, California, United States of America

¶ These authors equally contributed to this work.

† These authors equally contributed to this work.

*** Corresponding author**

Hyelee.Kim@ucsf.edu (HK)

Abstract (~300 words)

Perinatal depression (PND), which encompasses the antepartum and postpartum depression (APD and PPD) of mothers, is a neglected crisis in low- and middle-income countries (LMICs). We aimed to systematically search for and meta-analyze existing evidence to determine whether a mother's PND affects adverse growth outcomes in children in LMICs (PROSPERO protocol: CRD42021246803).

We conducted searches across nine databases (PubMed, EMBASE, Web of Science, CINAHL Plus, Global Health Database, Google Scholar, WHO Regional Databases, PsycINFO, and LILACS) from January 2000 to September 2023. We restricted studies that assessed PND using validated screening tools or clinical interviews during pregnancy or within 12 months postpartum. We included studies that reported four types of adverse child growth outcomes, stunting, wasting, underweight, and overweight/obesity, in children younger than 5 years. We pooled risk ratios (RRs) and odds ratios (ORs) between PND and each adverse growth outcome using random-effects models.

In total, 27 studies met the inclusion criteria for systematic review, with 24 eligible for meta-analysis, spanning data from 15 countries and 26,261 mother-baby pairs. Based on the studies that reported ORs, children below the age of 3 years with mothers experiencing PND had higher odds for stunting (OR 1.63, 95% CI 1.32, 2.02, I^2 = 56.0%) and underweight (OR 2.65, 95% CI 1.90, 3.68, I^2 = 34.5%) compared to children of mothers without PND. The pooled RRs for stunting, wasting, and underweight did not show significant differences between mothers with and without PND, though the effects varied by region, type of PND, timepoint of the outcome, and study designs.

While we found heterogeneous effects when using different measures of association, the number of studies, especially those addressing overweight/obesity as an outcome and those conducted in diverse LMICs, was limited, calling the need to allocate resources to better understand this link in LMICs.

INTRODUCTION

Perinatal depression (PND), a nonpsychotic depressive episode of mild to major severity, affects 11.9% of mothers during pregnancy or after delivery worldwide,(1) with significant reductions in quality of life and day-to-day functioning, with potentially far-reaching consequences for range of adverse child health outcomes.(2,3) PND encompasses depression among mothers during pregnancy, antepartum depression (APD), and depression after delivery, postpartum depression (PPD), depending on the timing of symptom onset.(4,5) Research has suggested a higher prevalence of PND in low- and middle-income countries (LMICs) compared to high-income countries where epidemiologic estimates are well studied (1,6) and in LMICs, over 25% and 19% of women suffer from PND and PPD, respectively.(7)

Maternal health during and after pregnancy plays a critical role in children's health. The first 1,000 days from conception to two years of age is a particularly sensitive period for children's development and sets the stage for their future growth trajectories. Moreover, eating behaviors and food preferences, influenced by parental care practices, become established during the first five years.(8,9) A systematic review and meta-analysis in Africa highlighted a strong association between APD and adverse birth outcomes, such as preterm birth and low birth weight, while PPD was also highly associated with infant growth and health outcomes.(10) Further, studies have shown that PPD in the LMICs could contribute to poor infant attachment and interactions with caregivers, potentially contributing to developmental and emotional challenges in children's future.(6) Inadequate nutrition during this time may lead to negative health outcomes for children, including cognitive impairment, compromised immune systems, and an increased risk of cardiometabolic disease.(11)

Globally, nearly half of child deaths are associated with malnutrition(12). According to the UNICEF/WHO/World Bank Group Joint Child Malnutrition Estimates, 149.2 million children

below five years were affected by stunting, 45.4 million by wasting, and 28.9 million by overweight.(13) Despite progress in recent decades, stunting and wasting rates remain above-established targets, while the prevalence of overweight is on the rise.(14) Neglecting to address PND may be contributing to these trends, with severe consequences for children's health, development, and future economic participation.(15) Moreover, the COVID-19 pandemic is expected to heighten the risk of all forms of child malnutrition and poor maternal mental health.(16,17) As part of efforts to achieve Sustainable Development Goal 2 and end all forms of malnutrition by 2030,(18) maternal mental health merits higher priority on the global public health agenda.

While a growing body of research suggests that PND is a risk factor for various suboptimal child cognitive, behavioral, and developmental outcomes,(19,20) there is relatively less evidence exists for the relationship between perinatal, maternal mental health and child growth outcomes in LMICs.(21,22) A synthesis of the available evidence for this relationship is useful to understand these relationships and for highlighting gaps in the existing literature.

The objective of this systematic review and meta-analysis was to assess whether PND has an impact on four types of adverse growth outcomes (stunting, wasting, underweight, overweight/obesity) among children under five years in LMICs using existing studies. While we considered the associations reported in the previous studies, our primary interest lay understanding the temporal relationship between PND and adverse child growth outcomes. Thus, we hypothesized that the PND occurring before would exhibit a statistically significant association with adverse child growth outcome in the pooled analyses. In contrast to previous meta-analyses on this topic, we intended to estimate the effect of exposures to APD and PPD separately, or as a composite PND on a broader range of adverse growth outcomes, including wasting, and overweight/obesity. This meta-analysis also included studies spanning a longer

time interval and a more diverse set of LMICs, including from Latin America, which have been underrepresented in previous meta-analyses.(23–26)

Methods

We defined PND as a diagnosis made during pregnancy or within 12 months postpartum. Our primary exposure of interest was PND, rather than maternal depression. Therefore, in cases where the authors reported maternal depression instead of PND, we limited the measurement time point for PPD to a period up to 12 months after delivery to prevent reverse causation between the exposure, PPD, and adverse growth outcomes. We reported the pooled odds ratios (ORs) and the pooled risk ratios (RRs) for child growth outcomes for both disaggregated exposures (APD and PPD) and the composite exposure (PND).(4) Given that each growth outcome has unique environmental and biological underpinnings, our aim was to report multiple disaggregated adverse growth outcomes, specifically, stunting, underweight, wasting, and overweight/obesity. This study adhered to the pre-registered protocol on PROSPERO International prospective register of systematic reviews (registration number: CRD42021246803, assessment url: https://www.crd.york.ac.uk/prospero/display_record.php?RecordID=246803).(27) We have presented the PRISMA 2020 Checklist in S1 Table.

Stunting was defined as height or length-for-age z-scores (HAZ or LAZ) of two standard deviations (SD) or more below the WHO Child Growth Standards median by sex.(28) Underweight was defined as weight-for-age z-scores (WAZ) of two SDs or more below the WHO Child Growth Standards median by sex.(28) Wasting was defined as weight-for-length or height-for-length z-scores (WLZ or HLZ) of two SDs or more below the WHO Child Growth Standards median by sex.(28) Overweight was defined as WLZ or HLZ of two SDs or more from the WHO Child Growth Standards median by sex, and obesity was defined as WLZ or

HLZ of three SDs or more from the WHO Child Growth Standards median by sex.(28) Given the nature of the systematic review, institutional review board approval was not required.

Data Source

We conducted searches in the following databases: PubMed, EMBASE, Web of Science, CINAHL Plus, Global Health Database (EBSCO), Google Scholar, WHO Regional Databases (Global Index Medicus), PsycINFO, and Latin American and Caribbean Health Sciences Literature (LILACS). Our initial search extended up to March 2021 and during the revision process, we updated the search until September 2023. The exact search terms used for each of the databases are provided in the *Supplement*. Additionally, we supplemented our search with hand search of the Women's Mental Health Journal, Conference abstracts of the International Marcé Society for Perinatal Mental Health, the WHO working group on PND, and related information and studies from websites of the Center for Global Mental Health and ReliefWeb.

Study Selection and Data Extraction

Identified references were uploaded to Covidence.org for the review process.(29) Eight reviewers completed title and abstract screening, full-text review, and data extraction (E.C., H.K., B.N., S.C., D.N., A.M., A.L., and L.G.). Each title and abstract, and subsequently each full-text study, underwent reviewed by two authors chosen from the group (E.C., H.K., B.N., S.C., D.N., A.M., A.L., and L.G.). Articles selected by both authors were included in the study. For articles selected by only one author, the team discussed its suitability for inclusion, and a consensus was reached.

Studies were considered eligible if they met the following criteria: 1) was an original research article published in a peer-reviewed journal; 2) contained demographic and clinical

information on mother-baby pairs; 3) the exposure was either APD or PPD, diagnosed during pregnancy or up to 12 months after delivery; 4) depression diagnosis was based on a validated patient questionnaire or clinical interview; 5) the outcome was classified as stunting, wasting, underweight, overweight, or obesity among children under the age five; 6) conducted in an LMIC as defined by the World Bank;(30) 7) reported relevant quantitative measures, such as ORs, RRs, or other metrics suitable for meta-analysis (e.g., % proportion or beta-coefficients); and 8) the publication was available in English, Spanish, Portuguese, or French.

We excluded studies that were literature reviews, systematic reviews and/or meta-analyses, as well as clinical trials. When a study conducted secondary data analysis on data from a randomized controlled trial (RCT) as an observational study, we assumed that the path from randomization to PND did not exist due to the inclusion of randomization. Consequently, if there was no backdoor path between PND and adverse growth outcomes, observational studies conducted within RCTs were included. If the studies used PND measurement addressing heterogeneous symptoms of depression, anxiety, and psychological distress, we included them if the association for depression was reported, or if the authors demonstrated that the measure was validated for depression in the study population. In case where adverse growth outcomes were defined by criteria outside the inclusion criteria, we included the studies based on consensus. Studies were excluded if they solely assessed adverse birth outcomes (e.g. low birth weight) or if the definition of adverse growth outcomes were omitted in the paper.

Since our research question focuses on adverse growth outcomes, we excluded studies that analyzed the growth outcome indicators, such as height-/length-for-age Z score (HAZ/LAZ), weight-for-age Z score (WAZ), or height-/length-for-weight Z score (HWZ/LWZ), as opposed to adverse growth outcomes. However, during the full-text review, it became apparent that many studies examined the association between maternal depression outside

the PND timeframe (e.g. maternal depression of 4-year-old child) and child growth outcomes that were not classified as adverse. While these findings were not the primary focus of our research, we have included a summary in S2 Table due to their relevance to our main research question. This table includes 30 studies, which including additional findings from four studies within the final selection for systematic review and meta-analysis.(31–60)

Standardized data collection forms were developed by two authors (B.N. and E.C.), and data extraction was performed by a team of two authors (D.N., H.K., S.C., A.M., B.N., E.C., A.L., and L.G). The data extracted included the following variables: authors, publication year, country, study design, number of mother-child pairs, the tool and cut-point used for PND measurement, timing of PND measurement, the type and definition of adverse growth outcomes, including the timing of adverse growth outcome measurement, adjusted covariates, measures of association (types and values), and their corresponding 95% confidence intervals (CIs).

Quality Assessment

The quality of the included studies was assessed by five authors (B.N., D.N., H.K., A.L., and L.G.), who evaluated studies using the Newcastle Ottawa Scale (NOS) (S3 Table).(61) Each study underwent assessment by two reviewers (D.N. and H.K.), and a third reviewer (B.N., A.L., or L.G.) provided the final approval and acted as a tiebreaker in the case of any discordance. One component of quality assessment for cohort studies examined whether the outcome of interest was absent at the baseline. However, given our research focus on the path from APD through adverse birth outcomes, we decided that this requirement was irrelevant to our research question. Consequently, we did not deduct any point for this component, regardless of whether the outcome was reported at baseline.

Studies with a score of 7 or higher out of a total score of 9 were categorized as "good quality." Nearly all studies, with the exception of one, got scores of seven or above on the NOS scale, demonstrating sufficient quality for inclusion in the study. It is worth noting that while these studies were deemed to have a good quality, each study may not have been entirely free from bias due to various factors, such as missing observations, residual and unmeasured confounding, measurement bias. Joshi and Raut (2019) received a score of 5 due to the absence of attrition rate reporting and the use of unadjusted reported measures of association.

Synthesis

While our initial aim was to perform meta-analyses encompassing children up to 5 years, it was observed that nearly all the included studies assessed growth outcomes in children aged less than 32 months. The exception was a single study, Bennett et al. (2016), which included four sub-studies and reported the outcomes at the age of five years.(62) In cases where outcomes were repeatedly measured in a longitudinal cohort study, the associations measured at different time points were inherently correlated with. Consequently, including all of these estimates in the meta-analysis would have artificially reduced the CIs.(63) To address this, we selected one of the estimates in such cases.(63) To ensure homogeneity in the timepoints of outcomes, the primary meta-analysis focused on estimates reported closest to the one-year mark. For example, in Patel et al. (2003), we selected the adjusted OR (aOR) among children aged six months rather than 6 weeks. For estimates that were repeated at various time points of outcome, we conducted the subgroup analyses to understand the trend of the association based on the time points of outcome.

Due to the limited number of studies using a consistent measure of association for overweight/obesity, the primary meta-analysis focused only on stunting, underweight, and wasting. Additionally, recognizing the heterogeneity in RR and OR, we stratified the analysis

based on the type of measure of association (RR and OR). Meta-analyses were conducted using both the DerSimonian and Laird methods (DL) under a random-effects model and the Knapp–Hartung (KH) standard-error adjustment estimates (also known as Sidik–Jonkman adjustment), given the relatively small number of studies for each outcome.(64,65) We reported the overall effect of PND on each adverse growth outcome performed subgroup analyses based on the timing of depression diagnosis (APD vs. PPD), time point of measurement of adverse growth outcome (4 months, 6 months, 1 year, 2 years, and 5 years), region (Africa, America, Asia, and Middle-East), and study design (case-control, cohort, cross-sectional, and secondary analysis in RCT).

Sensitivity analyses were conducted to assess the influence of 1) unadjusted estimates on the pooled estimate, 2) inclusion of maternal depression measured up to 24 months, and 3) studies published more than 10 years ago. Additionally, we performed meta-regressions, considering country income category, region, and duration of follow-up. Funnel plots and Egger's test were conducted to examine potential publication bias.(66) To evaluate heterogeneity, we followed the guidelines recommended by the Cochrane Statistical Methods Group: I^2 30% to 60% moderate heterogeneity, 50% to 90% substantial heterogeneity, and 75% to 100% indicating considerable heterogeneity.(67,68) All analyses were performed using Stata Version 17.(69)

Results

Description of studies

During the initial database search in 2021, we identified 1,944 records and assessed titles and abstracts of 1,414 articles (Fig 1). From this set, we conducted the full text review of 129 articles, except for four studies that we could not locate (e.g., the conference abstract). In

cases where specific estimates required for our research question were not reported, particularly when maternal depression was measured up to 5 years after birth, we made efforts to contact the authors to obtain the relevant estimate (n = 24). Among these efforts, one group, Prado et al. (2019), provided the requested estimate.(70) Additionally, three studies, Surkan et al. (2008), Kaaya et al. (2016), and Wemakor (2018), that measured PPD beyond the upper limit of our inclusion criteria (up to 24 months) were included in the systematic review and meta-analysis, and we excluded them in the sensitivity analysis.

Fig 1. PRISMA flowchart.

From: Page MJ, McKenzie JE, Bossuyt PM, Boutron I, Hoffmann TC, Mulrow CD, et al. The PRISMA 2020 statement: an updated guideline for reporting systematic reviews. BMJ 2021;372:n71. doi: 10.1136/bmj.n71

Note: LMICs = low- and middle-income countries

Three studies, namely Nasreen et al. (2013), Brentani and Fink. (2016), and Christodoulou (2019), met the other inclusion criteria but did not report the measure of association suitable for pooling in the meta-analysis.(38,60,71) Consequently, these studies were included in the systematic review but excluded in the meta-analysis. One study by Bennett et al. (2016), combined sub-studies conducted in India, Ethiopia, Peru, and Vietnam, and each sub-study was analyzed as a separate study.(62) As a result, the systematic review encompassed 24 studies (including 27 sub-studies), while the meta-analysis included 21 studies (comprising 24 sub-studies) during the initial search.(38,39,60,62,70–89)

During the search update in September 2023, we identified 755 studies and screened 573 studies based on their titles and abstracts (Fig 1). Among these, we conducted a full text review of 34 studies, and ultimately including 3 studies in the systematic review and meta-

analysis.(43,90,91) As a result, across the two database search processes, a total of 27 studies (30 sub-studies) were included in the final systematic review (38,39,43,60,62,70–91) (Table 1). These 27 studies covered 24 studies on stunting, 19 studies on underweight, 2 studies on overweight/obestiy, and 5 studies on wasting. In the final meta-analysis, we pooled estimates from a total of 27 sub-studies (n of studies = 24), (39,62,70,72–89): 21 studies (24 sub-studies) were on stunting, 17 studies (20 sub-studies) on underweight, and 3 studies on wasting.

Among the total studies included in the systematic review (n = 27), fourteen (52%) were cohort studies, nine (33%) were cross-sectional, three (11%) were case-control studies, and 1 (4%) was a secondary data analysis (cohort study) within an RCT. Only 7 studies (26%) utilized APD, including those that measured depression at birth or within 2 days after birth. All studies that reported the risk ratio between APD and stunting or underweight were cohort studies. Eighteen studies (67%) measured depression as PPD, and 2 studies (7%) assessed both APD and PPD.

Among the studies included in the meta-analysis for the association between PND and stunting (n = 21), 14 (67%) reported ORs and 7 (33%) studies reported RRs; seven APD studies (33%), 12 PPD studies (57%), one study (5%) that examined APD and PPD separately, and one study (5%) that used PND as a combined exposure. In the case of the meta-analysis for the association between PND and underweight, which included 17 studies, 10 (59%) reported ORs, and 7 (41%) reported RRs. Among these, 3 studies focused on APD (18%), 13 on PPD (76%) studies, and one study (6%) examined APD and PPD separately. For the meta-analysis of the association between PND and wasting, which encompassed 3 studies, all reported RRs. This set included 1 APD study (33%) and 2 PPD (67%) studies.

Various screening tools and their associated cutoff scores, as well as clinical diagnostic measures were employed to identify mothers with PND. Among the utilized questionnaires, the Edinburgh Postnatal Depression Scale (n = 11) was the most common measure of depression, followed by the Self-Reporting Questionnaire-20 (n = 4), Center for Epidemiological Studies Depression scale (n = 3), Hopkins Symptom Checklist (n = 2), Aga Khan University Anxiety and Depression Scale (n = 1), Depression Anxiety Stress Scales (n = 1), and Pitt Depression Inventory (n = 1) (Table 1). In most cases, the authors described in-detail the reasons for the selected questionnaire, such as reliability and validity, and for the selected cutoff score, the process of the local adaptation, or the use of an interpreter due to literacy issues. The semi-structured or structured clinical interview, like the Schedules for Clinical Assessment in Neuropsychiatry (n = 1), and Structured Clinical Interview for Diagnostic and Statistical Manual of Mental Disorders-III-R or -IV (n = 3) were also applied by the trained researchers or psychiatrists.

Among the total sub-studies included in the systematic review (30 sub-studies from 27 studies), six were conducted in India, four from South Africa, three each from Pakistan and Brazil, two from Ethiopia, Ghana, and Tanzania, and one each from Bangladesh, Vietnam, Iran, Kenya, Nigeria, Zambia, Zimbabwe, and Peru: Asia 11 studies, Middle East I study, Africa 14 studies, and America 4 studies. A descriptive summary of all studies included in the analysis and adjusted covariates in each study are shown in Tables 1 and 2, respectively.

Table 1. Summary of the study characteristics included in the systematic review and meta-analysis for the association between perinatal depression and child adverse growth outcomes (stunting, underweight, overweight/obesity) in the low- and middle-income countries the from 2003 to 2023 (N = 26,261).

First author (year)	Country	Study design	Sample size	Depression measure (cutoff)	Time of depression measure	Association measure	Time of outcome measure (after-birth)[a]	Stunting estimate (95% CI)	Underweight /overweight or obesity [b] estimate (95% CI)
Patel (2003)	India	Cohort	171	EPDS (≥11)	6 weeks postpartum	aOR	6 weeks	1.9 (0.5, 6.8)	3.2 (1.1, 9.4)
							6 months	3.2 (1.1, 8.9)	2.8 (1.1, 7.3)
Rahman (2004, 1)	Pakistan	Case-control	172	SRQ20 (≥ 10)	> 6 weeks postpartum	aOR	9 months		2.8 (1.2, 6.8)
Rahman (2004, 2)	Pakistan	Cohort	265	SCAN	3rd trimester	aOR	6 months	3.2 (1.1, 9.9)	3.5 (1.5, 8.6)
							12 months	2.8 (1.3, 6.1)	3.0 (1.5, 6)
Anoop (2004)	India	Case-control	144	SCID for DSM-III-R	< 6 weeks postpartum	aOR	6-12 months		7.4 (1.6, 38.5)
Tomlinson (2006)	South Africa	Cohort	147	SCID for DSM-IV	2 months postpartum	OR	18 months	2.52 (0.98, 6.47)	2.32 (0.90, 6.00)
Surkan (2008)	Brazil	Cross-sectional	595	CES-D (≥16)	6-24 months postpartum [c]	aOR	6-24 months	1.8 (1.1, 2.9)	1.8 (0.6, 5.6)
Adewuya (2008)	Nigeria	Case-control	242	SCID for DSM-III-R	< 6 weeks postpartum	OR	6 weeks	1.55 (0.43, 5.65)	2.60 (0.87, 7.62)
							3 months	3.28 (1.03, 10.47)	3.19 (1.21, 8.40)
							6 months	3.34 (1.18, 9.55)	4.21 (1.36, 13.20)
							9 months	2.68 (0.82, 8.80)	2.84 (0.98, 8.24)
Avan (2010)	South Africa	Cohort	892	PDI (≥19)	> 6 weeks postpartum	aRR	24 months	1.61 (1.02, 2.56)	0.76 (0.39, 1.49)
Ndokera (2010)	Zambia	Cross-sectional	278	SRQ20 (≥8)	> 6 weeks postpartum	RR	2-12 months	1.48 (0.35, 6.22)	1.48 (0.35, 6.22)
Nasreen (2013)[h]	Bangladesh	Cohort	652	EPDS	Third trimester	Adjusted beta-coefficient	6-8 months	-0.26[i] (-0.50, -0.01)	
					2-3 months postpartum			-0.26[i] (-0.54, 0.01)	-0.38[i] (-0.72, -0.05)
Brentani and Fink (2016)[h]	Brazil	Cross-sectional	798	EPDS (>13)	1 year	Adjusted beta-coefficient	1 year	0.02 (SE 0.03)	0.01 (SE 0.05)
Kaaya (2016)	Tanzania	Cohort	699	HSCL-25	During pregnancy	aRR	< 2 years	0.83 (0.67, 1.02)	0.86 (0.68, 1.09)
					<2 years postpartum[c]			0.86 (0.72, 1.03)	0.80 (0.54, 0.99)
					Cumulative postpartum			1.03 (1.01, 1.04)	1.02 (1.00, 1.04)
Madeghe (2016)	Kenya	Cross-sectional	200	EPDS (≥13)	6-16 weeks postpartum	aOR	6-16 weeks		5.79 (2.14, 15.62)
Upadhyay and	India	Cohort	1,833	SRQ20 (≥8)	< 6 weeks postpartum	aOR	5-21 months	1.53 (1.21, 1.92)	

Srivastava (2016)									
Bennett (2016)	India	Cohort	1,930	SRQ20 (≥8)	> 6 weeks postpartum	aRR	1 year	1.18 (1.03, 1.35)	1.11 (0.97, 1.26)
	India		1,930				5 years	1.07 (0.95, 1.22)	1.16 (1.00, 1.29)
	Peru		1,946				1 year	1.06 (0.93, 1.22)	0.85 (0.60, 1.19)
	Peru		1,946				5 years	0.96 (0.85, 1.09)	0.99 (0.67, 1.46)
	Vietnam		1,961				1 year	0.91 (0.81, 1.02)	1.29 (1.03, 1.62)
	Vietnam		1,961				5 years	1.12 (0.95, 1.33)	1.19 (0.95, 1.48)
	Ethiopia		1,885				1 year	0.91 (0.81, 1.02)	1.01 (0.89, 1.15)
	Ethiopia		1,885				5 years	0.94 (0.82, 1.09)	0.95 (0.80, 1.12)
Saeed (2017)	Pakistan	Cross-sectional	325	AKUADS (≥20)	12 months postpartum	aOR	< 2 years	3.15 (1.91, 5.18)	3.26 (1.99, 5.34)
Lima (2017)	Brazil	Cohort	1,124	CES-D (≥22)	2nd trimester	aOR	12-32 months	1.09 (0.62, 1.89)	0.84 (0.54, 1.31)
Wemakor (2018)	Ghana	Cross-sectional	200	CES-D (≥20)	6-23 months postpartum [c]	OR	6-23 months	1.05 (0.53, 1.73)	
Joshi and Raut (2019)	India	Cross-sectional	300	EPDS (≥10)	> 6 weeks postpartum	POR	< 1 year	1.49 (0.83, 2.65)	1.08 (0.59, 1.98)
Prado (2019)	Ghana	Cohort	1,003	EPDS[d]	6 months postpartum	OR[f]	18 months	0.73 (0.38, 1.39)	
Christodoulou (2019)[h]	South Africa	Cohort	470	EPDS (>13)	At birth	OR[g]	3 months	1.67	0.75
							6 months	0.50	0.00
							9 months	1.50	1.00
							12 months	1.13	1.00
							24 months	0.75	0.67
Ahmadi Gharaei (2020)	Iran	Cohort	470	DASS[e]	During pregnancy	aRR	6 months	1.10 (0.67, 1.81)	1.71 (1.07, 2.09)
Anato (2020)	Ethiopia	Cross-sectional	232	EPDS (≥13)	6 weeks postpartum	aOR	5-10 months	2.55 (1.24, 5.25)	
Tome (2020)	Zimbabwe	2ndary data analysis (cohort) of RCT	4,025	EPDS (≥12 or suicide)	During pregnancy	aOR	3 months	0.83 (0.53, 1.30)	
						aOR	6 months	0.82 (0.53, 1.28)	
						aOR	12 months	1.13 (0.84, 1.52)	
						aOR	18 months	1.10 (0.81, 1.49)	
Ricci (2023)	South Africa	2ndary data analysis (cross-sectional) of RCT	428	EPDS (>13)	6-9 months postpartum	aRR	6-9 months	1.02 (0.63, 1.66)	0.81 (0.41, 1.63)
Shriyan (2023)	India	Cohort	1,135	EPDS (>13)	During pregnancy and delivery within 48 h	aOR	1 year	1.72 (1.22, 2.43)	
Saleh (2023)	Tanzania	Cohort	1,739 for height 1,525 for	HSCL-8	During pregnancy	aRR	1 year	0.92 (0.83, 1.02)	0.80 (0.56, 1.16)

			weight						

a: For the studies that measured the outcomes repeatedly, we used the measure of association that was reported close to year 1 to prevent the correlation of repeated measurements.

b: Studies except for Brentani and Fink (2016) and Lima et al. (2017) used underweight as an outcome.

c: In the case of PPD and cumulative PPD, the timepoint of maternal depression measure was up until 24 months, which is beyond our inclusion criteria for PND.

d: A locally validated adapted version was used.

e: Authors reported the association between depression score in the DASS and the outcome.

f: The research team constructed an odds ratio and 95% CI based on raw data provided by the original study authors.

h: Studies excluded in the meta-analysis due to heterogeneity of measurement and no availability for CIs or SE.

i: Figs 2 and 3 from Nasreen et al. (2013) showed the mean HAZ and WAZ were lower among mothers with APD compared to mothers without APD, meaning that the negative coefficient actually predicts the increased risk rather than the protective effect.

Note: AKUADS = Aga Khan University Anxiety and Depression Scale, aOR = adjusted odds ratio, CES-D = Center for Epidemiological Studies-Depression, DASS = Depression Anxiety Stress Scales, EPDS = Edinburgh Postnatal Depression Scale, HSCL-8/-25 = Hopkins Symptom Checklist-8/-25, PDI = Pitt Depression Inventory, POR = prevalence odds ratio, RCT = randomized controlled trial, aRR = adjusted risk ratio, SCID for DSM-III-R, or -IV = Structured Clinical Interview for Diagnostic and Statistical Manual of Mental Disorders-III-R,

or -IV, SCAN = Schedules for Clinical Assessment in Neuropsychiatry, SE = standard error, SRQ20 = Self Reporting Questionnaire-20

Table 2. Adjusted covariates in each study included in the systematic review and meta-analysis.

Measure of Association	First author (year)	Adjusted variables
Odds ratio	Patel (2003)	Birthweight
	Rahman (2004, 1)	Birthweight, number of children, and socioeconomic status
	Rahman (2004, 2)	Low birthweight, breastfeeding, > 5 diarrheal episodes/year, parent education, maternal financial empowerment, relative poverty
	Anoop (2004)	Maternal intelligence, low birthweight, SES, duration of exclusive breastfeeding, duration of supplementary breastfeeding, immunization status, mother’s literacy
	Surkan (2008)	Child age, child gender, birthweight, breast-feeding duration, maternal education attainment, sanitation scale score, SES and living conditions scale score, number of children living in household, participation in Family Health Program, maternal self-efficacy score
	Madeghe (2016)	Maternal age, marital status, parity, income
	Upadhyay and Srivastava (2016)	Pregnancy intention, social supports, birth size, preterm birth, child sex, ever breastfed, serious illness, mother's age at birth of child, maternal education, maternal working status, antenatal care, iron folic acid tablets, tetanus injection, household head's education status, sex of household head, wealth index, drinking water, toilet facility, income socks, religion, caste, place of residence
	Saeed (2017)	Maternal age, education, income, occupation, child gender
	Lima (2017)	Monthly family income, maternal age, number of children, maternal schooling
	Anato (2020)	Wealth index, education, occupation, household food security, maternal workload
	Tome (2020)	Cluster, intervention arms, maternal at birth, mother’s height, mother’s mid upper arm circumference, child sex, single or multiple birth, household wealth index, presence of functioning hand washing station with water and soap, gender of household head, number of children under-fives, household dietary diversity, mother’s HIV status at enrolment
	Shriyan (2023)	Gestational age at delivery, age of the respondent, education, SES, parity, weight, height, gestational

		diabetes, birth weight, and smoking status of participants' husband.
Risk ratio	Avan (2010)	SES index, maternal age, child gender, preterm delivery
	Kaaya (2016)	Maternal perceived low social support, age, marital status, education, financial dependence, anemia, weight, height, HIV stage at enrolment and counselling session attendance, child's gender, birthweight, gestational age, breastfeeding, HIV status, vitamin A, multivitamin and other disease events.
	Bennett (2016)	Maternal schooling, sex of child, child age in months, wealth index
	Ahmadi Gharaei (2020)	Stunting: multivitamin intake, maternal education, birth height<br>Underweight: infant sex, having tooth at 6 months, birthweight, infection contraction within 6 months
	Ricci (2023)	Maternal age
	Saleh (2023)	Maternal age, maternal education, household wealth quintile, marital status, maternal employment status, parity, maternal CD4 category, maternal WHO HIV disease stage, taking ART, trial site, child sex, child age, child twin status, and randomized regimen
Beta-coefficient	Nasreen (2013)	Household SES, infant's sex, infant's weight at 2-3 months, infants' temperament (unadaptable)
	Brentani and Fink (2016)	Child age, child sex, twin, born small for gestational age, prematurity, maternal age, mother as a primary caregiver, caregiver primary education, caregiver secondary education, caregiver higher education, caregiver married, asset status

Note: ART = antiretroviral therapy, HIV = human immunodeficiency virus, SES = socioeconomic status

Primary outcomes

Stunting

Meta-analysis of ORs between PND and stunting

Children aged 6 to 32 months whose mothers had PND had significantly higher odds of stunting, OR 1.63 (n = 14, 95% CI 1.32, 2.02, I^2 = 56.0%), compared to those of mothers without PND (KH estimates: 1.66, 95% CI 1.29, 2.14, I^2 = 69.1%) (Fig 2). In a sensitivity

analysis that excluded studies with unadjusted ORs or PND measured up to 2 years produced a higher pooled estimate: OR 1.81 (n = 9, 95% CI 1.39, 2.36, I^2 = 62.3%). Further, when we limited the estimate to the recent publications (within the last 10 years) in this estimate, the overall effect remained substantial at 1.64 (n = 6, 95% CI 1.22, 2.19, I^2 = 69.4%).

In a subgroup analysis based on the type of PND (Fig 2A), APD and PPD were associated with 1.44 times the odds of stunting (n = 4, 95% CI 1.01, 2.06, I^2 = 60.5%), and 1.75 times the odds of stunting (n = 10, 95% CI 1.32, 2.31, I^2 = 54.5%), respectively (KH estimates: APD 1.46 (95% CI 0.97, 2.21, I^2 = 70.5%) and PPD 1.77 (95% CI 1.28, 2.43, I^2 = 65.8%). In a subgroup analysis by region (Fig 2B, 6 studies from Africa, 2 from America, 6 from Asia), studies conducted in Asia showed the greatest overall effect between PND and stunting (OR 1.95 (95% CI 1.49, 2.56), I^2 = 49.1%).

Fig 2. Meta-analysis of the odds ratio between perinatal depression and stunting (n = 14) – subgroup analysis according to region and type of perinatal depression (antepartum vs. postpartum).

Fig 2A. Subgroup analysis according to type of perinatal depression.

Fig 2B. Subgroup analysis according to region.

Fig 2 legend.

- The blue square and the horizontal line indicate ORs and their 95% confidence intervals, respectively.
- The red rhombus indicates the overall OR obtained from the DerSimonian and Laird method under a random-effects model.

When we stratified the estimates according to the timepoint of outcome measurement, stunting showed different patterns of association with APD and PPD during the first year (Fig

3). While the effect of APD was strongest when measured around 1 year postpartum, the effect of PPD was most pronounced when measured before 1 year. For both, the effect on stunting at each time point decreased after 1 year. In a subgroup analysis by study design (S1 Fig), cohort studies, which allow us to examine the temporal relationship between PPD and stunting (PPD occurring before the outcome of stunting) showed an attenuated effect of 1.55, in contrast to other study designs (n = 4, 95% CI 0.92, 2.63, I^2 = 62.4%).

Fig 3. Subgroup analysis of the odds ratio between perinatal depression and stunting according to the time point of stunting measurement.

Fig 3A. The odds ratio between antepartum depression and stunting according to the timepoint of outcome measurement.

Fig 3B. The odds ratio between antepartum depression and stunting according to the timepoint of outcome measurement.

Fig 3 legend.

- The blue square and the horizontal line indicate ORs and their 95% confidence intervals, respectively.
- The red rhombus indicates the overall OR obtained from the DerSimonian and Laird method under a random-effects model.
- The overall effect in the bottom of these figures should be ignored, given the repeated estimates of the same study.

Meta-analysis of RRs between PND and stunting

Children aged 6 to 24 months whose mothers had PND had similar risks of stunting, with a RR 0.99 (n of sub-studies = 10, 95% CI 0.90, 1.08, I^2 = 56.0%), compared to those of mothers without PND (KH estimates: 1.00 (95% CI 0.89, 1.13, I^2 = 74.4%). In the study by Kaaya et al. (2016), among the three estimates related to the timing of PND (APD, PPD, and

cumulative PPD), we selected the one measured for APD, that met the inclusion criteria of our study. A sensitivity analysis that excluded studies with unadjusted RRs or PND measured up until 2 years produced a similar pooled estimate: RR 0.99 (n = 9, 95% CI 0.90, 1.08, I^2 = 60.3%). Further, when we limited the analysis to the recent publications, the overall effect remained almost the same at 0.97 (n = 8, 95% CI 0.89, 1.06, I^2 = 54.7%).

The subgroup analyses based on the type of PND, region, timepoint of outcome measurement, and study design (Figs 4 and 5, and S2 Fig) showed consistently the null effects on stunting. The overall effect (RR) of APD on stunting at each time point showed a decreasing trend as the child ages (Fig 5A). However, it is important to note that this sub-analysis included only 3 studies.

Fig 4. Meta-analysis of the risk ratio between perinatal depression and stunting of children.

Fig 4A. Subgroup analysis according to type of perinatal depression.[a]

Fig 4B. Subgroup analysis according to region.

Fig 4 legend.

a: Bennett et al. (2016) was comprised of 4 different sub-studies, and Kaaya et al. (2016) reported the association with APD, PPD, and cumulative PPD separately.

- The blue square and the horizontal line indicate RRs and their 95% confidence intervals, respectively.
- The red rhombus indicates the overall RR obtained from the DerSimonian and Laird method under a random-effects model.
- The overall effect in the bottom of these figures should be ignored, given the repeated estimates of the same study and population (Kaaya et al. (2016)).

Fig 5. Subgroup analysis of the risk ratio between perinatal depression and stunting according to the time point of stunting measurement.

Fig 5A. The risk ratio between antepartum depression and stunting timepoint of outcome measurement.

Fig 5B. The risk ratio between postpartum depression and stunting according to the timepoint of outcome measurement.

Fig 5 legend.

- The blue square and the horizontal line indicate RRs and their 95% confidence intervals, respectively.
- The red rhombus indicates the overall RR obtained from the DerSimonian and Laird method under a random-effects model.
- The overall effect in the bottom of these figures should be ignored given the repeated estimates of the same study and population (Kaaya et al. (2016)).

Additional findings on the association between PND and stunting

Three additional studies were included in the systematic review. Nasreen et al. (2013), a cohort study in Bangladesh (Asia), reported that mothers with APD had statistically significantly higher adjusted probabilities of stunting among children aged 6-8 months than mothers without APD ("antepartum depressive symptoms predicted infant's stunting"). Brentani and Fink (2016), a cross-sectional study in Brazil, observed a slight increase in the adjusted probabilities of stunting among children aged 1 year from mothers with PPD compared to mothers without PPD; however, this difference was not statistically significant. Christodoulou et al. (2019), from a cohort study in South Africa, reported percentage proportions of stunting according to the presence of PND among mothers. In line with the subgroup analysis of OR based on the timing of stunting measurement, the percentage proportion estimates suggested that mothers with APD, measured at birth, had higher odds of stunting among children aged 1 year (OR 1.13) when compared to mothers without APD.

However, it was followed by protective odds of stunting among children aged 2 years (OR 0.75).

Underweight

Meta-analysis of ORs between PND and underweight

Children aged 6 to 24 months whose mothers had PND, exhibited significantly higher odds of underweight, with an OR of 2.65 (n = 10, 95% CI 1.90, 3.68, I^2 = 34.5%), when compared to children of mothers without PND (KH estimates: 2.67, 95% CI 1.86, 3.85, I^2 = 45.5%) (Fig 6). A sensitivity analysis that excluded studies with unadjusted ORs or PND measured up until 2 years yielded a higher pooled estimate: OR 3.25 (n = 7, 95% CI 2.41, 4.39, I^2 = 0.0%). Further, narrowing the range to the most recent publications resulted in only two studies being considered, with an overall OR of 3.67 (95% CI 2.33, 5.79, I^2 = 2.9%).

Fig 6. Meta-analysis of odds ratio between perinatal depression and underweight (n = 10) – subgroup analysis according to region and type of perinatal depression (antepartum vs. postpartum).

Fig 6A. Subgroup analysis according to type of perinatal depression.

Fig 6B. Subgroup analysis according to region.

Fig 6 legend.

- The blue square and the horizontal line indicate ORs and their 95% confidence intervals, respectively.
- The red rhombus indicates the overall OR obtained from the DerSimonian and Laird method under a random-effects model.

The subgroup analysis comparing underweight and APD versus PPD showed a similar trend. Mothers with PPD had an OR 2.62 (n = 9, 95% CI 1.79, 3.83, I^2 = 40.9%) for having an

underweight child than mothers without PPD, while mothers with APD had three times the odds (n = 1, 95% CI 1.50, 6.00) of having an underweight child compared to mothers without APD. In the subgroup analysis based on region, although the number of included studies was small, the magnitude of the overall OR between PND and underweight followed the order of Africa (n = 3) > Asia (n = 6) > America (n = 1).

When we stratified the estimates according to the timepoint of outcome measurement, the overall association between PPD and underweight was the highest at the earliest time point (4 months) (n = 1), followed by the decreasing patterns (Fig. 7). Additionally, the overall effect of PPD on underweight among cohort studies (n = 2, OR 2.55, 95% CI 1.30, 4.98, I^2 = 0.0%) demonstrated a similar magnitude to the overall effect of PPD (S3 Fig). Unfortunately, we could not conduct a subgroup analysis with APD since only one study examined the association between APD and underweight (n = 1).

Fig 7. Meta-analysis of the odds ratio between postpartum depression and underweight – subgroup analysis according to the time point of underweight measurement (n = 9).

Fig 7 legend.

- The blue square and the horizontal line indicate ORs and their 95% confidence intervals, respectively.
- The red rhombus indicates the overall OR obtained from the DerSimonian and Laird method under a random-effects model.

Meta-analysis of RRs between PND and underweight

Children aged 6 to 24 months whose mothers had PND had a similar risk of underweight, with a RR of 1.06 (n of sub-studies = 10, 95% CI 0.93, 1.22, I^2 = 49.5%),

compared to those of mothers without PND (KH estimates: 1.05, 95% CI 0.88, 1.235 I^2 = 70.0%). In Kaaya et al. (2016), we selected the estimate for APD that met our inclusion criteria. A sensitivity analysis, which excluded studies with unadjusted RRs or PND measured up to 2 years, produced a similar pooled estimate: RR 1.06 (n = 9, 95% CI 0.92, 1.22, I^2 = 54.6%). When we further limited the analysis to the recent publications, the overall effect RR remained at 1.07 (n = 7, 95% CI 0.93, 1.46, I^2 = 57.8%).

The subgroup analysis comparing underweight and APD versus PPD yielded the results that closely resembled the overall analysis (Fig 8A). In the subgroup analysis based on the region (Fig 8B), although the numbers of included studies were limited, the effect direction varied by region. In America, one study indicated a potentially protective effect on child underweight among mothers with PND compared to those without PND, though this finding was not statistically significant. Conversely, the overall effect in Asia (RR 1.16 (95% CI 1.01, 1.33)), Africa (RR 1.02 (95% CI 1.00, 1.03)) and one study from the Middle East suggested a higher risk of child underweight among mothers with PND compared to their counterparts.

Fig 8. Meta-analysis of the risk ratio between perinatal depression and underweight of children.

Fig 8A. Subgroup analysis according to type of perinatal depression.[a]

Fig 8B. Subgroup analysis according to region.

Fig 8 legend.

a: Bennett et al. (2016) was comprised of 4 different sub-studies, and Kaaya et al. (2016) reported the association with APD, PPD, and cumulative PPD separately.

- The blue square and the horizontal line indicate RRs and their 95% confidence intervals, respectively.

- The red rhombus indicates the overall RR obtained from the DerSimonian and Laird method under a random-effects model.
- The overall effect in the bottom of these figures should be ignored, given the repeated estimates of the same study and population (Kaaya et al. (2016)).

When we stratified the estimates based on the timepoint of outcome measurement, the association between APD and underweight was stronger at the earliest time point (n = 1, 4 months) compared to those obtained in 1 year (n = 1) and 2 years (n = 1) (Fig. 9). The association between PPD and underweight showed a more consistent pattern; while none of the subgroup analyses were statistically significant, the direction of the effect changed from being greater than 1 at year 1 to being less than 1 at year 2, followed by returning to a value greater than 1 at year 5. Additionally, the overall effect of PPD on underweight among cohort studies (n of sub-studies= 6, RR 1.02, 95% CI 0.90, 1.16, I^2 = 48.3%) yielded a similar magnitude to the overall effect of PPD (S4 Fig).

Fig 9. Subgroup analysis of the risk ratio between perinatal depression and underweight according to the time point of stunting measurement.

Fig 9A. The risk ratio between antepartum depression and underweight according to the timepoint of outcome measurement.

Figure 9B. The risk ratio between postpartum depression and underweight according to the timepoint of outcome measurement.

Fig 9 legend.

- The blue square and the horizontal line indicate ORs and their 95% confidence intervals, respectively.
- The red rhombus indicates the overall OR obtained from the DerSimonian and Laird method under a random-effects model.

- The overall effect in the bottom of these figures should be ignored, given the repeated estimates of the same study.

Additional findings on the association between PND and underweight

Three additional studies were included in the systematic review. Nasreen et al. (2013) reported statistically significantly higher adjusted probabilities of underweight among children aged 6-8 months ("Maternal postpartum depressive symptoms independently predicted infant's underweight").(71) Christodoulou et al. (2019) presented percentage proportions suggesting a null or protective effect of APD measured at birth on underweight over a 2-year periods following birth.

Wasting

Five studies that met the inclusion criteria reported wasting with heterogenous measures of association (38,79,81,87,91) (Table 3). Joshi and Raut (2019) in India reported an almost null prevalence OR for wasting among infants aged less than one year when comparing mothers with and without PPD (prevalence OR=1.01, 95% CI 0.51, 2.01). In South Africa, Christodoulou showed percentage proportions indicating variations in wasting among mothers with and without APD over a 2-year period.

Table 3. Summary of the study characteristics included in the systematic review and meta-analysis for the association between perinatal depression and wasting of children in low- and middle-income countries from 2003 to 2023.

First author (year)	Country	Study design	Sample size	Depression measure (cutoff)	Time of depression measure	Association measure	Time of outcome measure (after-	Wasting (95% CI)

							birth)	
Avan (2010)	South Africa	Cohort	892	PDI (≥19)	> 6 weeks postpartum	aRR	24 months	0.66 (0.30, 1.42)
Kaaya (2016)	Tanzania	Cohort	699	HSCL-25	During pregnancy	aRR	< 2 years	1.06 (0.74, 1.51)
					<2 years postpartum[a]			0.59 (0.35, 1.01).
					Cumulative postpartum			1.08 (1.04, 1.12)
Joshi and Raut (2019)	India	Cross-sectional	300	EPDS (≥10)	> 6 weeks postpartum	POR	< 1 year	1.01 (0.51, 2.01)
Christodoulou (2019)[c]	South Africa	Cohort	470	EPDS (>13)	At birth	OR[b]	3 months	1.45
							6 months	1.50
							9 months	0.00
							12 months	0.50
							24 months	2.00
Saleh (2023)	Tanzania	Cohort	1,739 for height 1,525 for weight	HSCL-8	During pregnancy	aRR	1 year	2.61 (1.03, 6.65)

a: In case of PPD and cumulative PPD, the timepoint of maternal depression measure was until 24 months, which is beyond of our inclusion criteria for PND.

b: The authors reported the risk of each stratum of exposure and outcome, and the research team calculated ORs. Since the CIs were not presented, these estimates are only included in the systematic review.

c: Studies excluded in the meta-analysis due to heterogeneity of measurement and no availability for CIs or SE.

Note: CIs = confidence intervals OR = odds ratio, EPDS = Edinburgh Postnatal Depression Scale, HSCL-25 = Hopkins Symptom Checklist, PDI = Pitt Depression Inventory, POR = prevalence odds ratio, aRR = adjusted risk ratio, SE = standard error

Kaaya et al. conducted in Tanzania examined the association of wasting with three exposures: APD, PPD, and cumulative PPD. Among these, only cumulative PND showed statistically a significant association with wasting among mothers with HIV (aRR 1.08 (95% CI 1.04, 1.12)). The aRR of APD 1.06 (95% CI 0.74, 1.51) and for PPD, it was 0.59 (95% CI 0.35, 1.01). A meta-analysis of RRs from Avan et al. (2010), Kaaya et al. (2016), and Saleh et al. (2023), three cohort studies from Africa, suggested contrasting effects of APD and PPD on the risk of wasting. The RR of APD on wasting was 1.49 (95% CI 0.63, 3.52, I^2 = 68.0%) (KH estimates: 1.48, 95% CI 0.65, 3.38, I^2 = 65.6%), while the RR of PPD on wasting was 0.81 (95% CI 0.51, 1.29, I^2 = 69.2%) (KH estimates: 0.84, 95% CI 0.57, 1.24, I^2 = 58.4%) (Fig 10). Saleh et al. (2023) showed a stronger effect of APD on the risk of wasting at year 1 compared to Avan et al. (2010) and Kaaya et al. (2016), suggesting the heterogenous effects of PND on the risk of wasting (S5 Fig).

Fig 10. Subgroup analysis of the risk ratio between perinatal depression and wasting according to type of perinatal depression.[a]

Fig 10 legend.

- The blue square and the horizontal line indicate RRs and their 95% confidence intervals, respectively.
- The red rhombus indicates the overall RR obtained from the DerSimonian and Laird method under a random-effects model.

Overweight/Obesity

Only two studies reported overweight or obesity as an outcome. Lima et al. (2017), a cohort study in Brazil, found that mothers with and without APD had an OR of 0.84 for

overweight among children aged 12 to 32 months (95% CI 0.54, 1.31), suggesting a potential protective effect of APD on overweight. On the contrary, Brentani and Fink (2016), a cross-sectional study in Brazil, reported a slight increase in the adjusted probabilities of obesity among children aged 1 year among mothers who had PPD compared to those without PPD, a potential risk of PPD on obesity.(60) However, both were not statistically significant.

Meta-regression

To investigate the source of heterogeneity, we conducted the meta-regression analyses using the Sidik–Jonkman random-effects method for each association (RRs and ORs) between PND and outcomes (stunting and underweight). We considered several variables, including using the type of PND, study year (categorical variable), income level (low income, low middle income, high middle income), region, country, study design, and months of outcome measured (continuous variable). When examining the OR between PND and stunting, country explained the largest proportion of between-study variance (R^2 74.0%). For the RR between PND and stunting, study year emerged as a significant predictor (R^2 34.9%, p-value for the year 2010 = 0.039) (Table 4). Furthermore, the region of Africa (p-value = 0.046) and the secondary analysis of RCT (p-value = 0.010) were found to be statistically significant predictors for the OR between PND and stunting. For the OR between PND and underweight, study year explained the highest proportions of the between-study variance (R^2 56.7%, p-value for 2019 = 0.058). For the RR between PND and underweight, country, region and study year played significant roles (R^2 of country 79.5%, p-value for Iran = 0.003, p-value for Vietnam = 0.025, R^2 of region 49.8%, p-value for Africa = 0.011 and R^2 of year 42.9%, p-value for year 2020 = 0.008).

Table 4. Table of estimates from the meta-regression.

Association	Variable	Test of residual homogeneity	I^2 (%)	R^2 (%)

		Q residual	p-value		
Log OR between PND and stunting	APD vs. PPD	40.65	0.004	66.8	11.2
	Study year	21.15	0.048	60.2	24.6
	Income level	41.42	0.002	70.3	0.0
	Region	35.46	0.012	66.4	10.9
	Country	8.05	0.887	37.9	74.7
	Study design	26.53	0.088	60.8	31.5
	Months of outcome measured	50.52	0.000	71.5	0.0
Log OR between PND and underweight	APD vs. PPD	14.63	0.331	31.5	0.0
	Study year	2.43	0.965	12.8	56.7
	Income level	14.19	0.289	32.9	0.0
	Region	13.54	0.331	32.2	0.0
	Country	8.25	0.509	31.17	0.0
	Study design	13.38	0.342	30.6	0.0
	Months of outcome measured	14.84	0.318	30.3	0.0
Log RR between PND and stunting	APD vs. PPD	25.95	0.026	78.6	3.7
	Study year	24.76	0.016	74.1	34.9
	Income level	27.40	0.011	77.5	1.2
	Region	32.16	0.001	78.7	0.0
	Country	20.30	0.009	73.2	20.1
	Study design	32.79	0.003	82.4	0.0
	Months of outcome measured	32.63	0.003	81.5	0.0
Log RR between PND and underweight	APD vs. PPD	29.43	0.009	83.7	0.0
	Study year	18.79	0.093	76.1	42.9
	Income level	29.51	0.006	82.1	0.0

	Region	8.27	0.764	70.7	49.8
	Country	5.71	0.680	34.6	79.5
	Study design	30.20	0.007	84.2	0.0
	Months of outcome measured	28.82	0.011	81.7	0.0

Publication Bias

Visual inspection of the funnel plot reveals some asymmetries in the pooled associations found in this study, suggesting the presence of publication bias (Fig 11). However, it is worth noting that despite the limited number of included studies, no statistically significant publication bias was detected when applying Egger's tests. These studies reported OR of stunting (n = 14, p=0.241), RR of stunting (n of sub-studies = 10, p=0.292), OR of underweight (n = 10, p=0.403), and RR of underweight (n of sub-studies = 10, p=0.685).

Fig 11. Contour-enhanced Funnel plots of included studies in the meta-analysis.

A. **A funnel plot of studies reporting odds ratios between perinatal depression and stunting.**
B. **A funnel plot of studies reporting odds ratios between perinatal depression and underweight.**
C. **A funnel plot of studies reporting risk ratios between perinatal depression and stunting.**
D. **A funnel plot of studies reporting risk ratios between perinatal depression and underweight.**

Discussion

This systematic review and meta-analysis included 27 studies, comprising a total of 27 sub-studies, spanning the period from 2003 to 2023 in LMICs. It explored various adverse child growth outcomes and their relationship with PND. The association between PND and adverse growth outcomes exhibited variations based on the measure of association, country/region, types of PND, and study design. Among the studies reporting ORs, PND was significantly associated to stunting (OR = 1.63, 95% CI 1.32, 2.02) and underweight (OR=2.65, 95% CI 1.90, 3.68) in children up to 32 months of age. The meta-analysis, pooling RRs for stunting, underweight, and wasting, did not reveal a significant association, as mirroring the findings with ORs. Unfortunately, we could not conduct a meta-analysis for obesity/overweight due to the limited numbers of relevant studies.

PND has been associated with intrauterine growth restriction, premature delivery, and low birth weight.(92) Biologic mechanisms such as increased stress hormones due to depression that are transmitted in utero may also influence children's nutritional outcomes.(93) PPD, through its impacts on mood, concentration and cognition, energy levels, and other factors, can impact caregiving abilities, undermine breastfeeding outcomes, and impede mother-baby bonding.(6,94) Women suffering from depression, on average, also have higher risks for engaging in harmful behaviors, such as drinking or smoking, that may negatively impact their children.(95)

In addition to genetic and hormonal determinants, environmental factors like social support and economic status play an important role in LMICs in the onset of PND as well as in child health outcomes.(96) These include stress, trauma, and the physical and emotional demands of pregnancy, delivery, and child care as additional potential triggers for PND that may be experienced more frequently or more acutely by women in LMICs who lack support or

coping resources.(5) Unfortunately, mental health is underprioritized and underfunded, especially in most LMICs. Up to 85% of people with severe mental disorders in LMICs receive no treatment, while average per capita spending on mental health is just US $0.25.(97) Despite the existence of numerous screening and diagnostic tools, many of which have been adapted to LMIC contexts, routine screening for PND is not common in many LMICs, especially in more rural and low-resource areas. Therefore, PND may be substantially underreported and undertreated in LMICs.(7)

Currently, there are limited numbers of studies for each outcome, and we have observed significant variations in the characteristics of existing studies, including different measures of associations, features of the study population (country, region, time), and analytic approaches (selection of confounding factors). As revealed in subgroup analyses and meta-regression, country and region are key factors that explained the heterogeneity in the existing studies. Studies conducted in India and Pakistan consistently report statistically significantly increases in adjusted measures of association for stunting among children who born to mothers with PND when compared to children of mothers without PND.(62,72,83,98) India and Pakistan have historically had some of the highest rates of regions with the highest rates of stunting (more than 30%) among children for decades, and women in these regions often experience excessive workloads and high stress levels, particularly during perinatal periods.(13,99) Moreover, this relationship was also observed in a study focusing on child growth outcomes. Holm-Larsen et al. (2019) conducted the cohort study in Kenya, where the prevalence of stunting was high (26.2% during the study period) (S2 Table).(13) They reported that the mean HAZ of children aged 2-3 years, whose mothers had PPD, was significantly lower by 0.32 units (95% CI -0.49, -0.15) compared to those whose mothers did not have PPD.(32) However, Santos et al. (2010) conducted a study in Brazil, where the prevalence of stunting was much lower at 3.6% among the study population, they reported no association

between maternal PPD and stunting among 4-year-old children after adjusting for confounding factors (S2 Table).(48)

Further, a notable gap in the existing research pertains to the assessment of the degree of support system, family structure, the extent of a mother's influence on child growth, and food insecurity. The unique characteristics and dynamics of LMIs, shaped by their demographic transitions, imply that the role of the community and support systems in childcare can vary significantly. In some studies, rather ironically, PND has been identified as a protective factor against adverse growth outcomes in children. Consequently, children whose mothers experience PND, and the resulting challenges in caregiving, may be at a heightened risk of stunting, a chronic form of malnutrition, especially if they have limited access to food sources and reside in regions where stunting is more prevalent.

We observed that a combined exposure to PND elevated the odds of stunting throughout the initial 32 months of a child's life by 63%, while the influence of PPD alone was even more pronounced, increasing the odds of stunting by 75%. Stunting is unique in its association with fetal development during pregnancy, setting it apart from other forms of undernutrition. This condition is intertwined with both to woman's nutritional status during pregnancy as well as the baby's nutritional status after birth and can be largely prevented through optimal maternal health and child feeding and care practices during the first 1,000 days.(8)

PND exhibited an even more pronounced effect on children being underweight with an OR of 2.73 (95% CI 1.92, 3.91), although this was primarily driven by the effect of PPD. Underweight is often an indicative of insufficient nutrient intake and can result from a multitude of environmental and genetic factors. In alignment with the findings on stunting, studies

conducted in India and Pakistan consistently reported elevated risks of underweight among children whose mothers with PND compared to those without PND.(72,74,75,84,98) Furthermore, studies from Iran and Vietnam also found a statistically significant association between underweight and PND, even though they could not observe a similar association between stunting and PND.(62,88)

Wasting, which heightens the risk of death in young children due to recent, severe weight loss, is a relatively rare outcome with a global prevalence of 6.7% compared to stunting at 22% among children under the age of five.(13,100) The two studies meeting our criteria resulted inconclusive associations, even with the qualitative analysis.(79,87) Results from the studies excluded due to the heterogeneity of measures of association also presented conflicting findings. For example, in a cohort study in Tanzania (N = 1,128), the adjusted mean z-score of weight for height was significantly 0.21 units higher (95% CI 0.02, 0.40)) among 2 to 3-year-old children with mothers with PPD compared to those without PPD, suggesting that PPD maybe protective against wasting.(32) In the Brazilian cohort study (N = 3,564), which we excluded due to timing of outcome measurement, the authors reported that mothers who scored as having PPD three times (12, 24, and 48 months).(48) Although not statistically significant, the adjusted odds for wasting of children aged 48 months was 1.5 times higher among mothers with PPD compared to mothers without PPD (95% CI 0.4, 7.6). However, when mothers who did not meet the PPD criteria all three points were compared to the mothers without PPD, aOR was 0.6 (95% CI 0.2-2.0). Given that wasting is an acute outcome of malnutrition, it may be more influenced by acute food crises, such as natural and man-made disasters, rather than maternal depression-related dysfunction. Additionally, in some LMICs, where the traditional extended family system remains prevalent, the risk of children experiencing wasting might be mitigated by the support of extended family members.(99)

Consequently, further data is needed to draw definitive conclusions regarding the impact of PND on wasting in LMICs.

In light of the growing worldwide obesity epidemic, there is emerging evidence that PND may be associated with childhood overweight and obesity, although research in LMICs remains limited.(101–103) The mechanisms of child overweight and obesity are not fully understood but are believed to include a complex interplay of genetic, lifestyle, and environmental factors.(104) In LMICs which face the challenge of a dual burden of malnutrition, overweight was initially associated with higher SES segments of society, while underweight was more common among the poor. However, as energy-dense, nutrient-poor diets become increasingly prevalent among lower SES groups, the prevalence of overweight has also risen.(105) Similar to the issue of with wasting, we observed that only one study on overweight/obesity met the criteria for analysis, making it difficult to discern clear patterns. In addition to Lima et al. (2017), Santos et al. reported no association between PPD and overweight among children aged four years after adjusting for confounding factors when mothers who had PPD once or twice over three times of PPD measurement during the four-year measurement period.(48) However, when mothers experienced PPD three times were compared to mothers without PPD, four-year-old children whose mothers had PPD had 1.6 times the adjusted odds of being overweight (95% CI 1.0, 2.5) compared to those whose mothers were without PPD. Consequently, further research is needed to investigate the relationship between PND and overweight/obesity among children in LMICs.

This study effectively addressed several limitations present in the most recently published meta-analysis.(23) First, instead of pooling the association between maternal depression and adverse child growth outcomes regardless of the temporal relationship between them, we prioritize investigating the impact of PND on those outcomes by including

the studies where outcomes occurred after or at least at the same time with the PND. Secondly, we extended the analysis to include studies published over a longer time interval, spanning from 2003 to 2023. We expanded the scope by examining a wider array of databases, including five Latin American platforms, which have typically been underrepresented in meta-analyses on this topic. In contrast to the approach of Dadi et al., where multiple adverse child growth outcomes, such as underweight, wasting, and short stature, were combined into a pooled estimate termed "malnutrition," with a reported pooled RR of 1.39 (95% CI 1.21, 1.61), we analyzed the composite adverse child growth outcomes separately because they have distinct etiologies. We also addressed the challenge of heterogeneity in outcome measurement time by conducting analyses on children aged approximately 12 months, with an age range from 6 to 32 months. Lastly, sought to explore the impact of the two types of PND, namely, APD and PPD, by considering their differential onset.

The collection, definition, and reporting of covariates exhibited significant heterogeneity across the included studies. Furthermore, the interpretation of the publication analysis was constrained due to the limited number of included studies for the homogenous research question. It is worth noting that more than half of the studies, which performed adjusted analyses, incorporated birthweight or preterm birth as covariates (S6 Fig). It is important to acknowledge that untreated APD is a strong predictor for PPD,(106) as roughly one-third of PPD cases have onset during pregnancy, and 27% start prior to pregnancy.(107) Thus, adjusting for these factors may inadvertently block one of the causal pathways we aim to measure. Additionally, it is essential to consider that there are often variable lags between depression onset and its clinical diagnosis. A postpartum diagnosis, for instance, does not conclusively rule out the presence of depression during or prior to pregnancy. Therefore,

conducting further longitudinal cohort studies that can account for these time-varying characteristics of exposure, outcome, and confounding factors is imperative.

Conclusion

A limited numbers of studies have investigated the association between PND and adverse child growth outcomes in LMICs. Among these studies, we observed a significant association between PND and an increased odds of stunting and underweight status in children. These findings underscore the long-term implications for child health and development and emphasize the disproportionate impact on children in resource-constrained settings. Despite availability of evidence for supporting low-resource intervention programs, such as the WHO-recommended Thinking Healthy Program in LMICs, there remains a substantial gap in their implementation and scale-up.(108) As eloquently articulated by Howard et al. (2014), 'There is no health without perinatal mental health.'(109) It is imperative for public health practitioners to prioritize the prevention, screening, and treatment of both prenatal and postpartum depression as integral components of efforts to combat child malnutrition in LMICs, within the framework of as broader maternal and child health programs.

Acknowledgments

The authors thank Dr. Paul Bain, a librarian of the Harvard Countway Library, for his contributions to the search strategy. We are also grateful for Dr. Catie Oldenburg, the professor of the Department of Epidemiology & Biostatistics at University of California San Francisco for her advice on the revision of this manuscript.

References

1. Woody CA, Ferrari AJ, Siskind DJ, Whiteford HA, Harris MG. A systematic review and meta-regression of the prevalence and incidence of perinatal depression. J Affect Disord. 2017 Sep 1;219:86–92.

2. Shidhaye P, Giri P. Maternal depression: a hidden burden in developing countries. Ann Med Health Sci Res. 2014 Jul;4(4):463–5.

3. World Health Organization, Fund UNP. Mental health aspects of women's reproductive health : a global review of the literature [Internet]. World Health Organization; 2009 [cited 2021 Nov 2]. Available from: https://apps.who.int/iris/handle/10665/43846

4. Stewart DE, Vigod S. Postpartum Depression. N Engl J Med. 2016;375(22):2177–86.

5. The National Institute of Mental Health. National Institute of Mental Health. [cited 2021 Nov 2]. Perinatal Depression. Available from: https://www.nimh.nih.gov/health/publications/perinatal-depression

6. Parsons CE, Young KS, Rochat TJ, Kringelbach MortenL, Stein A. Postnatal depression and its effects on child development: a review of evidence from low- and middle-income countries. Br Med Bull. 2012 Mar 1;101(1):57–79.

7. Gelaye B, Rondon MB, Araya R, Williams MA. Epidemiology of maternal depression, risk factors, and child outcomes in low-income and middle-income countries. Lancet Psychiatry. 2016 Oct;3(10):973–82.

8. Scott JA. The first 1000 days: A critical period of nutritional opportunity and vulnerability. Nutr Diet J Dietit Assoc Aust. 2020 Jul;77(3):295–7.

9. Patel V, Saxena S, Lund C, Thornicroft G, Baingana F, Bolton P, et al. The Lancet Commission on global mental health and sustainable development. Lancet Br Ed. 2018;392(10157):1553–98.

10. Dadi AF, Akalu TY, Wolde HF, Baraki AG. Effect of perinatal depression on birth and infant health outcomes: a systematic review and meta-analysis of observational studies from Africa. Arch Public Health. 2022 Jan 20;80(1):34.

11. Kwon EJ, Kim YJ. What is fetal programming?: a lifetime health is under the control of in utero health. Obstet Gynecol Sci. 2017 Nov;60(6):506–19.

12. Black RE, Victora CG, Walker SP, Bhutta ZA, Christian P, de Onis M, et al. Maternal and child undernutrition and overweight in low-income and middle-income countries. Lancet Lond Engl. 2013 Aug 3;382(9890):427–51.

13. United Nations Children's Fund (UNICEF), World Health Organization, International Bank, for Reconstruction and Development/The World Bank. Levels and trends in child malnutrition: key findings of the 2021 edition of the joint child malnutrition estimates. Geneva: World Health Organization; 2021.

14. WHO. World Health Organization. 2021 [cited 2021 Nov 29]. Obesity and overweight. Available from: https://www.who.int/news-room/fact-sheets/detail/obesity-and-overweight

15. Baron EC, Hanlon C, Mall S, Honikman S, Breuer E, Kathree T, et al. Maternal mental health in primary care in five low- and middle-income countries: a situational analysis. BMC Health Serv Res. 2016 Feb 16;16(1):53.

16. Headey D, Heidkamp R, Osendarp S, Ruel M, Scott N, Black R, et al. Impacts of COVID-19 on childhood malnutrition and nutrition-related mortality. The Lancet. 2020 Aug 22;396(10250):519–21.

17. Mertens E, Peñalvo JL. The Burden of Malnutrition and Fatal COVID-19: A Global Burden of Disease Analysis. Front Nutr. 2020;7:619850.

18. Martin. Goal 3: Ensure healthy lives and promote well-being for all at all ages [Internet]. United Nations Sustainable Development. 2021 [cited 2021 Nov 2]. Available from: https://www.un.org/sustainabledevelopment/health/

19. Liu Y, Kaaya S, Chai J, McCoy DC, Surkan PJ, Black MM, et al. Maternal depressive symptoms and early childhood cognitive development: a meta-analysis. Psychol Med. 2017 Mar;47(4):680–9.

20. Burger M, Hoosain M, Einspieler C, Unger M, Niehaus D. Maternal perinatal mental health and infant and toddler neurodevelopment - Evidence from low and middle-income countries. A systematic review. J Affect Disord. 2020 May 1;268:158–72.

21. WHO. Children: improving survival and well-being [Internet]. [cited 2021 Nov 2]. Available from: https://www.who.int/news-room/fact-sheets/detail/children-reducing-mortality

22. Pelletier DL, Frongillo EA, Schroeder DG, Habicht JP. The effects of malnutrition on child mortality in developing countries. Bull World Health Organ. 1995;73(4):443–8.

23. Dadi AF, Miller ER, Mwanri L. Postnatal depression and its association with adverse infant health outcomes in low-and middle-income countries: A systematic review and meta-analysis. BMC Pregnancy Childbirth [Internet]. 2020;20(1). Available from: https://www.embase.com/search/results?subaction=viewrecord&id=L632408992&from=export

24. Lampard AM, Franckle RL, Davison KK. Maternal depression and childhood obesity: a systematic review. Prev Med. 2014 Feb;59:60–7.

25. Marco PL, Valerio ID, de Mola Zanatti CL, Goncalves H. Systematic review: Symptoms of parental depression and anxiety and offspring overweight. Rev Saude Publica. 2020;54:49.

26. Surkan PJ, Kennedy CE, Hurley KM, Black MM. Maternal depression and early childhood growth in developing countries: systematic review and meta-analysis. Bull World Health Organ. 2011 Aug 1;89(8):608–15.

27. Page MJ, McKenzie JE, Bossuyt PM, Boutron I, Hoffmann TC, Mulrow CD, et al. The PRISMA 2020 statement: an updated guideline for reporting systematic reviews. BMJ Online. 2021;372:n71–n71.

28. World Health Organization. WHO Child Growth Standards: length/height-for-age, weight-for-age, weight-for-length, weight-for-height and body mass index-for-age: methods and development. WHO Press; 2006.

29. Covidence systematic review software. Melbourne, Australia: Veritas Health Innovation;

30. Country Classification – World Bank Data Help Desk [Internet]. [cited 2021 Nov 2]. Available from: https://datahelpdesk.worldbank.org/knowledgebase/topics/19280-country-classification

31. Rotheram-Fuller EJ, Tomlinson M, Scheffler A, Weichle TW, Hayati Rezvan P, Comulada WS, et al. Maternal patterns of antenatal and postnatal depressed mood and the impact on child health at 3-years postpartum. J Consult Clin Psychol. 2018 Mar;86(3):218–30.

32. Holm-Larsen CE, Madsen FK, Rogathi JJ, Manongi R, Mushi D, Meyrowitsch DW, et al. Postpartum depression and child growth in Tanzania: a cohort study. BJOG Int J Obstet Gynaecol. 2019 Apr;126(5):590–8.

33. Gordon S, Rotheram-Fuller E, Rezvan P, Stewart J, Christodoulou J, Tomlinson M. Maternal depressed mood and child development over the first five years of life in South Africa. J Affect Disord. 2021 Nov 1;294:346–56.

34. Li X, Gao R, Dai X, Liu H, Zhang J, Liu X, et al. The association between symptoms of depression during pregnancy and low birth weight: a prospective study. Bmc Pregnancy Childbirth. 2020 Mar 6;20(1):147.

35. Choi H, Lim JY, Lim NK, Ryu HM, Kwak DW, Chung JH, et al. Impact of pre-pregnancy body mass index and gestational weight gain on the risk of maternal and infant pregnancy complications in Korean women. Int J Obes 2005. 2022 Jan;46(1):59–67.

36. Wu S, Gennings C, Wright RJ, Wilson A, Burris HH, Just AC, et al. Prenatal stress, methylation in inflammation-related genes, and adiposity measures in early childhood: The Programming Research in Obesity, Growth Environment and Social Stress cohort study. Psychosom Med. 2018 Jan;80(1):34–41.

37. Wang H, Zhou H, Zhang Y, Wang Y, Sun J. Association of maternal depression with dietary intake, growth, and development of preterm infants: a cohort study in Beijing, China. Front Med. 2018 Oct;12(5):533–41.

38. Christodoulou J, Le Roux K, Tomlinson M, Le Roux IM, Katzen LS, Rotheram-Borus MJ. Perinatal maternal depression in rural South Africa: Child outcomes over the first two years. J Affect Disord. 2019 Mar 15;247:168–74.

39. Tome J, Mbuya MNN, Makasi RR, Ntozini R, Prendergast AJ, Dickin KL, et al. Maternal caregiving capabilities are associated with child linear growth in rural Zimbabwe. Matern Child Nutr. 2021 Apr;17(2):e13122.

40. Odendaal HJ, Human M, van der Merwe C, Brink LT, Nel DG, Goldstein RD. The Association between Maternal Depression, Infant Characteristics and Need for Assistance in A Low-Income-Country. J Subst Abuse Alcohol. 2021;8(2):1090.

41. Harper A, Rothberg A, Chirwa E, Mall S. Examining maternal depression, birthweight and linear growth: Findings from the South African National Income Dynamics Study. SAJCH South Afr J Child Health. 2022;16(4):215–9.

42. Apriliana T, Keliat BA, Mustikasari M, Primasari Y. A contributing factor of maternal pregnancy depression in the occurrence of stunting on toddlers. J Public Health Res. 2021 Dec 2;11(2).

43. Shriyan P, Khetrapal S, van Schayck OCP, Babu GR. Maternal depressiveness and infant growth outcomes: Findings from the MAASTHI cohort study in India. J Psychosom Res. 2023 Jul;170:111378.

44. Kaiyo-Utete M, Langhaug L, Chingono A, Dambi JM, Magwali T, Henderson C, et al. Antenatal depression: Associations with birth and neonatal outcomes among women attending maternity care in Harare, Zimbabwe. PloS One. 2023;18(7):e0270873.

45. Baker-Henningham H, Powell C, Walker S, Grantham-McGregor S. Mothers of undernourished Jamaican children have poorer psychosocial functioning and this is associated with stimulation provided in the home. Eur J Clin Nutr. 2003;57(6):786–92.

46. Surkan PJ, Ryan LM, Carvalho Vieira LM, Berkman LF, Peterson KE. Maternal social and pyschological conditions and physical growth in low-income children in Piauí, Northeast Brazil. Soc Sci Med 1982. 2007 Jan;64(2):375–88.

47. Surkan PJ, Kawachi I, Peterson KE. Childhood overweight and maternal depressive symptoms. J Epidemiol Community Health. 2008 May;62(5):e11.

48. Santos IS, Matijasevich A, Domingues MR, Barros AJD, Barros FCF. Long-lasting maternal depression and child growth at 4 years of age: a cohort study. J Pediatr. 2010 Sep;157(3):401–6.

49. Hassan BK, Werneck GL, Hasselmann MH. Maternal mental health and nutritional status of six-month-old infants. Rev Saúde Pública Online. 2016;50:7–7.

50. Motlhatlhedi K, Setlhare V, Ganiyu AB, Firth JA. Association between depression in carers and malnutrition in children aged 6 months to 5 years. Afr J Prim Health Care Fam Med. 2017;9(1):a1270.

51. Choi KW, Sikkema KJ, Vythilingum B, Geerts L, Faure SC, Watt MH, et al. Maternal childhood trauma, postpartum depression, and infant outcomes: Avoidant affective processing as a potential mechanism. J Affect Disord. 2017 Mar 15;211:107–15.

52. Neamah HH, Sudfeld C, McCoy DC, Fink G, Fawzi WW, Masanja H, et al. Intimate partner violence, depression, and child growth and development. Pediatrics. 2018 Jul;142(1):1–10.

53. Nguyen PH, Friedman J, Kak M, Menon P, Alderman H. Maternal depressive symptoms are negatively associated with child growth and development: Evidence from rural India. Matern Child Nutr. 2018 Oct;14(4):e12621.

54. Sharmin KN, Sarwar N, Mumu SJ, Taleb DA, Flora MS. Postnatal depression and infant growth in an urban area of Bangladesh. Midwifery. 2019 Jul;74:57–67.

55. Garman EC, Cois A, Tomlinson M, Rotheram-Borus MJ, Lund C. Course of perinatal depressive symptoms among South African women: associations with child outcomes at 18 and 36 months. Soc Psychiatry Psychiatr Epidemiol. 2019 Sep;54(9):1111–23.

56. Mohammedahmed ASA, Koko AEA, Arabi AME, Ibrahim MA. Maternal depression, a hidden predictor for severe acute malnutrition in children aged 6-59 months: a case-control study at Omdurman Paediatrics Teaching Hospital, Sudan. Sudan J Paediatr. 2020;20(2):111–21.

57. Emerson JA, Caulfield LE, Musafiri Kishimata E, Nzanzu JP, Doocy S. Maternal mental health symptoms are positively associated with child dietary diversity and meal frequency but not nutritional status in Eastern Democratic Republic of Congo. Public Health Nutr. 2020 Jul;23(10):1810–9.

58. Susiloretni K, Smith E, Suparmi, Marsum, Agustina R, Shankar A. The psychological distress of parents is associated with reduced linear growth of children: Evidence from a nationwide population survey. PLOS ONE. 2021 Oct 26;16(10).

59. He Q, Cheng G, He S, Tian G, Xie X, Jiang N, et al. Association between maternal postpartum depression and children's physical growth in early childhood: a birth cohort study. Front Pediatr. 2023;11:1135876.

60. Brentani A, Fink G. Maternal depression and child development: Evidence from São Paulo’s Western Region Cohort Study. Rev Assoc Med Bras 1992. 2016 Sep;62(6):524–9.

61. Luchini C, Stubbs B, Solmi M, Veronese N. Assessing the quality of studies in meta-analyses: Advantages and limitations of the Newcastle Ottawa Scale. World J Meta-Anal. 2017 Aug 26;5(4):80–4.

62. Bennett IM, Schott W, Krutikova S, Behrman JR. Maternal mental health, and child growth and development, in four low-income and middle-income countries. J Epidemiol Community Health. 2016;70(2):168–73.

63. Higgins J, Li T, Deeks J, editors. Chapter 6: Choosing effect measures and computing estimates of effect. In: Cochrane Handbook for Systematic Reviews of Interventions version 64 (updated August 2023) [Internet]. Cochrane; 2023. Available from: www.training.cochrane.org/handbook

64. DerSimonian R, Laird N. Meta-analysis in clinical trials. Control Clin Trials. 1986 Sep;7(3):177–88.

65. Knapp G, Hartung J. Improved tests for a random effects meta-regression with a single covariate. Stat Med. 2003 Sep 15;22(17):2693–710.

66. Egger M, Smith GD, Schneider M, Minder C. Bias in meta-analysis detected by a simple, graphical test. BMJ. 1997 Sep 13;315(7109):629–34.

67. Higgins JPT, Thompson SG, Deeks JJ, Altman DG. Measuring inconsistency in meta-analyses. BMJ. 2003 Sep 6;327(7414):557–60.

68. Higgins J, Thomas J, Chadler J, Cumpston M, Page M, Welch V, editors. Cochrane Handbook for Systematic Reviews of Interventions version 6.3 [Internet]. Cochrane; 2022. Available from: www.training.cochrane.org/handbook

69. Stata Statistical Software. College Station, TX: StataCorp LLC; 2021. (StataCorp).

70. Prado EL, Yakes Jimenez E, Vosti S, Stewart R, Stewart CP, Somé J, et al. Path analyses of risk factors for linear growth faltering in four prospective cohorts of young children in Ghana, Malawi and Burkina Faso. BMJ Glob Health. 2019;4(1):e001155.

71. Nasreen HE, Nahar Kabir Z, Forsell Y, Edhborg M. Impact of maternal depressive symptoms and infant temperament on early infant growth and motor development: Results from a population based study in Bangladesh. J Affect Disord. 2013 Apr 5;146(2):254–61.

72. Patel V, DeSouza N, Rodrigues M. Postnatal depression and infant growth and development in low income countries: a cohort study from Goa, India. Arch Dis Child. 2003 Jan;88(1):34–7.

73. Rahman A, Iqbal Z, Bunn J, Lovel H, Harrington R. Impact of maternal depression on infant nutritional status and illness. Arch Gen Psychiatry. 2004 Sep;61(9):946–52.

74. Rahman A, Lovel H, Bunn J, Iqbal Z, Harrington R. Mothers' mental health and infant growth: a case-control study from Rawalpindi, Pakistan. Child Care Health Dev. 2004 Jan;30(1):21–7.

75. Anoop S, Saravanan B, Joseph A, Cherian A, Jacob KS. Maternal depression and low maternal intelligence as risk factors for malnutrition in children: A community based case-control study from South India. Arch Dis Child. 2004;89(4):325–9.

76. Tomlinson M. Parenting and poverty: A complex interaction. In: Tyano S, Keren M, Herrman H, Cox J, editors. Parenthood and mental health: A bridge between infant and adult psychiatry [Internet]. Wiley-Blackwell; 2010. p. 337–48. (World Psychiatric Association evidence and experience in psychiatry). Available from: http://ezp-prod1.hul.harvard.edu/login?url=http://search.ebscohost.com/login.aspx?direct=true&db=psyh&AN=2010-10943-030&site=ehost-live&scope=site

77. Surkan PJ, Kawachi I, Ryan LM, Berkman LF, Vieira LMC, Peterson KE. Maternal depressive symptoms, parenting self-efficacy, and child growth. Am J Public Health. 2008 Jan;98(1):125–32.

78. Adewuya AO, Ola BO, Aloba OO, Mapayi BM, Okeniyi JAO. Impact of postnatal depression on infants' growth in Nigeria. J Affect Disord. 2008 May;108(1–2):191–3.

79. Avan B, Richter LM, Ramchandani PG, Norris SA, Stein A. Maternal postnatal depression and children's growth and behaviour during the early years of life: exploring the interaction between physical and mental health. Arch Dis Child. 2010 Sep;95(9):690–5.

80. Ndokera R, MacArthur C. The relationship between maternal depression and adverse infant health outcomes in Zambia: A cross-sectional feasibility study. Child Care Health Dev. 2011 Jan;37(1):74–81.

81. Kaaya S, Garcia ME, Li N, Lienert J, Twayigize W, Spiegelman D, et al. Association of maternal depression and infant nutritional status among women living with HIV in Tanzania. Matern Child Nutr. 2016 Jul;12(3):603–13.

82. Madeghe BA, Kimani VN, Vander Stoep A, Nicodimos S, Kumar M. Postpartum depression and infant feeding practices in a low income urban settlement in Nairobi-Kenya. BMC Res Notes. 2016 Dec 8;9(1):506.

83. Upadhyay AK, Srivastava S. Effect of pregnancy intention, postnatal depressive symptoms and social support on early childhood stunting: Findings from India. BMC Pregnancy Childbirth [Internet]. 2016;16(1). Available from: https://www.embase.com/search/results?subaction=viewrecord&id=L613897116&from=export

84. Saeed Q, Shah N, Inam S, Shafique K. Maternal depressive symptoms and child nutritional status: A cross-sectional study in socially disadvantaged Pakistani community. J Child Health Care Prof Work Child Hosp Community. 2017;21(3):331–42.

85. Lima TFM, Maciel WM, Alencar MN de, Cruz JA da S, Carvalho CA de, Silva AAM da. Association between maternal depressive symptoms with child malnutrition or child excess weight. Rev Bras Saúde Mater Infant Online. 2017 Jul;17(3):591–601.

86. Wemakor A, Iddrisu H. Maternal depression does not affect complementary feeding indicators or stunting status of young children (6-23 months) in Northern Ghana. BMC Res Notes. 2018;11(408):(25 June 2018).

87. Joshi MN, Raut AV. Maternal depression and its association with responsive feeding and nutritional status of infants: A cross-sectional study from a rural medical college in central India. J Postgrad Med. 2019;65(4):212–8.

88. Ahmadi Gharaei H, Nematollahi S, Moameri H, Madani A, Parsaeian M, Holakouie-Naieni K. Effect of maternal mental health during pregnancy on infant growth at six months of age in Suburban communities in South of Iran. Med J Islam Repub Iran. 2020;34:157.

89. Anato A, Baye K, Tafese Z, Stoecker BJ. Maternal depression is associated with child undernutrition: A cross-sectional study in Ethiopia. Matern Child Nutr. 2020 Jul;16(3):e12934.

90. Ricci H, Nakiranda R, Malan L, Kruger HS, Visser M, Ricci C, et al. Association between maternal postpartum depressive symptoms, socioeconomic factors, and birth outcomes with infant growth in South Africa. Sci Rep. 2023 Apr 7;13(1):5696.

91. Saleh A, Perumal N, Muhihi A, Duggan CP, Ulenga N, Al-Beity FMA, et al. Associations Between Social Support and Symptoms of Antenatal Depression with Infant Growth and Development Among Mothers Living with HIV in Tanzania. AIDS Behav. 2023 May 4;

92. Fekadu Dadi A, Miller ER, Mwanri L. Antenatal depression and its association with adverse birth outcomes in low and middle-income countries: A systematic review and meta-analysis. PloS One. 2020;15(1):e0227323.

93. Gillman MW, Rich-Edwards JW, Huh S, Majzoub JA, Oken E, Taveras EM, et al. Maternal corticotropin-releasing hormone levels during pregnancy and offspring adiposity. Obes Silver Spring Md. 2006 Sep;14(9):1647–53.

94. Slomian J, Honvo G, Emonts P, Reginster JY, Bruyère O. Consequences of maternal postpartum depression: A systematic review of maternal and infant outcomes. Womens Health. 2019 Jan 1;15:1745506519844044.

95. Patton BP, Krans EE, Kim JY, Jarlenski M. The impact of Medicaid expansion on postpartum health care utilization among pregnant women with opioid use disorder. Subst Abuse. 2019 Jul 3;40(3):371–7.

96. Nicklaus S, Boggio V, Chabanet C, Issanchou S. A prospective study of food variety seeking in childhood, adolescence and early adult life. Appetite. 2005 Jun;44(3):289–97.

97. World Health Assembly 65. Global burden of mental disorders and the need for a comprehensive, coordinated response from health and social sectors at the country level: report by the Secretariat [Internet]. World Health Organization; 2012 [cited 2021 Nov 2]. Report No.: A65/10. Available from: https://apps.who.int/iris/handle/10665/78898

98. Rahman A, Iqbal Z, Bunn J, Lovel H, Harrington R. Impact of maternal depression on infant nutritional status and illness: A cohort study. Arch Gen Psychiatry. 2004;61(9):946–52.

99. Lingam R, Gupta P, Zafar S, Hill Z, Yousafzai A, Iyengar S, et al. Understanding care and feeding practices: building blocks for a sustainable intervention in India and Pakistan. Ann N Y Acad Sci. 2014;1308(1):204–17.

100. WHO. Fact sheets - Malnutrition [Internet]. [cited 2021 Nov 2]. Available from: https://www.who.int/news-room/fact-sheets/detail/malnutrition

101. Audelo J, Kogut K, Harley KG, Rosas LG, Stein L, Eskenazi B. Maternal Depression and Childhood Overweight in the CHAMACOS Study of Mexican-American Children. Matern Child Health J. 2016;20(7):1405–14.

102. Wang L, Anderson JL, Dalton WTI, Wu T, Liu X, Zheng S, et al. Maternal depressive symptoms and the risk of overweight in their children. Matern Child Health J. 2013 Jul;17(5):940–8.

103. Constantino B, Silva JC da, Michels M de S, Mastroeni MF. Symptoms of maternal depression and anxiety and their effect on the child's weight status four years after delivery. Early Child Dev Care [Internet]. 2019 Oct 15; Available from: http://ezp-prod1.hul.harvard.edu/login?url=http://search.ebscohost.com/login.aspx?direct=true&db=psyh&AN=2019-61933-001&site=ehost-live&scope=site

104. Sahoo K, Sahoo B, Choudhury AK, Sofi NY, Kumar R, Bhadoria AS. Childhood obesity: causes and consequences. J Fam Med Prim Care. 2015;4(2):187–92.

105. Tzioumis E, Adair LS. Childhood dual burden of under- and over-nutrition in low- and middle-income countries: a critical review. Food Nutr Bull. 2014 Jun;35(2):230–43.

106. Robertson E, Grace S, Wallington T, Stewart DE. Antenatal risk factors for postpartum depression: a synthesis of recent literature. Gen Hosp Psychiatry. 2004 Aug;26(4):289–95.

107. Wisner KL, Sit DKY, McShea MC, Rizzo DM, Zoretich RA, Hughes CL, et al. Onset Timing, Thoughts of Self-harm, and Diagnoses in Postpartum Women With Screen-Positive Depression Findings. JAMA Psychiatry. 2013 May 1;70(5):490–8.

108. Eappen BS, Aguilar M, Ramos K, Contreras C, Prom MC, Scorza P, et al. Preparing to launch the 'Thinking Healthy Programme' perinatal depression intervention in Urban Lima, Peru: experiences from the field. Glob Ment Health. 2018;5:e41–e41.

109. Howard LM, Piot P, Stein A. No health without perinatal mental health. Lancet Br Ed. 2014;384(9956):1723–4.

Supporting information

Supplement 1. The structured search strategy utilized.

S1 Table. PRISMA checklist (Main and abstract).

S2 Table. Summary of the findings on the association between maternal depression and child growth outcome.

S3 Table. Quality assessment of studies with the Newcastle Ottawa Scale.

S1 Fig. Subgroup analysis of the odds ratio between perinatal depression and stunting according to study design.

S1 Fig A. The odds ratio between antepartum depression and stunting according to the study design (n = 4).

S1 Fig B. The odds ratio between postpartum depression and stunting (n = 10).

S2 Fig. Subgroup analysis of the risk ratio between postpartum depression and stunting according to study design (n of sub-studies = 8).

S3 Fig. Subgroup analysis of the odds ratio between postpartum depression and underweight according to study design (n = 9).

S4 Fig. Subgroup analysis of the risk ratio between postpartum depression and underweight according to study design (n of sub-studies = 8).

S5 Fig. Subgroup analysis of the risk ratio between perinatal depression and wasting according to the timepoint of outcome.

S6 Fig. Directed acyclic graph of time-varying characteristics of exposure, mediator-confounders, and outcomes.

Note: APD = antepartum depression, PPD = postpartum depression, L0 = time-varying mediator-confounder (ie, family support) before pregnancy, L1 = time-varying mediator-confounder after delivery

Time-varying exposure: APD and PPD

Time-varying outcome: birth weight and child growth outcome at year 1

Green arrows indicate the causal pathways we want to measure.

Red arrows indicate the backdoor path that induces bias when we measure the association between exposure and outcome.

Figure 1

Click here to access/download;Figure;Fig1_revised.docx

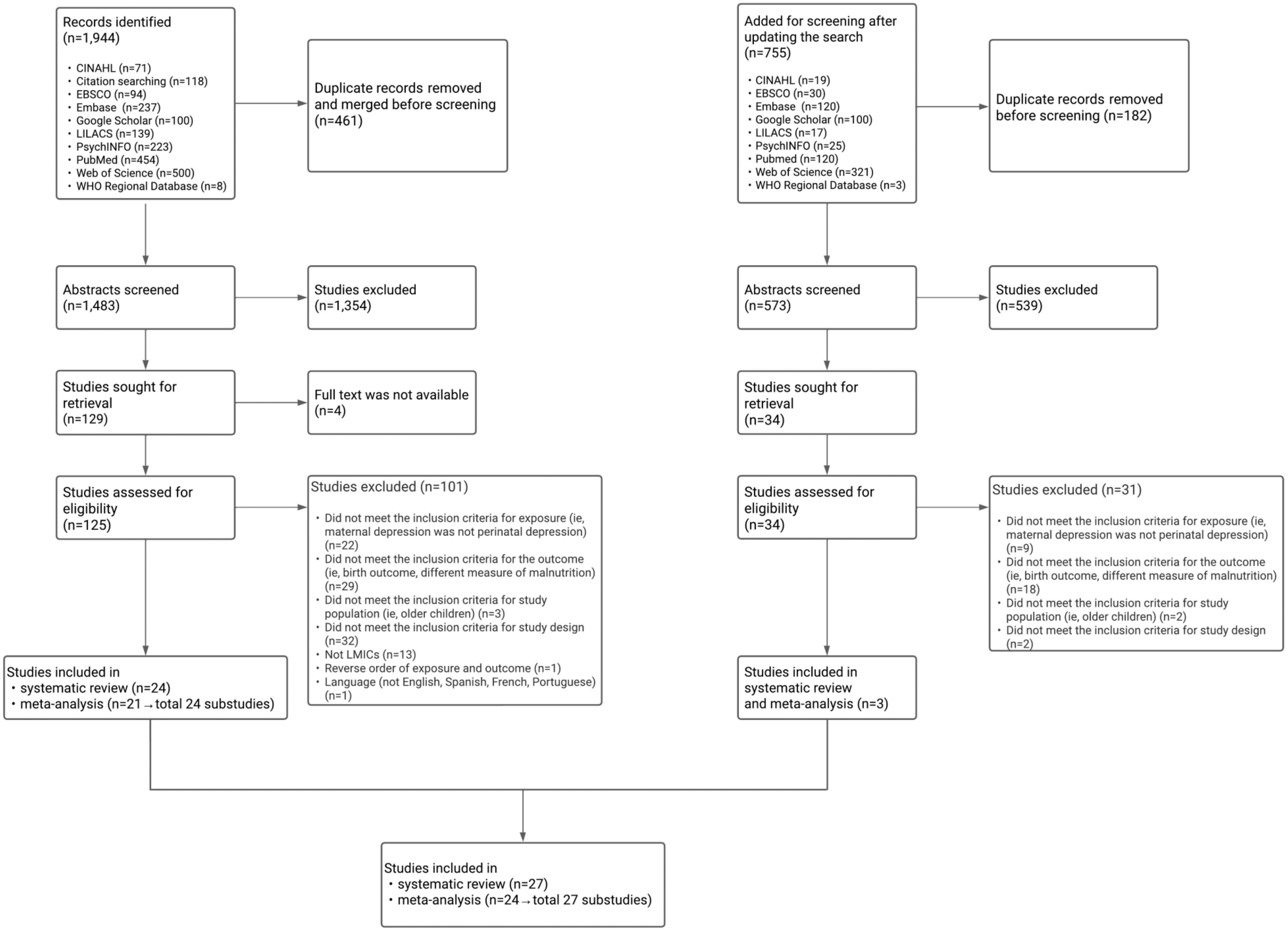

Fig 1. PRISMA flowchart.

From: Page MJ, McKenzie JE, Bossuyt PM, Boutron I, Hoffmann TC, Mulrow CD, et al. The PRISMA 2020 statement: an updated guideline for reporting systematic reviews. BMJ 2021;372:n71. doi: 10.1136/bmj.n71

Note: LMICs = low- and middle-income countries

Click here to access/download;Figure;Fig2_revised.docx

Fig 2. Meta-analysis of odds ratio between perinatal depression and stunting (n = 14) – subgroup analysis according to region and type of perinatal depression (antepartum vs. postpartum).

Fig 2A. Subgroup analysis according to type of perinatal depression.

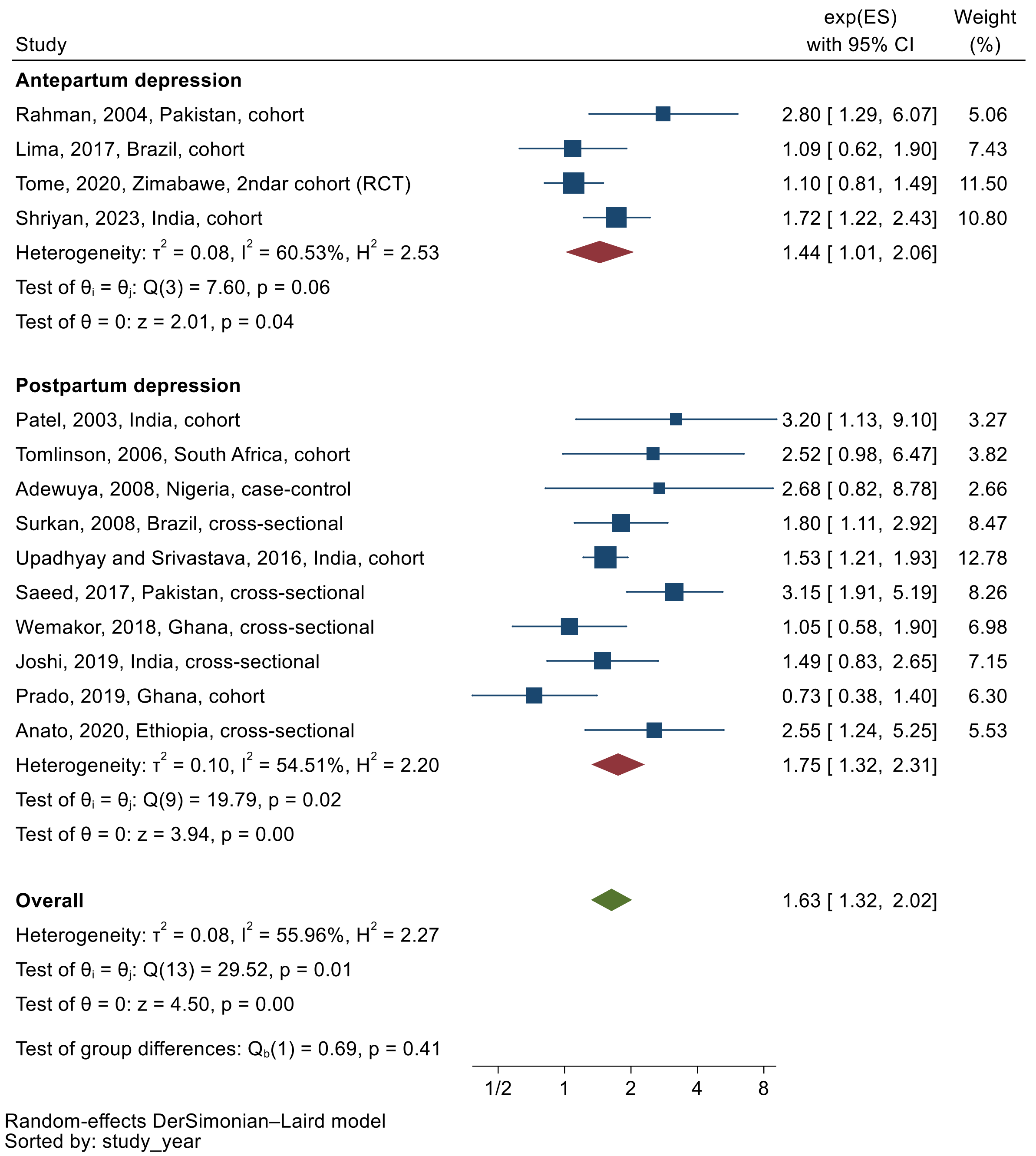

Fig 2B. Subgroup analysis according to region.

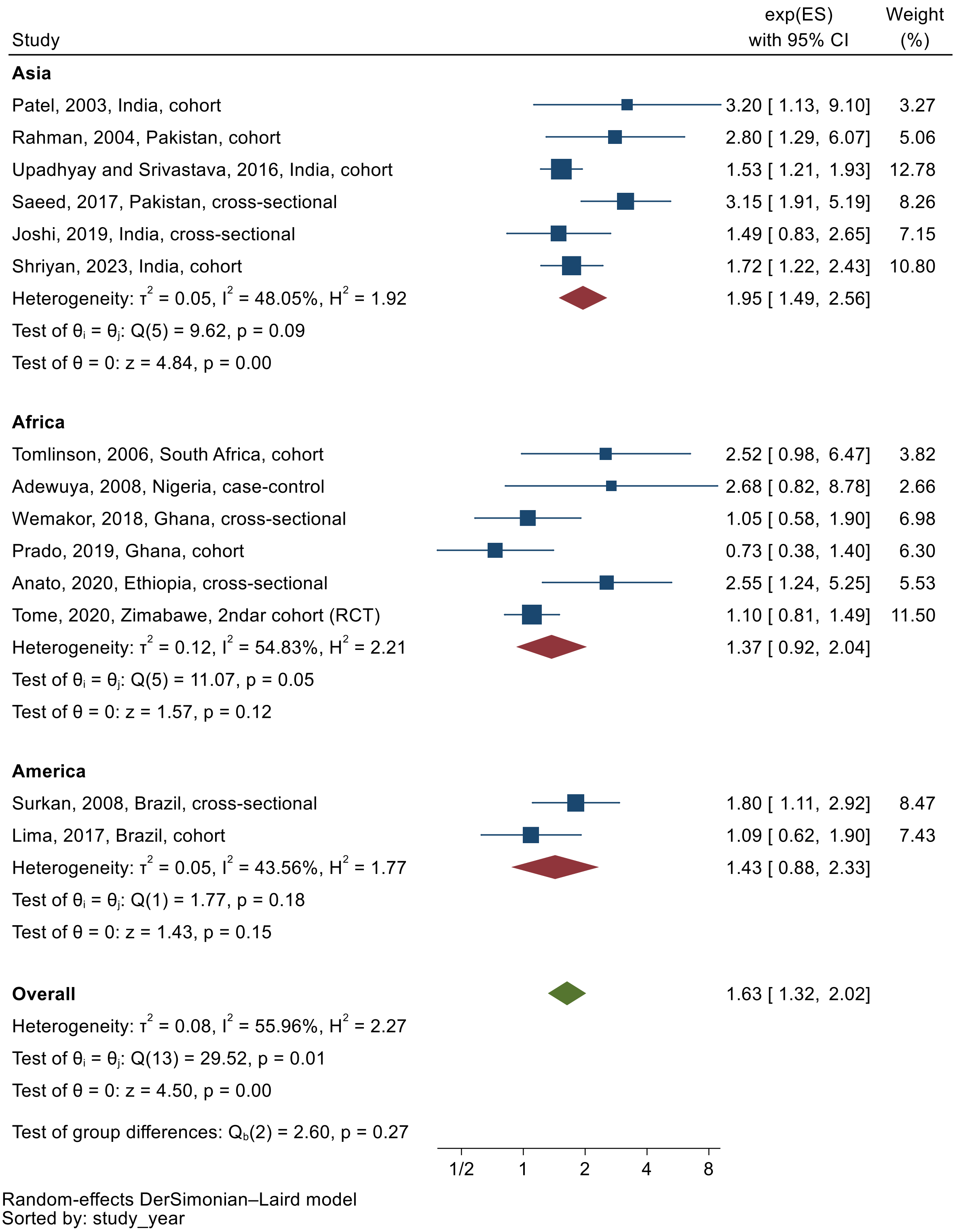

Fig 2 legend.

- The blue square and the horizontal line indicate ORs and their 95% confidence intervals, respectively.
- The red rhombus indicates the overall OR obtained from the DerSimonian and Laird method under a random-effects model.

Click here to access/download;Figure;Fig3_revised.docx

Fig 3. Subgroup analysis of the odds ratio between perinatal depression and stunting according to the time point of stunting measurement.

Fig 3A. The odds ratio between antepartum depression and stunting according to the timepoint of outcome measurement.

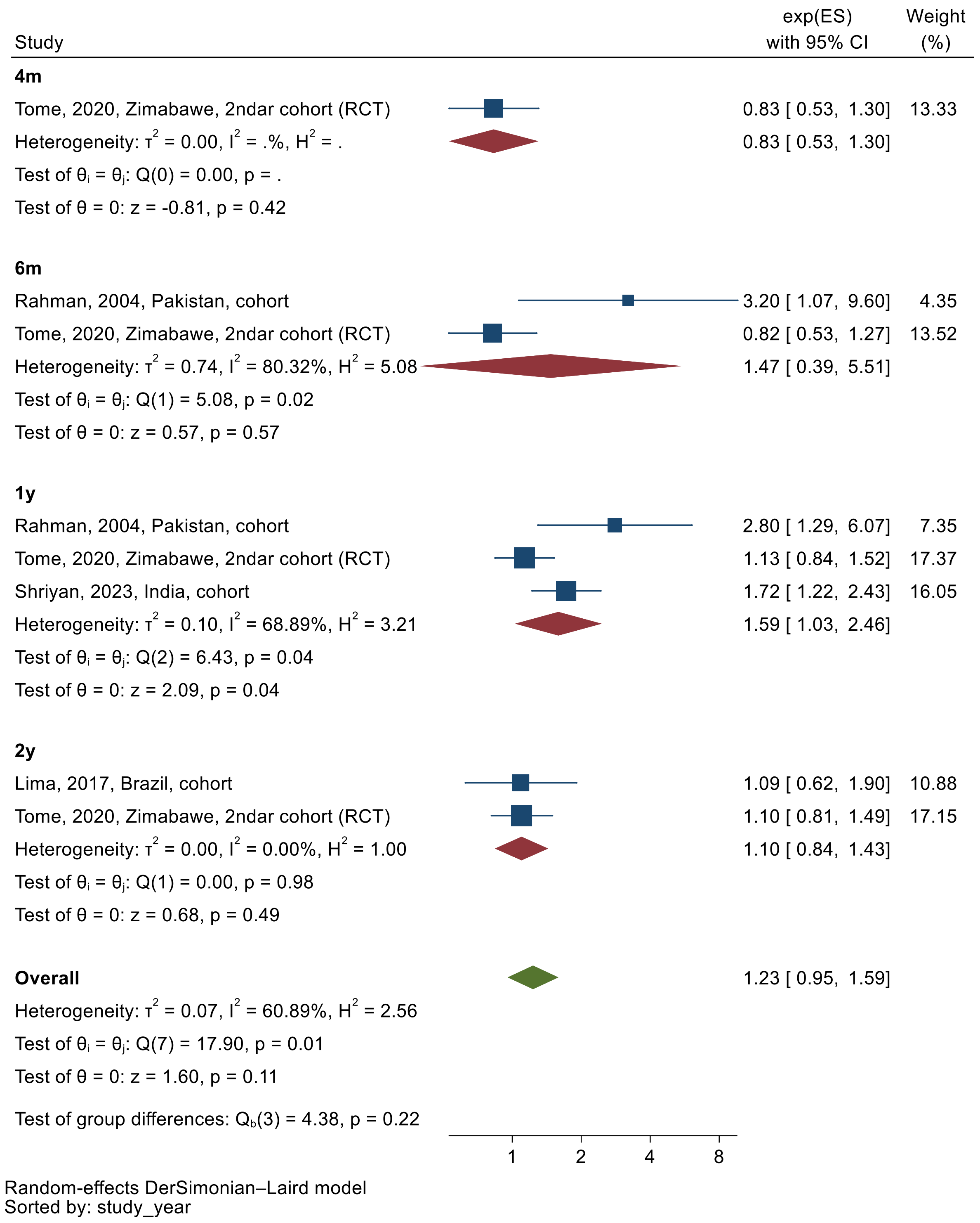

Fig 3B. The odds ratio between postpartum depression and stunting according to the timepoint of outcome measurement.

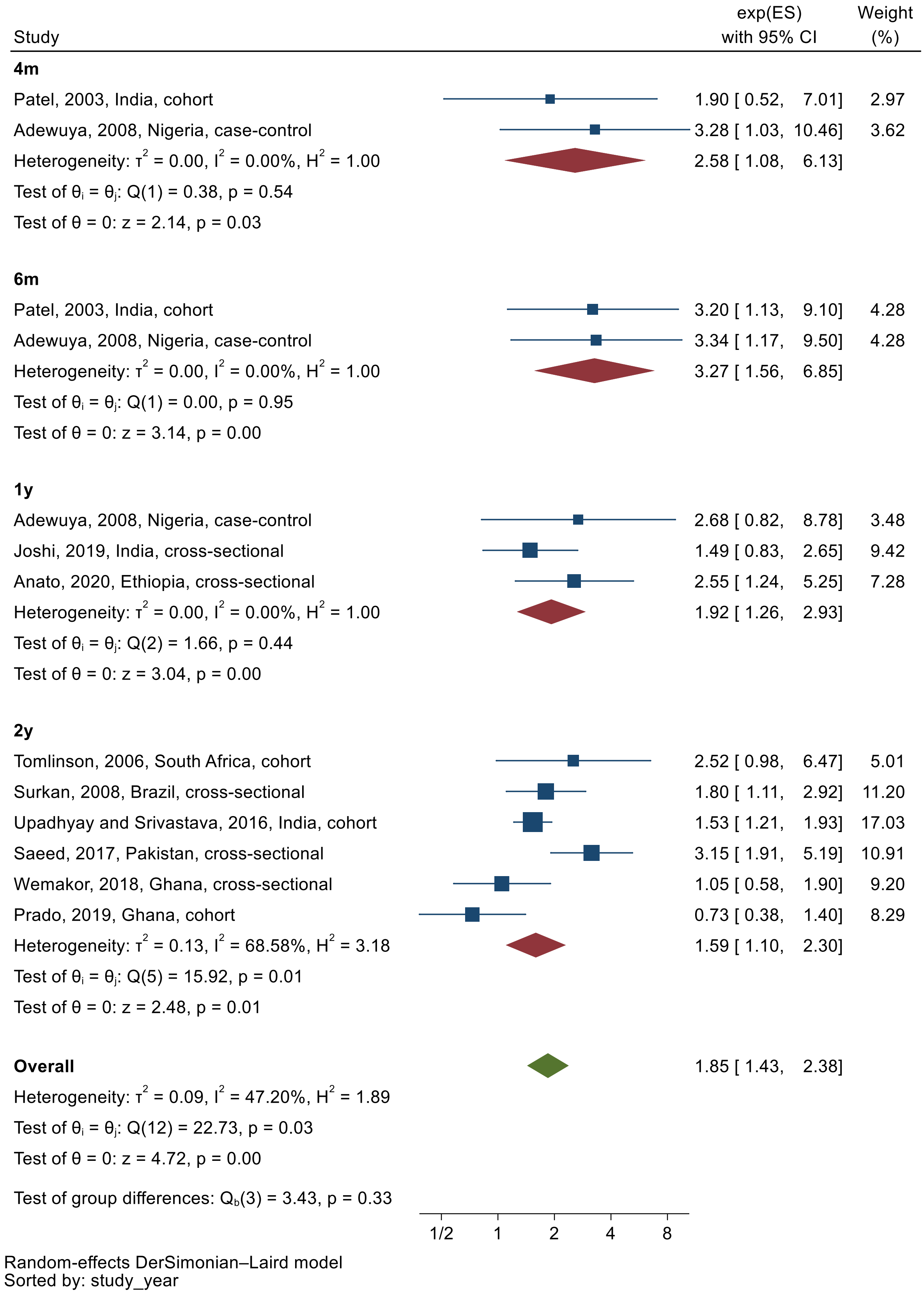

Fig 3 legend.

- The blue square and the horizontal line indicate ORs and their 95% confidence intervals, respectively.
- The red rhombus indicates the overall OR obtained from the DerSimonian and Laird method under a random-effects model.
- The overall effect in the bottom of these figures should be ignored, given the repeated estimates of the same study.

Figure 4
Click here to access/download;Figure;Fig4_revised.docx

Fig 4. Meta-analysis of the risk ratio between perinatal depression and stunting of children.

Fig 4A. Subgroup analysis according to type of perinatal depression.[a]

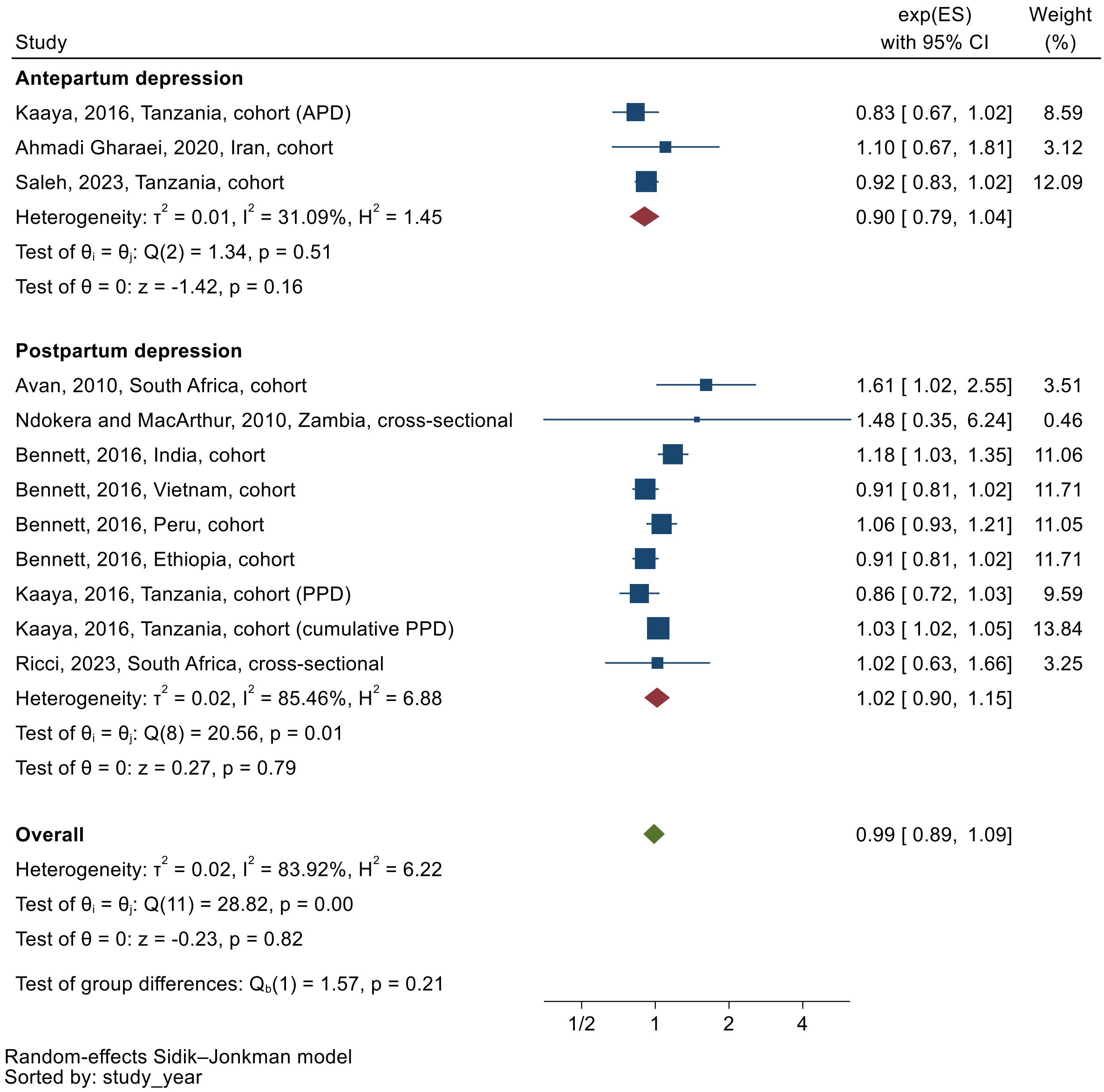

Fig 4B. Subgroup analysis according to region.

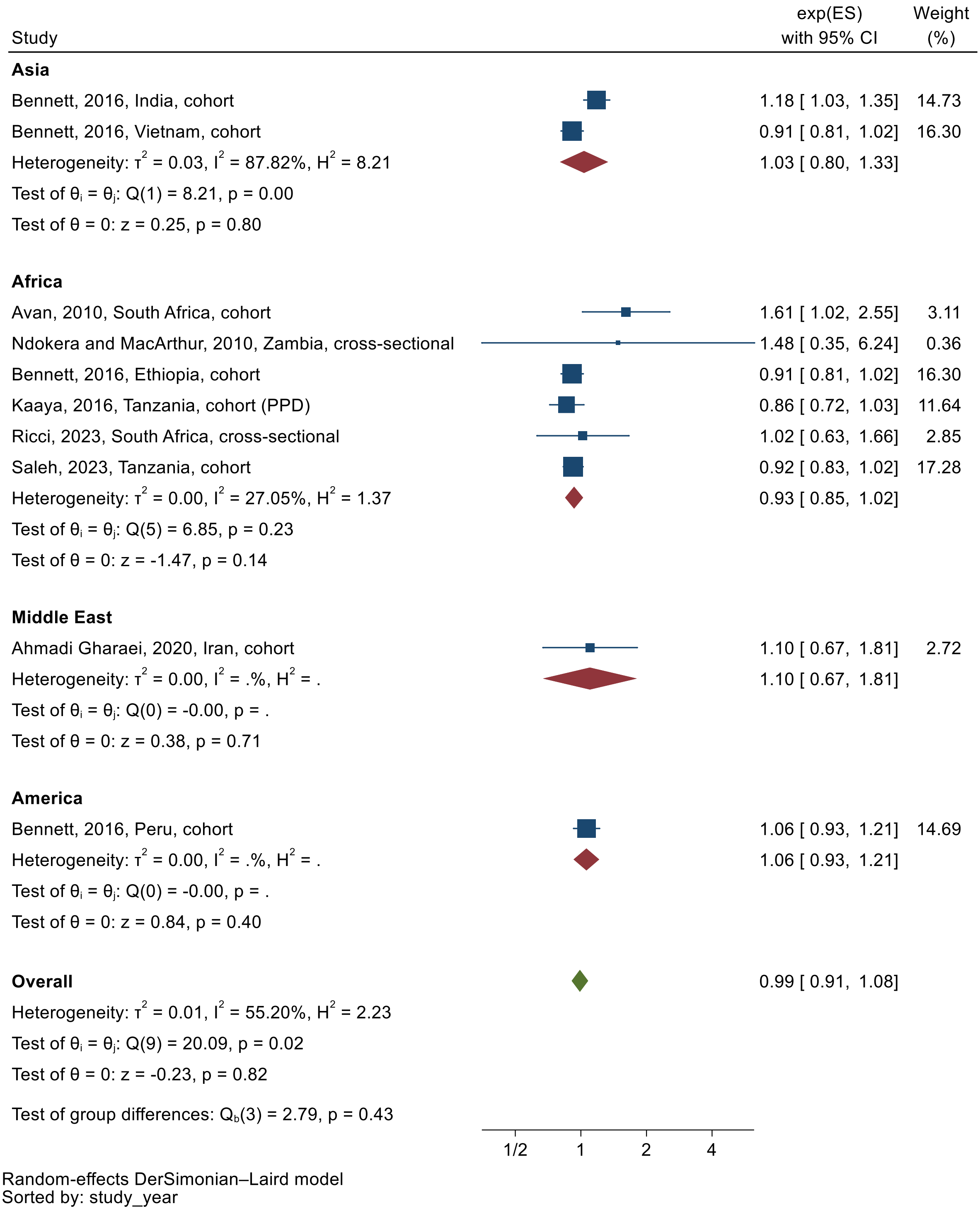

Fig 4 legend.

a: Bennett et al. (2016) was comprised of 4 different sub-studies, and Kaaya et al. (2016) reported the association with APD and PPD, separately.

- The blue square and the horizontal line indicate RRs and their 95% confidence intervals, respectively.
- The red rhombus indicates the overall RR obtained from the DerSimonian and Laird method under a random-effects model.
- The overall effect in the bottom of these figures should be ignored given the repeated estimates of the same study and population (Kaaya et al. (2016)).

Figure 5 Click here to access/download;Figure;Fig5_revised.docx

Fig 5. Subgroup analysis of the risk ratio between perinatal depression and stunting according to time point of stunting measurement.

Fig 5A. The risk ratio between antepartum depression and stunting according to the timepoint of outcome measurement.

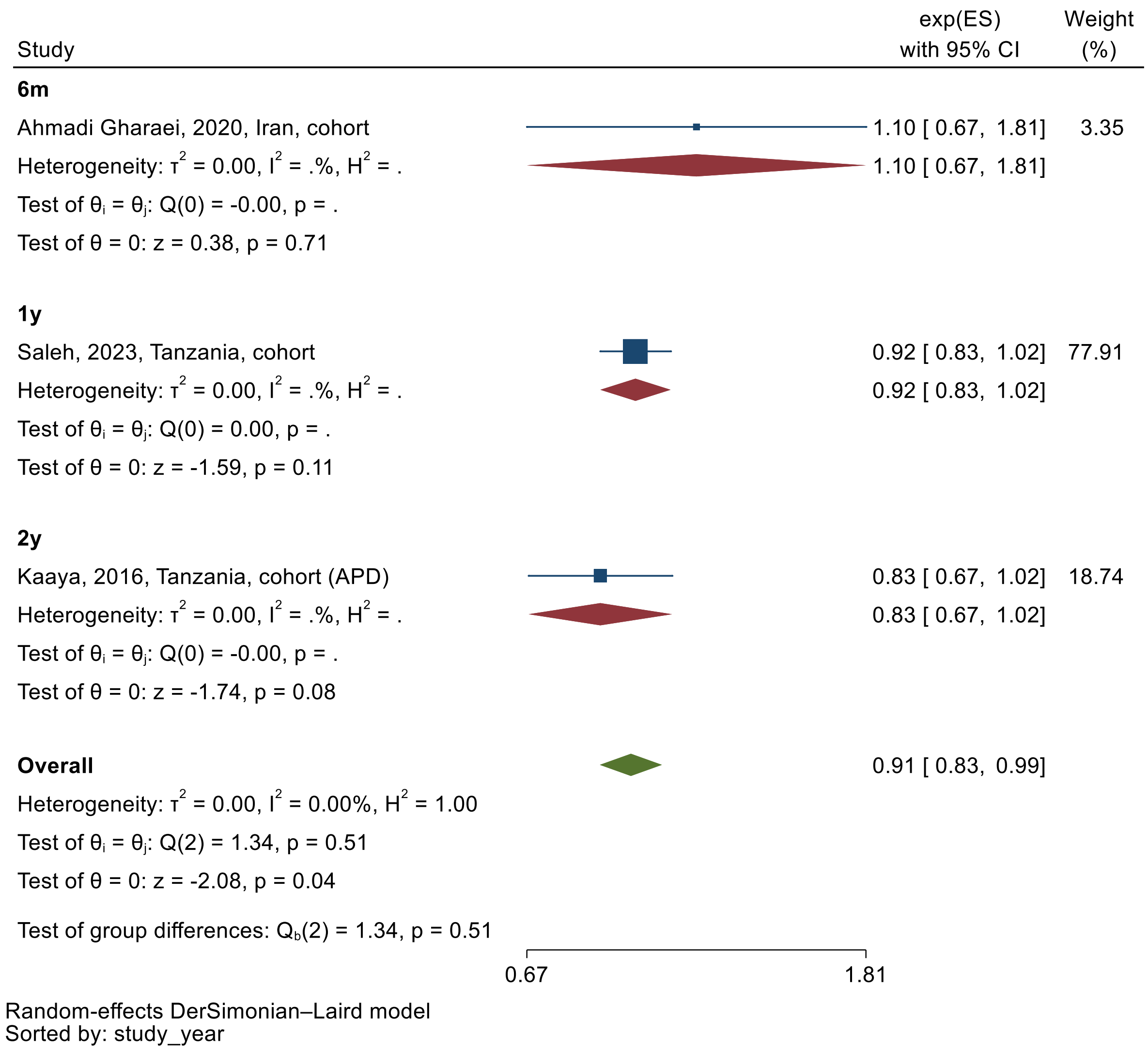

Fig 5B. The risk ratio between postpartum depression and stunting according to timepoint of outcome measurement.

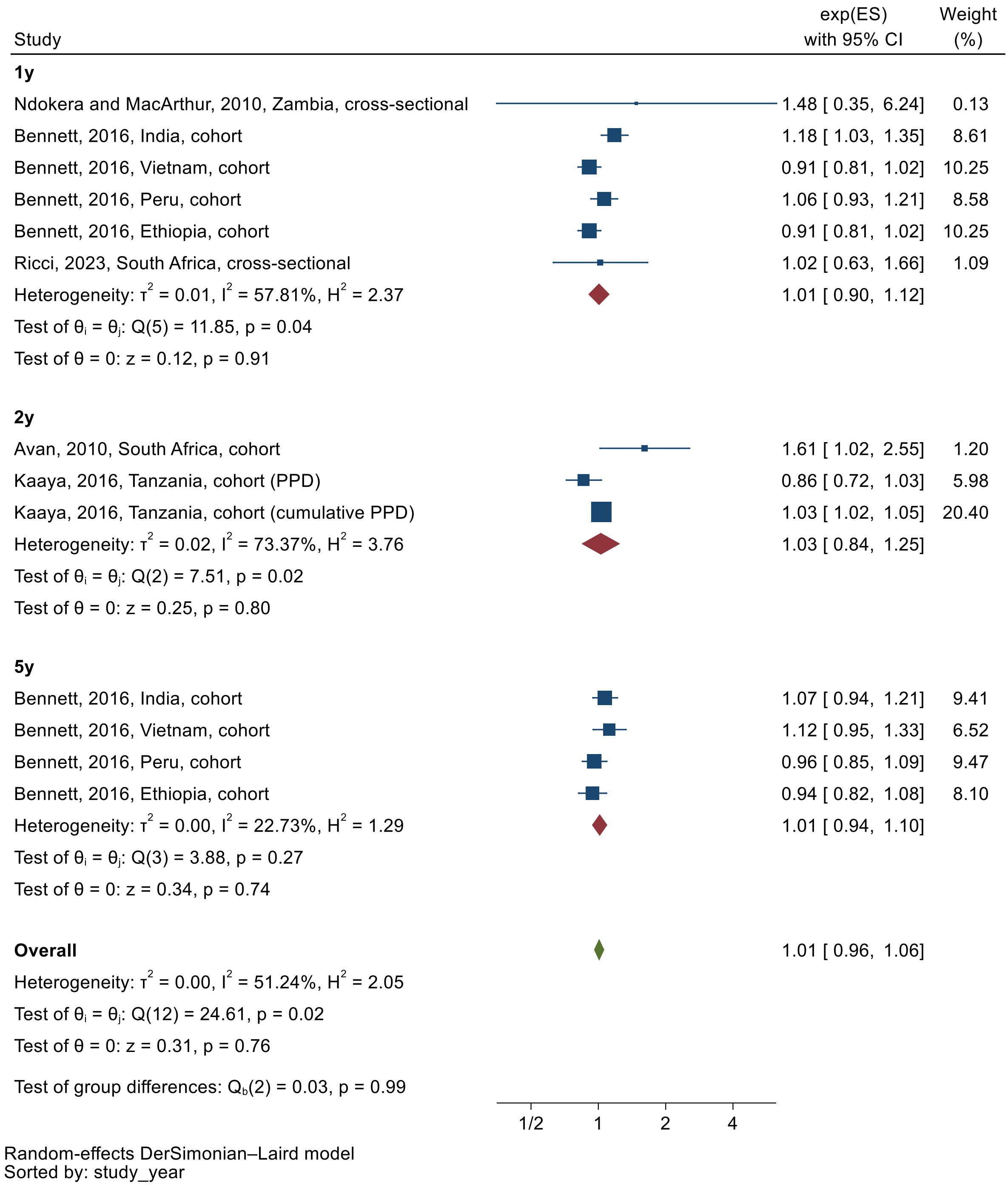

Fig 5 legend.

- The blue square and the horizontal line indicate RRs and their 95% confidence intervals, respectively.
- The red rhombus indicates the overall RR obtained from the DerSimonian and Laird method under a random-effects model.
- The overall effect in the bottom of these figures should be ignored given the repeated estimates of the same study and population (Kaaya et al. (2016)).

Fig 6. Meta-analysis of odds ratio between perinatal depression and underweight (n = 10) – subgroup analysis according to region and type of perinatal depression (antepartum vs. postpartum).

Fig 6A. Subgroup analysis according to type of perinatal depression.

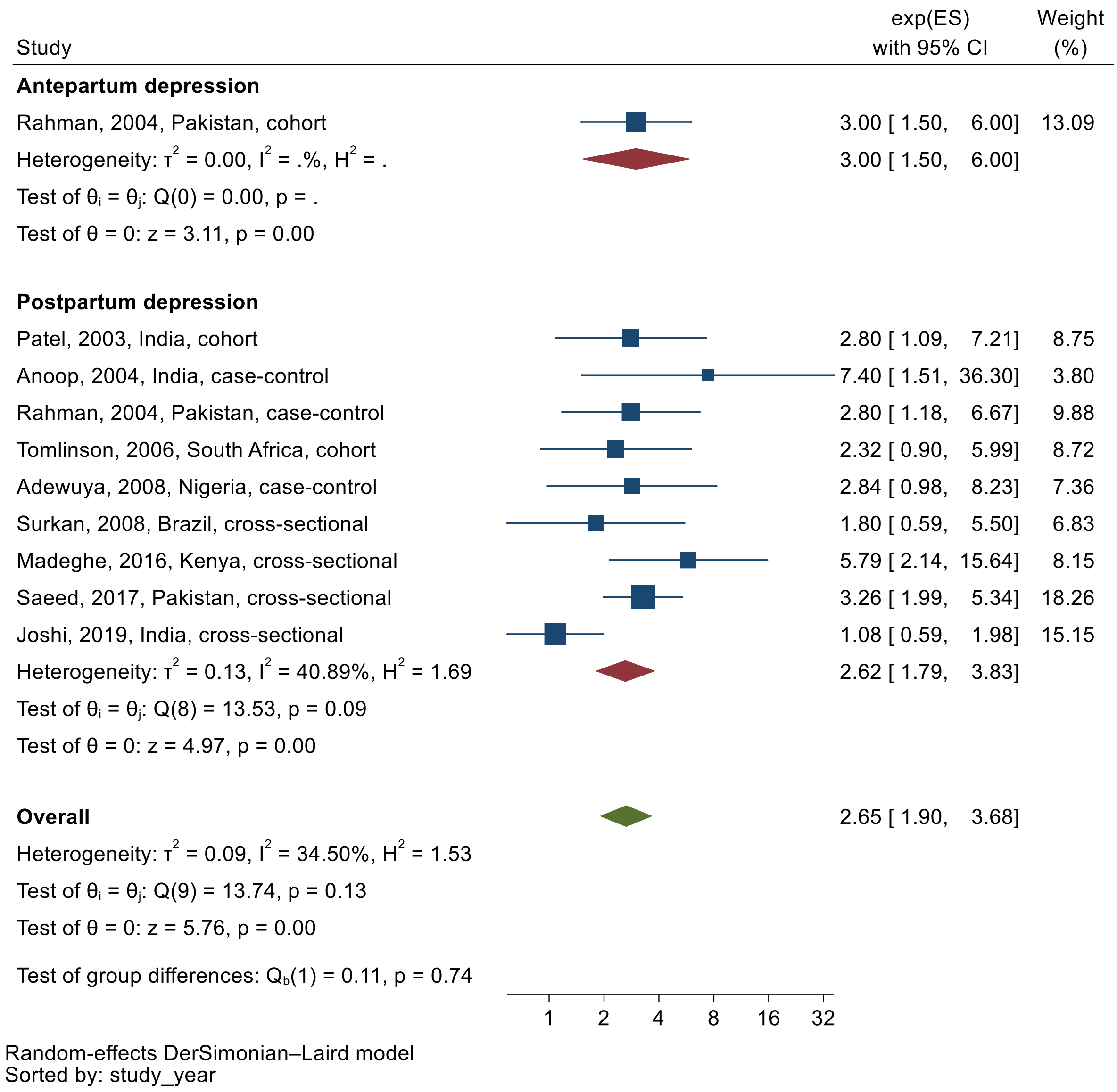

Fig 6B. Subgroup analysis according to region.

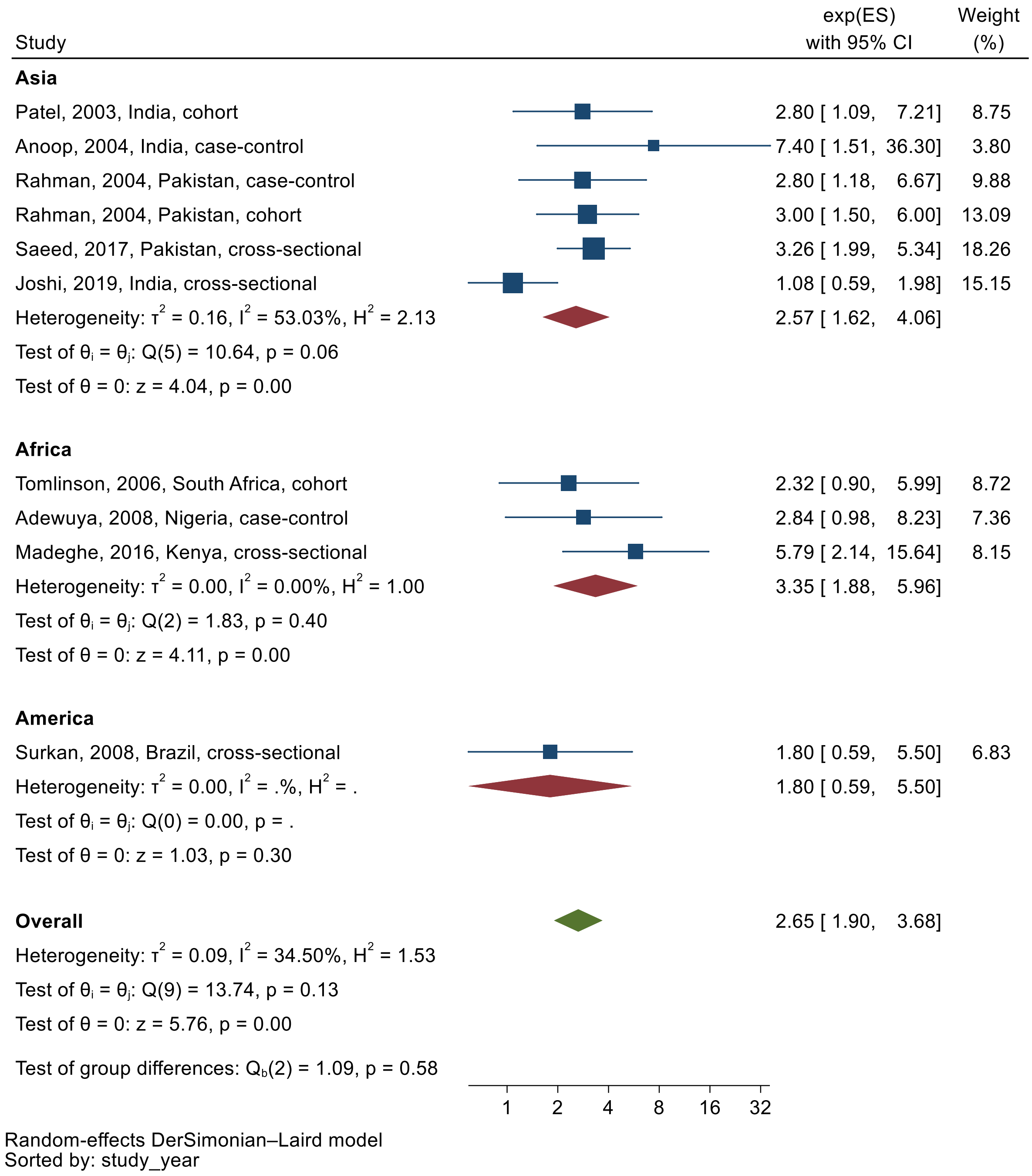

Fig 6 legend.

- The blue square and the horizontal line indicate ORs and their 95% confidence intervals, respectively.
- The red rhombus indicates the overall OR obtained from the DerSimonian and Laird method under a random-effects model.

Figure 7

Click here to access/download;Figure;Fig7_revised.docx

Figure 7. Meta-analysis of the odds ratio between postpartum depression and underweight – subgroup analysis according to time point of underweight measurement (n = 9).

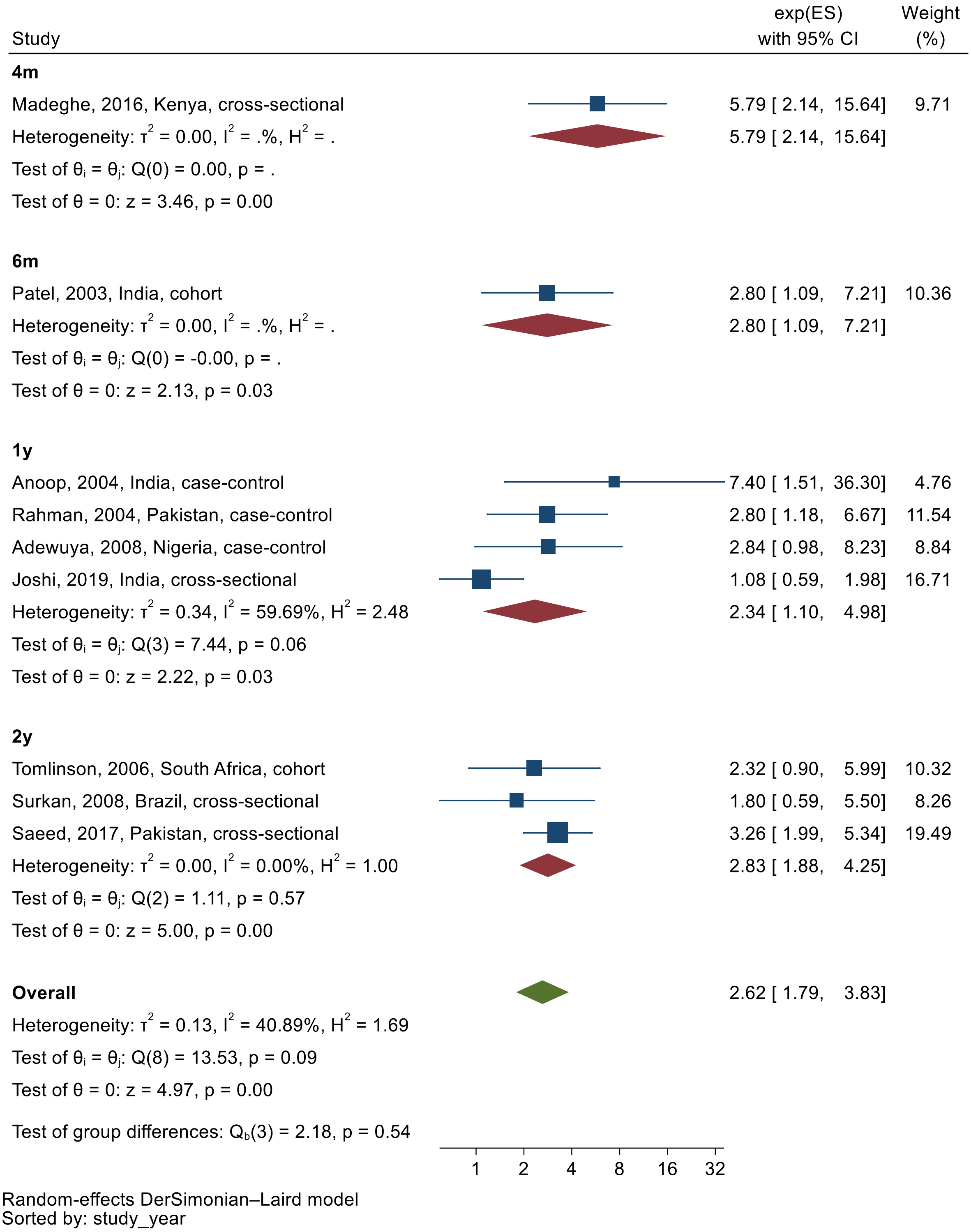

Figure 7 legend.

- The blue square and the horizontal line indicate ORs and their 95% confidence intervals, respectively.
- The red rhombus indicates the overall OR obtained from the DerSimonian and Laird method under a random-effects model.

Click here to access/download;Figure;Fig8_revised.docx

Fig 8. Meta-analysis of the risk ratio between perinatal depression and underweight of children.

Fig 8A. Subgroup analysis according to type of perinatal depression.[a]

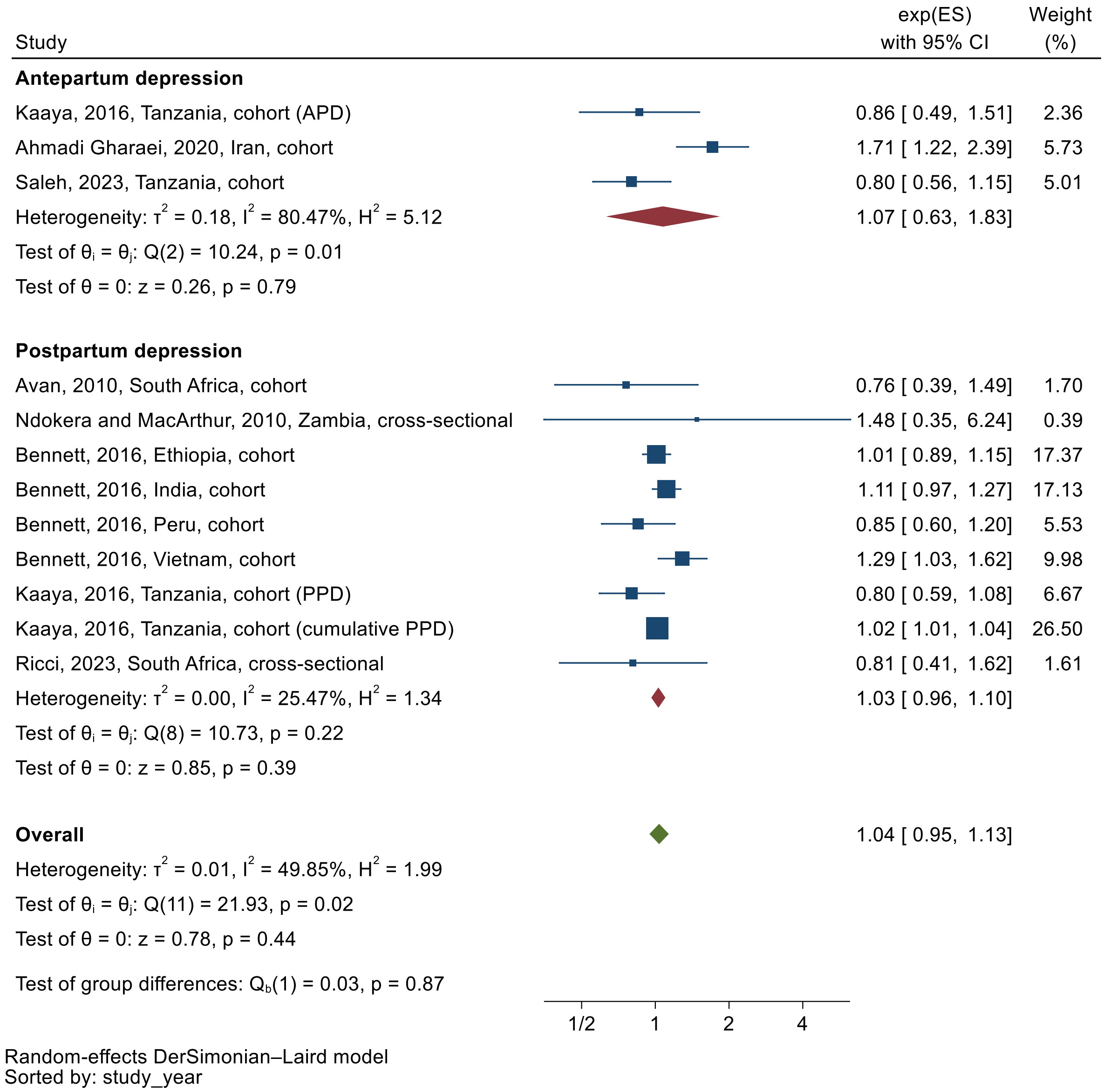

Fig 8B. Subgroup analysis according to region.

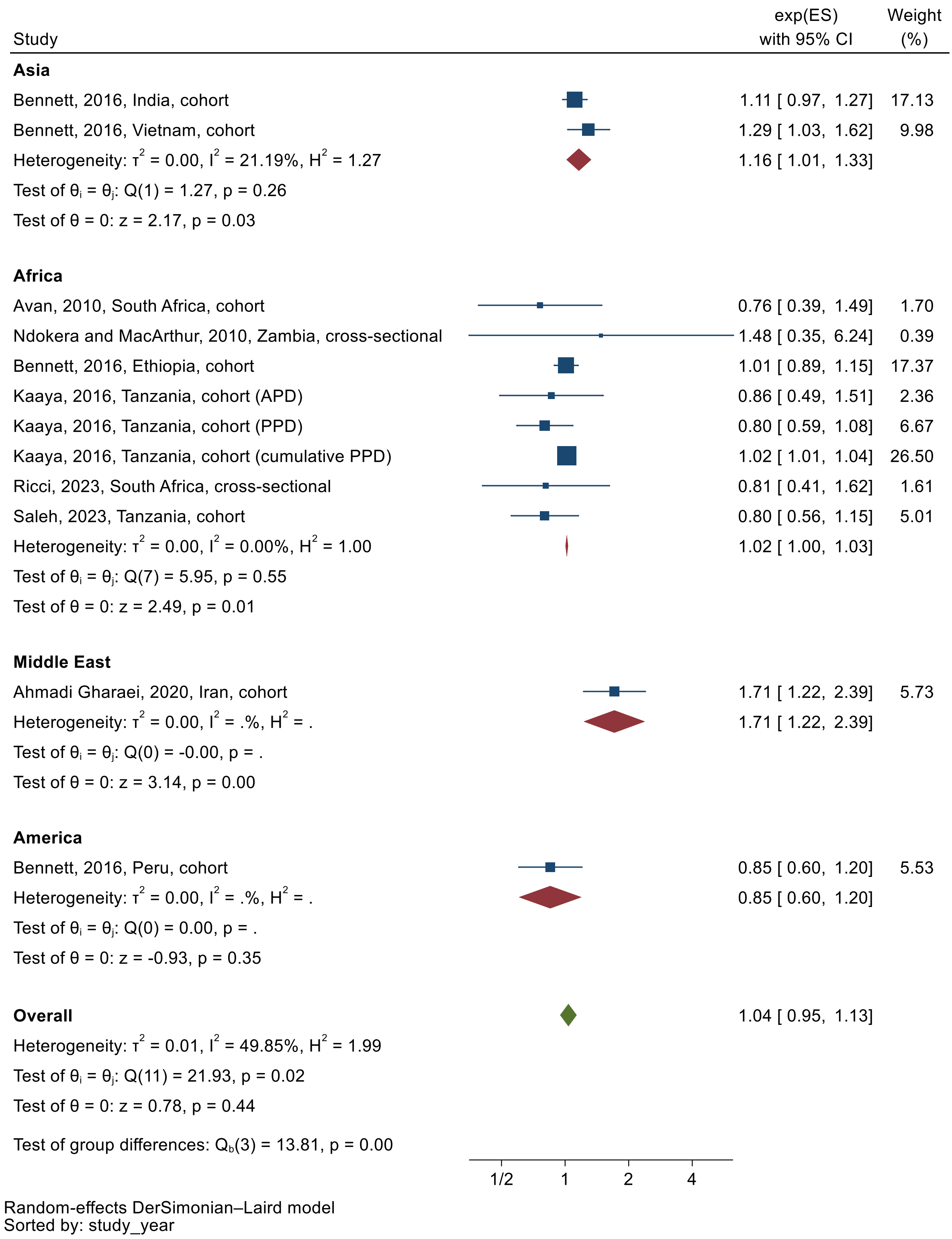

Fig 8 legend.

a: Bennett et al. (2016) was comprised of 4 different sub-studies, and Kaaya et al. (2016) reported the association with APD, PPD, and cumulative PPD separately.

- The blue square and the horizontal line indicate RRs and their 95% confidence intervals, respectively.
- The red rhombus indicates the overall RR obtained from the DerSimonian and Laird method under a random-effects model.
- The overall effect in the bottom of these figures should be ignored, given the repeated estimates of the same study and population (Kaaya et al. (2016)).

Figure 9 Click here to access/download;Figure;Fig9_revised.docx

Fig 9. Subgroup analysis of the risk ratio between perinatal depression and underweight according to time point of stunting measurement.

Fig 9A. The risk ratio between antepartum depression and underweight according to the timepoint of outcome measurement.

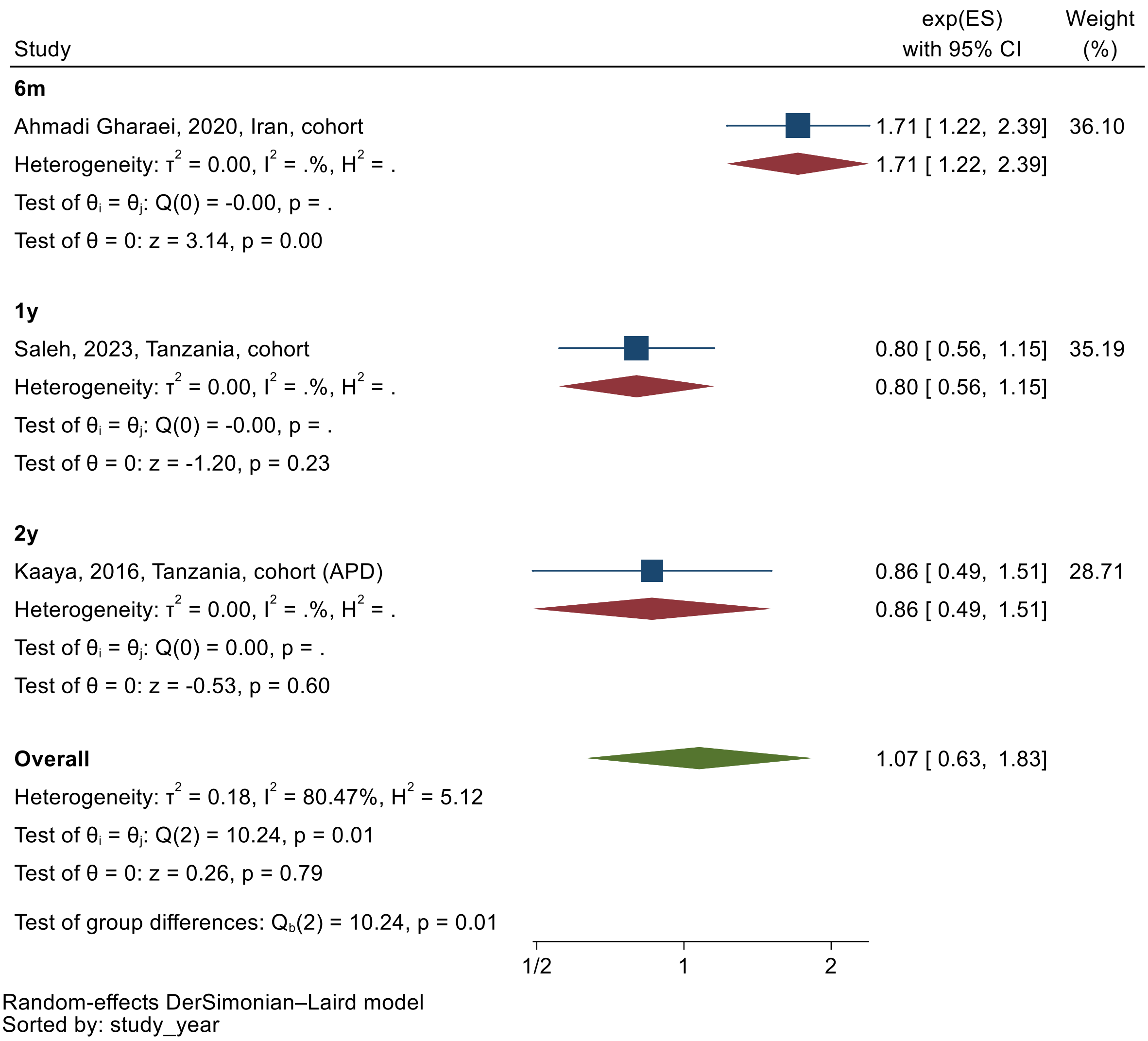

Figure 9B. The risk ratio between postpartum depression and underweight according to timepoint of outcome measurement.

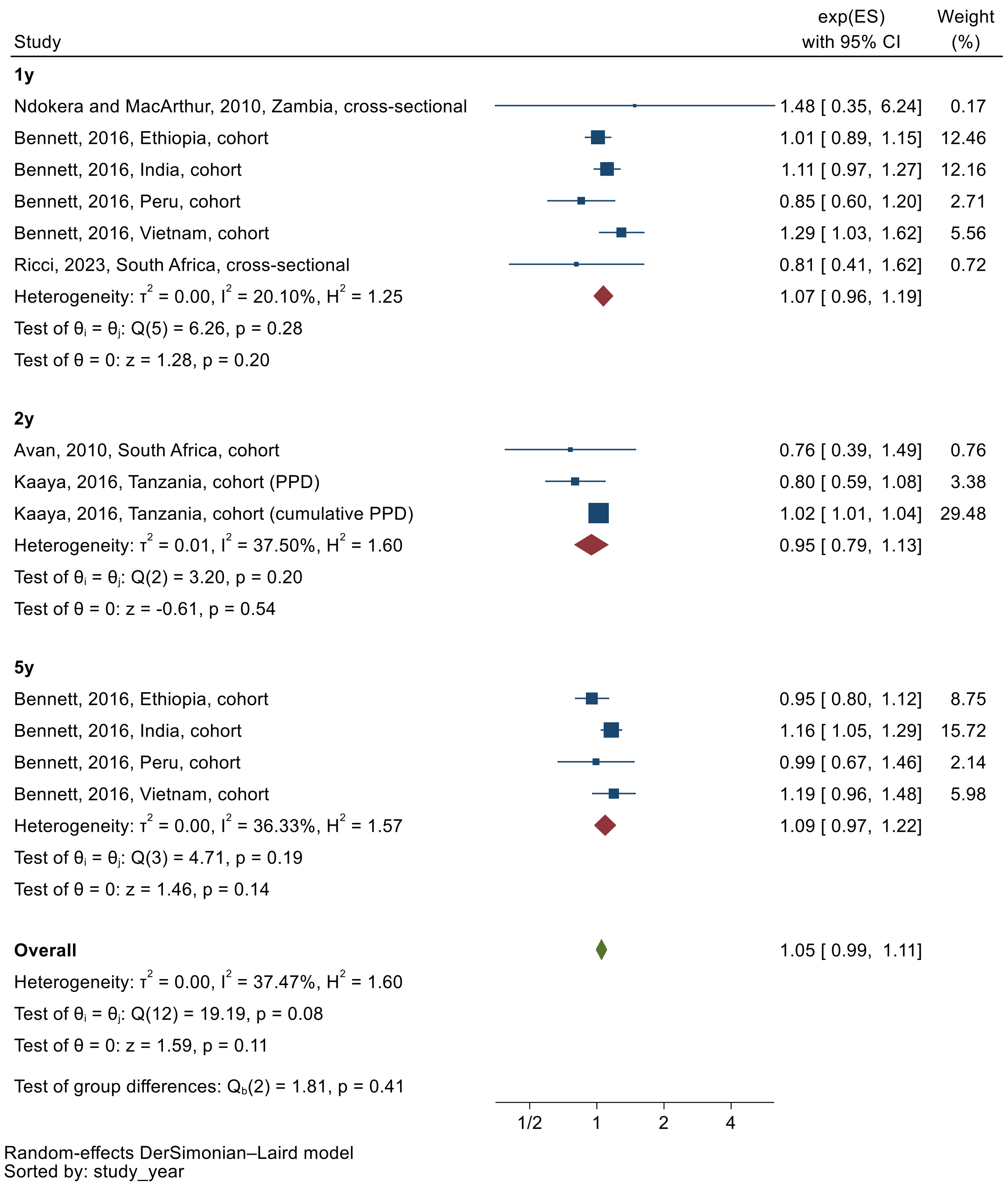

Fig 9 legend.

- The blue square and the horizontal line indicate ORs and their 95% confidence intervals, respectively.
- The red rhombus indicates the overall OR obtained from the DerSimonian and Laird method under a random-effects model.
- The overall effect in the bottom of these figures should be ignored given the repeated estimates of the same study.

Fig 10. Subgroup analysis of the risk ratio between perinatal depression and wasting according to type of perinatal depression.[a]

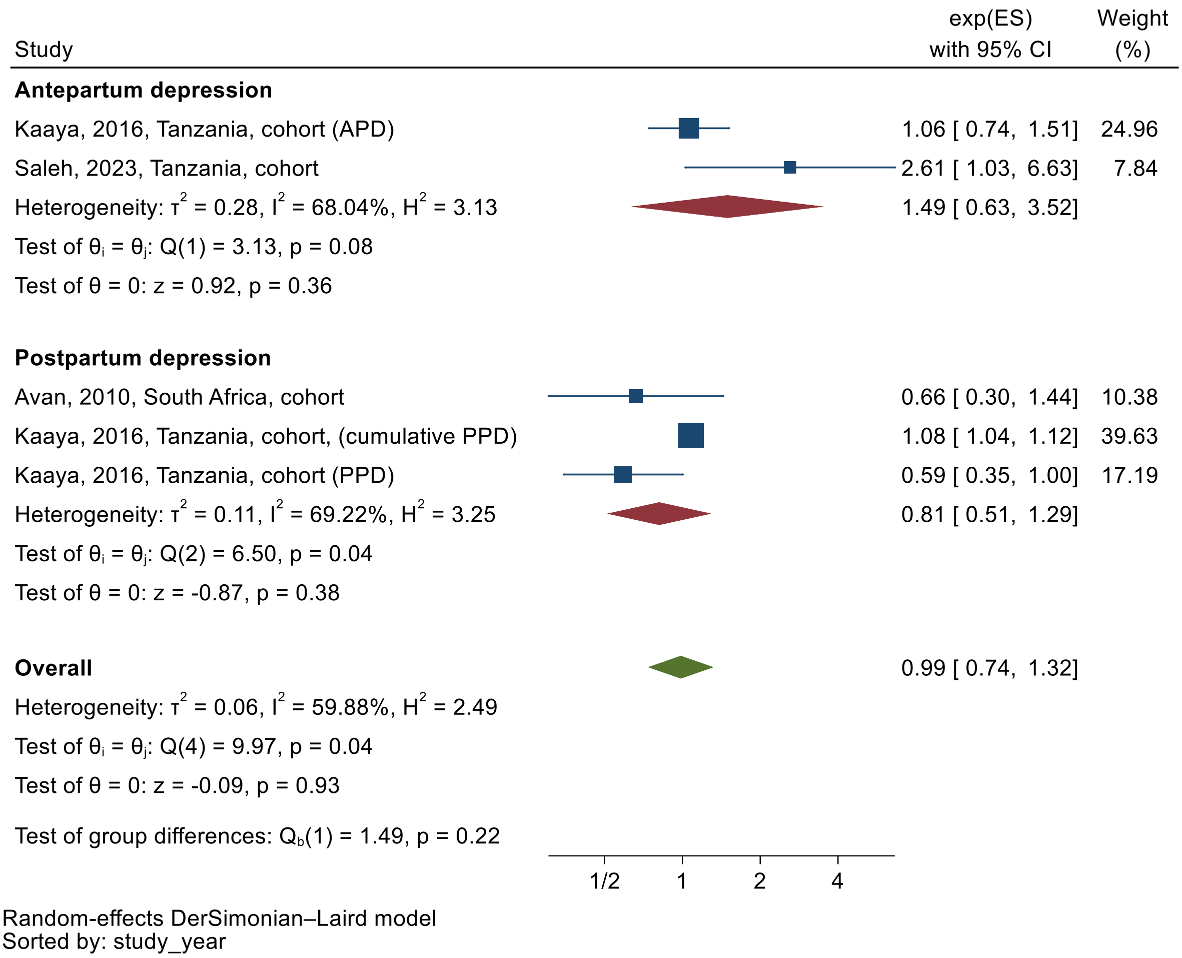

Fig 10 legend.

- The blue square and the horizontal line indicate RRs and their 95% confidence intervals, respectively.
- The red rhombus indicates the overall RR obtained from the DerSimonian and Laird method under a random-effects model.

 Click here to access/download;Figure;Fig11_revised.docx

Fig 11. Contour-enhanced funnel plots of included studies in the meta-analysis.

A. A funnel plot of studies reporting odds ratios between perinatal depression and stunting.

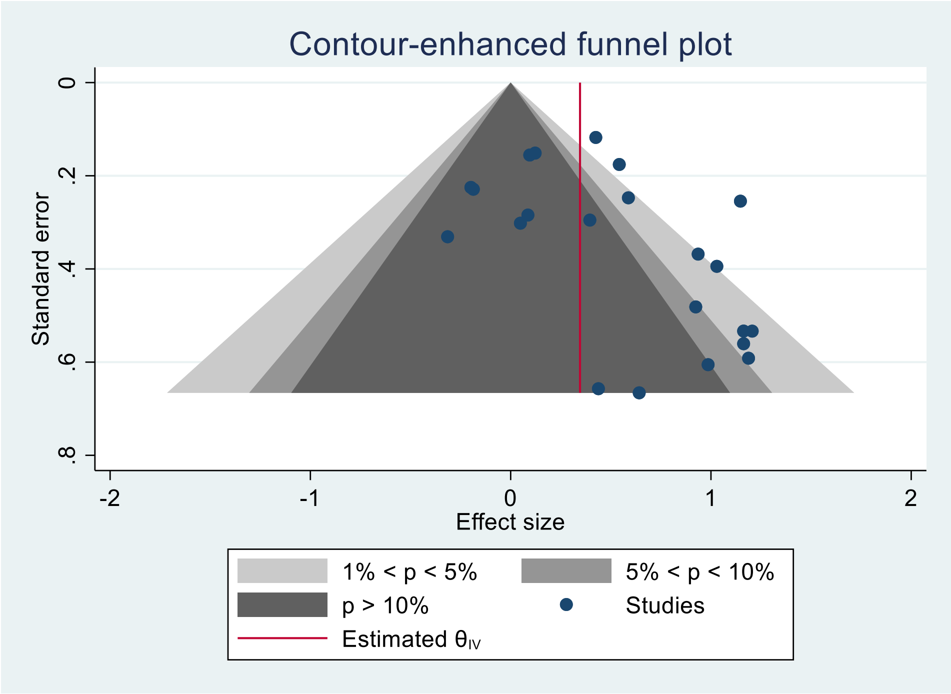

B. A funnel plot of studies reporting odds ratios between perinatal depression and underweight.

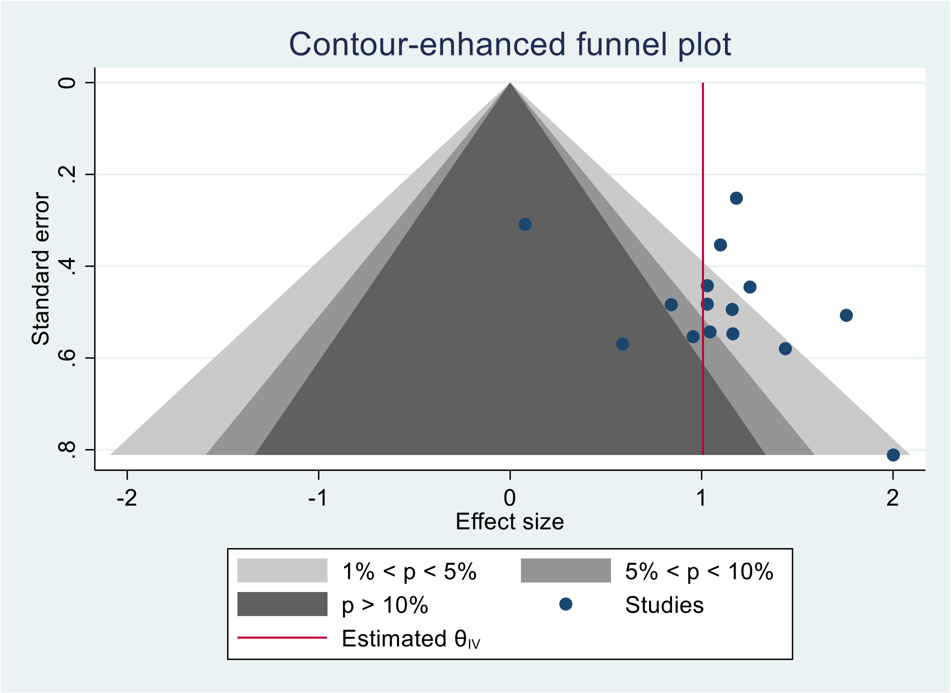

C. A funnel plot of studies reporting risk ratios between perinatal depression and stunting.

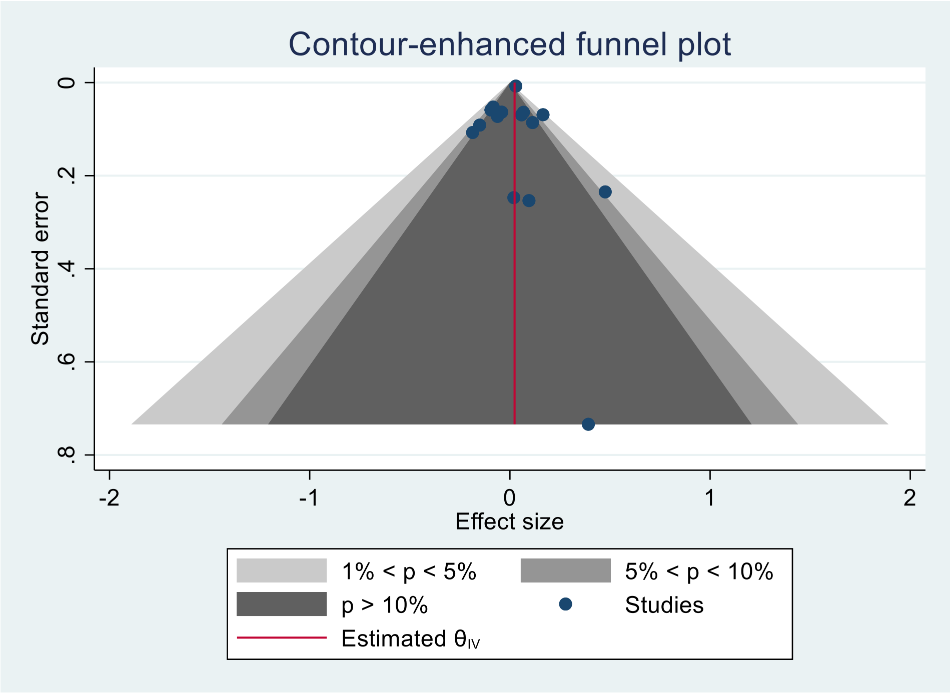

D. A funnel plot of studies reporting risk ratios between perinatal depression and underweight.

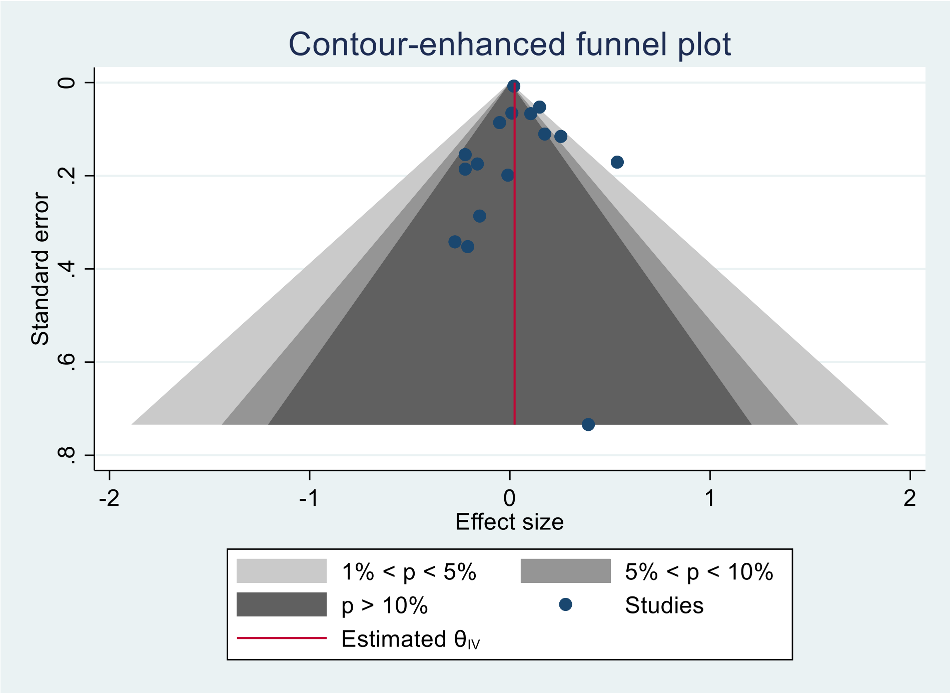

Click here to access/download
Supporting Information
Supplementary_Material_2023_revision.docx

S1 Table. PRISMA Checklist

Click here to access/download
Supporting Information
S1 Table_PRISMA Checklist.docx

University of California
San Francisco

Hyelee Kim, MD, MAS, MS

Ph.D. student of Epidemiology and Translational Science Program

Department of Epidemiology & Biostatistics

University of California San Francisco

550 16th Street, Second Floor

San Francisco, CA 94158

Hyelee.Kim@ucsf.edu

January 18, 2024

Dear *PLOS Global Public Health* editors,

I am writing to summarize the changes in the resubmitted version of our manuscript (PGPH-S-23-02443), titled "Perinatal depression and adverse child growth outcomes in low- and middle-income countries (LMICs): a systematic review and meta-analysis," compared to the submitted version in June 2023 (PGPH-D-23-01052).

Firstly, as demonstrated in updated Fig1 (PDF page 55 out of 83), we updated the previous literature search up until September 2023 using the same search words, databases, and the systematic review tool (Covidence) we used in the previous version. The search words are presented in the supplementary materials (Supplement 1).

Secondly, in the previous version, among the articles we found relevant to our research questions, we needed to trim down the list to obtain the estimate from more homogeneous characteristics (final list: a total of 20 sub-studies from 17 papers). In this version, in addition to 3 papers we identified in the updated search, we included 27 papers in the systematic review and a total of 27 sub-studies from 24 papers in the meta-analysis among the studies identified in the previous version (Fig 1). Instead, we conducted various additional subgroup analyses, including time-point of outcome measurement (i.e., Fig 3A and 3B), study designs (i.e., S1 Fig A and B), and conducting sensitivity analysis.

Thirdly, in the additional subgroup analyses according to study region (i.e., Fig 4B), we found that the study region might be a meaningful factor that affects the heterogeneity of estimates. Therefore, we reconducted the meta-analyses, including the updated study list and added the estimates of the meta-regression in Table 4.

Fourthly, while our study's outcome was child adverse growth outcomes, many studies sought the association between perinatal depression and child growth outcomes. Given the indirect relevance of the findings of those studies, we also added the summary of the studies from those studies in the S2 Table.

University of California San Francisco

Hyelee Kim, MD, MAS, MS

Ph.D. student of Epidemiology and Translational Science Program

Department of Epidemiology & Biostatistics

University of California San Francisco

550 16th Street, Second Floor

San Francisco, CA 94158

Hyelee.Kim@ucsf.edu

Lastly, we added S6 Fig, a directed acyclic graph of time-varying characteristics of exposure, mediator-confounders, and outcomes, which explains why adjusting birthweight could be problematic for answering the association between perinatal depression and child adverse outcomes.

Thank you so much again for considering our paper for your journal. For further questions, please contact Dr. Hyelee Kim at Hyelee.Kim@ucsf.edu.

Sincerely,

Hyelee Kim MD, MAS, MS
