# Supplementary material for: Perinatal depression and adverse child growth outcomes in low-income and middle-income countries (LMICs): A systematic review and meta-analysis
Source: PLOS Glob Public Health. 2024 Oct 28;4(10):e0003586. doi: 10.1371/journal.pgph.0003586 (PMC11516009; doi:10.1371/journal.pgph.0003586)
Supplement: Attachment — Submitted filename: PlosOne Public Health Editor.pdf [file pgph.0003586.s020.pdf]

30th August, 2024.

The Editor

PLOS Global Public Health

Dear Sir,

LETTER OF ACCEPTANCE FOR MANUSCRIPT NO.: PGPH-D-23-02443.

Sorry for getting back to you rather late on my final review of the paper with the manuscript number: PGPH-D-23-02443. Based on my thorough review of the revised submitted manuscript, I have come to the following conclusion and decision.

My general comments are that; **1.** the paper is a good piece, **2.** the analyses carried out by the authors are rigorous and merit publication for the knowledge to share with the academic and health community and **3.** the authors have addressed over 95% of my previous comments made in the earlier draft. While I recommend the paper for onward publication, I suggest that the authors work on redesigning the PRISMA flowchart to make it more refined. They must also be clear on the total number of studies that were included in the whole analysis. See the authors trying to say the total number =27 studies (systematic review) then again will see 24 studies for meta-analysis (n=24→total 27 substudies). This is confusing.

I, therefore, recommend that the paper be accepted for publication.

Thank you.

David Adumbire

Reviewer 3
