# Supplementary material for: Perinatal depression and adverse child growth outcomes in low-income and middle-income countries (LMICs): A systematic review and meta-analysis
Source: PLOS Glob Public Health. 2024 Oct 28;4(10):e0003586. doi: 10.1371/journal.pgph.0003586 (PMC11516009; doi:10.1371/journal.pgph.0003586)
Supplement: Attachment — Submitted filename: Rebuttal_letter.docx [file pgph.0003586.s017.docx]

January 18, 2024

Hyelee Kim, MD, MAS, MS

Ph.D. student of Epidemiology and Translational Science Program

Department of Epidemiology & Biostatistics

University of California San Francisco

550 16th Street, Second Floor

San Francisco, CA 94158

[Hyelee.Kim@ucsf.edu](mailto:Hyelee.Kim@ucsf.edu)

Dear *PLOS Global Public Health* editors,

I am writing to summarize the changes in the resubmitted version of our manuscript (PGPH-S-23-02443), titled "Perinatal depression and adverse child growth outcomes in low- and middle-income countries (LMICs): a systematic review and meta-analysis," compared to the submitted version in June 2023 (PGPH-D-23-01052).

Firstly, as demonstrated in updated Fig1 (PDF page 55 out of 83), we updated the previous literature search up until September 2023 using the same search words, databases, and the systematic review tool (Covidence) we used in the previous version. The search words are presented in the supplementary materials (Supplement 1).

Secondly, in the previous version, among the articles we found relevant to our research questions, we needed to trim down the list to obtain the estimate from more homogeneous characteristics (final list: a total of 20 sub-studies from 17 papers). In this version, in addition to 3 papers we identified in the updated search, we included 27 papers in the systematic review and a total of 27 sub-studies from 24 papers in the meta-analysis among the studies identified in the previous version (Fig 1). Instead, we conducted various additional subgroup analyses, including time-point of outcome measurement (i.e., Fig 3A and 3B), study designs (i.e., S1 Fig A and B), and conducting sensitivity analysis.

Thirdly, in the additional subgroup analyses according to study region (i.e., Fig 4B), we found that the study region might be a meaningful factor that affects the heterogeneity of estimates. Therefore, we reconducted the meta-analyses, including the updated study list and added the estimates of the meta-regression in Table 4.

Fourthly, while our study’s outcome was child adverse growth outcomes, many studies sought the association between perinatal depression and child growth outcomes. Given the indirect relevance of the findings of those studies, we also added the summary of the studies from those studies in the S2 Table.

Hyelee Kim, MD, MAS, MS

Ph.D. student of Epidemiology and Translational Science Program

Department of Epidemiology & Biostatistics

University of California San Francisco

550 16th Street, Second Floor

San Francisco, CA 94158

[Hyelee.Kim@ucsf.edu](mailto:Hyelee.Kim@ucsf.edu)

Lastly, we added S6 Fig, a directed acyclic graph of time-varying characteristics of exposure, mediator-confounders, and outcomes, which explains why adjusting birthweight could be problematic for answering the association between perinatal depression and child adverse outcomes.

Thank you so much again for considering our paper for your journal. For further questions, please contact Dr. Hyelee Kim at [Hyelee.Kim@ucsf.edu](mailto:Hyelee.Kim@ucsf.edu).

Sincerely,

Hyelee Kim MD, MAS, MS
