# Supplementary material for: Perinatal depression and adverse child growth outcomes in low-income and middle-income countries (LMICs): A systematic review and meta-analysis
Source: PLOS Glob Public Health. 2024 Oct 28;4(10):e0003586. doi: 10.1371/journal.pgph.0003586 (PMC11516009; doi:10.1371/journal.pgph.0003586)
Supplement: Attachment — Submitted filename: Response to Reviewers.docx [file pgph.0003586.s019.docx]

**Response to Reviewers**

Dear reviewers,

Thank you so much for your keen feedback on improving our manuscript. As suggested by reviewers, we reduced the length of the article to make it compact and succinct. For journal style, we followed the PLOS ONE submission Guidelines (https://journals.plos.org/plosone/s/submission-guidelines). The reply to each reviewer is shown below.

**Reviewer #1:**

**Introduction**

Page 4. Paragraph 2 line 2….check the sentence “ there is relatively less evidence exists”.

Paragragh 3, line 5 also check the sentence “our primary interest lay understanding the temporal relationship between PND and adverse child growth outcomes”.

Page 4, line 6 what do you mean by occurring before as used in the sentence

- We edited the sentences mentioned above, as shown below.
  - *“there is relatively less evidence regarding the relationship between perinatal maternal mental health and child adverse growth outcomes in LMICs.”*
  - *“our primary interest lies in understanding the temporal relationship between PND and adverse child growth outcomes.”*
  - Deleted “occurring”

**Methods**

I find some conflicting information in the method and discussion. In the method section, I read articles included where from 2021 to 2023. But in the discussion, you mentioned that the search spanned from 2003 and 2023. Kindly reconcile.

- *We added the details of the two searches, the original search between 2000 and 2021 and the update between 2021 and 2023, to the paper, as shown below.*

*“Our initial search was from January 2000 to March 2021, and during the revision process, we updated the search until September 2023* *to include the recent publications for the past two years.”*

Furthermore, considering different tools were used in measuring depression, could it not have affected the results? I mean, they have different validity, strength of measurement and scoring. If so, it can be highlighted as a limitation of study. Some studies on PND usually include data on relationship of the PND and patient or demographic factors. Were these factored into your study?

- Thank you for the feedback. We agree with you, and it would be challenging to say that the target sensitivity or specificity of different depression measures would be consistent across the measures. Previously, we mentioned the heterogeneity in measures briefly, so we added the limitation from measures explicitly, as demonstrated in the second paragraph below.
  - *“Most of meta-analyses demonstrated substantial or considerable heterogeneity of included studies. There were limited numbers of studies for each outcome, and we observed significant variations in the characteristics of existing studies, including different measures and their thresholds, types of measure of association, features of the study population (country, region, time), and analytic approaches (selection of confounding factors).”*
  - *“**The included studies used different tools to measure PND, with different sensitivities and specificities, and this can be one plausible explanation for the observed heterogeneity.”*
- Regarding the demographic factors related to PND, we initially tried to include some of these factors in meta-regression but could not because different units and tools were applied to them. We agree that the context of demographic factors could differ according to diverse LMICs, so more studies are needed. Data harmonization through the research consortium would be a better way to maintain the consistency of measures and tools applied in diverse studies.

**Discussion**

Page 36, paragragh 1, line 10. Check the sentence and rephrase” In the Brazilian cohort study (N = 3,564), which we excluded due to timing of outcome measurement, the authors reported that mothers who scored as having PPD three times (12, 24, and 48 months).”

- *“A Brazilian cohort study (N = 3,564), which we excluded due to the timing of outcome measurement, reported heterogeneous association according to PPD duration (12, 24, and 48 months)”*

Page 37, line 11…check sentence “Similar to the issue of with wasting”.

- Deleted “Similar to the issue of with wasting”.

Also check line 13 -16.

- We edited the sentence as shown below.

*“Santos et al. (2010) reported no association between PPD and overweight among 4-year-old children whose mothers had PPD symptoms once or twice over during the measurement period (44).”*

Line 24…prioritized

Page 38 line 12, we sought,

- For the points above, we deleted the pertinent paragraph to make the paper more succinct. Thank you for pointing them out.

Line 20, 27 % started

- Edited accordingly (“*27% of PPD started before pregnancy (97)*”).

**Figures**

In the figures, I tried understanding the columns exp (ES) and % weight. There are no keys explaining them

- Added the explanations to each figure.

**Reviewer #2:** their investigation entailed meticulous searches throughout a comprehensive array of nine databases, including PubMed, EMBASE, Web of Science, CINAHL Plus, Global Health Database, Google Scholar, WHO Regional Databases, PsycINFO, and LILACS, spanning the period from January 2000 to September 2023. they placed a deliberate focus on studies that utilized well-established screening instruments or clinical interviews to gauge perinatal depression (PND) over the course of pregnancy or up to a year after childbirth. Inclusion criteria were centered on research that detailed any of four detrimental growth indicators in children under five years old, namely stunting, wasting, underweight, and overnutrition/obesity. To synthesize the findings, they integrated both risk ratios (RRs) and odds ratios (ORs) that link PND to these negative growth outcomes, employing a random-effects model to accommodate the diversity inherent in the data.

The paper is very good but very long. Researchers need to focus on the important results, narrow down the details, and transfer some tables to the appendices to make it easier for readers.

Thank you for the feedback! According to the suggestion, we trimmed down the manuscript, particularly for the Result and Discussion sections, and moved some subgroup analysis figures to supplements.

**Reviewer #3:** Perinatal depression and adverse child growth outcomes are major public health concerns in many low- and middle-income countries (LMICs). Thus, the paper is critical and it contributes to a major research and health contribution. The analyses carried out by the authors are rigorous and merit publication for the knowledge to share with the academic and health community. However, there are major revisions to be made to make the paper very firmed and readable. I have made comments in the paper which could guide authors to effect the changes.

Thank you for the keen and detailed feedback. We changed the position of the period after the reference according to the suggestion. Please check out the reply for each feedback.

- Title
  - The definition of LMICs was low-income and middle-income countries, as defined by the Word Bank, including low, lower-middle, and upper-middle countries.^[1]^^[2]^ Given the heterogeneous usage of LMICs we were also able to see (low- and middle-income countries vs. low and lower-middle-income countries), we edited the title to make it clear: low-income and middle-income countries (LMICs).
- Abstract
  - Deleting ‘adverse’ – decided to keep it since the outcome is ‘adverse’ growth outcomes, not growth outcomes.
  - Spanning data from .. – added the relevant part to the result and double-checked the numbers (N = 26,261). We used N of 1,739 for Saleh et al. (2023) since population measured height and weight overlapped.
  - Total population calculation in Stata (numbers from Table 1)

. di 171+172+265+144+147+595+242+892+278+652+798+699+200+1833+1930+1946+1961+1885+325+1124+200+300+1003+470+470+232+4025+428+1135+1739

26261

- Introduction
  - Location of references: edited accordingly.
- Method
  - Data source: supplemental sources were added to the abstract and PRISMA flowchart (Figure 1). In the previous submission process, we were supposed to submit the PRISMA checklist separately. Sorry for making mistakes in order numbers for supplements.
  - Study selection: We included 136 countries identified as LMICs by the World Bank in 2021, including Brazil and South Africa.^[3]^ We added more detail to the description of how the data extraction was performed among the two authors from the list.
  - Quality assessment: added Joshi and Raut (2019)
  - Synthesis: for the income level of meta-analysis presented in Table 4, we categorized countries as low-income, lower-middle-income, and upper-middle-income countries. We moved the relevant paragraph from the Result to the Method section. Additionally, while we assume independence of observations in the regression analysis, in the longitudinal study, the repeated observation among individuals cannot be independent of each other. We added more detail for explanation.
  - We moved the description for the meta-regression from the Result to Method section and added details for the income category.
- Result
  - According to the manuscript body formatting guidelines, we put the title and legend of figures in the middle of the manuscript.
  - South Africa and Brazil are upper-middle-income countries, so we included them in our analysis (low-income and middle-income countries).
  - Table 1: we double-checked the number of total populations. In Saleh (2023), we included 1,739 for calculation since the different outcomes (height and weight) were measured in the same population.
  - Legend for Table 1: we originally followed the PLOS manuscript body formatting guidelines but edited according to the reviewer’s suggestion.
  - Figures: according to the submission guidelines (*“Do not include figures in the main manuscript file. Each figure must be prepared and submitted as an individual file”),* we added the titles and legends figures to the manuscript and uploaded figure files separately. Please let us know if we need to add figures directly to the manuscript.
  - Publication Bias: edited as shown below.

*“According to the measure of association and outcome strata, we obtained p-values in the Egger’s tests: OR of stunting (n = 14, p = 0.241), RR of stunting (n of sub-studies = 10, p = 0.292), OR of underweight (n = 10, p = 0.403), and RR of underweight (n of sub-studies = 10, p = 0.685).”*

- Discussion
  - Thank you for providing the reference. In this qualitative study, we were able to see that social support and economic status may play a role in the development of PND (*“Three major categories were recognized as being contributing factors to cases of PND. These were financial problems, family stress/lack of social support, and problems between women and their husbands/partners.”*). We deleted the references and pertinent parts you pointed out and applied the recommended reference in another part.
  - Regarding Holm-Larsen et al. (2019), thank you so much for pointing that out. After checking out, we edited the country and prevalence of stunting accordingly.
  - Further: we intended to point out that the meaning of support from family and community and access to resources could differ according to the context in diverse LMICs, while studies on this question pay attention solely to the relationship between mother and child.
  - In the Brazilian cohort study: here, we used the number from Table I originally but changed it to the total population number used by authors.

1. Low & middle income | Data. data.worldbank.org. <https://data.worldbank.org/country/XO>
2. World Bank Classification of Low and Middle Income Countries (LMICs). EPH European Public Health Conference. <https://ephconference.eu/repository/conference/2022/LMICs%20classification%20World%20Bank%202022.pdf>
3. World Bank income groups. Our World in Data. Accessed July 2, 2024. <https://ourworldindata.org/grapher/world-bank-income-groups?time=2021>
